# Discovery of β‐nitrostyrene derivatives as potential quorum sensing inhibitors for biofilm inhibition and antivirulence factor therapeutics against *Serratia marcescens*

**DOI:** 10.1002/mlf2.12135

**Published:** 2024-09-06

**Authors:** Jiang Wang, Jingyi Yang, Pradeepraj Durairaj, Wei Wang, Dongyan Wei, Shi Tang, Haiqing Liu, Dayong Wang, Ai‐Qun Jia

**Affiliations:** ^1^ Hainan Affiliated Hospital of Hainan Medical University Hainan General Hospital Haikou China; ^2^ Center for Translational Research Shenzhen Bay Laboratory Shenzhen China; ^3^ Key Laboratory of Tropical Biological Resources of Ministry of Education, School of Pharmaceutical Sciences Hainan University Haikou China; ^4^ Hainan Branch, Shanghai Children's Medical Center, School of Medicine Shanghai Jiao Tong University Sanya China; ^5^ Present address: National High Magnetic Field Laboratory, FAMU‐FSU College of Engineering Florida State University Tallahassee Florida USA

**Keywords:** biofilms, quorum sensing, *Serratia marcescens*, virulence factors, (*E*)‐1‐methyl‐4‐(2‐nitrovinyl)benzene

## Abstract

Quorum sensing (QS) inhibition has emerged as a promising target for directed drug design, providing an appealing strategy for developing antimicrobials, particularly against infections caused by drug‐resistant pathogens. In this study, we designed and synthesized a total of 33 β‐nitrostyrene derivatives using 1‐nitro‐2‐phenylethane (NPe) as the lead compound, to target the facultative anaerobic bacterial pathogen *Serratia marcescens*. The QS‐inhibitory effects of these compounds were evaluated using *S. marcescens* NJ01 and the reporter strain *Chromobacterium violaceum* CV026. Among the 33 new β‐nitrostyrene derivatives, (*E*)‐1‐methyl‐4‐(2‐nitrovinyl)benzene (m‐NPe, compound 28) was proven to be a potent inhibitor that reduced biofilm formation of *S. marcescens* NJ01 by 79%. Scanning electron microscopy (SEM) and confocal laser scanning microscopy (CLSM) results revealed that treatment with m‐NPe (50 μg/ml) not only enhanced the susceptibility of the formed biofilms but also disrupted the architecture of biofilms by 84%. m‐NPe (50 μg/ml) decreased virulence factors in *S. marcescens* NJ01, reducing the activity of protease, prodigiosin, and extracellular polysaccharide (EPS) by 36%, 72%, and 52%, respectively. In *S. marcescens* 4547, the activities of hemolysin and EPS were reduced by 28% and 40%, respectively, outperforming the positive control, vanillic acid (VAN). The study also found that the expression levels of QS‐ and biofilm‐related genes (*flhD, fimA, fimC, sodB, bsmB, pigA, pigC*, and *shlA*) were downregulated by 1.21‐ to 2.32‐fold. Molecular dynamics analysis showed that m‐NPe could bind stably to SmaR, RhlI, RhlR, LasR, and CviR proteins in a 0.1 M sodium chloride solution. Importantly, a microscale thermophoresis (MST) test revealed that SmaR could be a target protein for the screening of a quorum sensing inhibitor (QSI) against *S. marcescens*. Overall, this study highlights the efficacy of m‐NPe in suppressing the virulence factors of *S. marcescens*, identifying it as a new potential QSI and antibiofilm agent capable of restoring or improving antimicrobial drug sensitivity.

## Impact statement

In the pursuit of developing a new class of quorum sensing inhibitor (QSIs) targeting opportunistic pathogens, we systematically designed and synthesized 33 β‐nitrostyrene derivatives. Through a thorough assessment of their QS‐inhibitory and antibiofilm formation properties, it became evident that m‐NPe stands out as a robust QSI against *Serratia marcescens*. Our findings unequivocally support the notion that m‐NPe has excellent potential for development as a highly effective QSI and antibiofilm agent, particularly in the combat against microbial infections. Furthermore, we propose that m‐NPe could be considered for clinical trials, offering the prospect of partially restoring or improving antimicrobial drug sensitivity.

## INTRODUCTION

The growing incidence of antimicrobial resistance (AMR) in the fight against significant infectious diseases poses an enormous risk to health worldwide in the post‐pandemic era, and it keeps worsening. A rise in AMR infection has become unavoidable due to the excessive use of antimicrobials which has rendered human health more vulnerable to opportunistic microorganisms^1^. This fact highlights the crucial necessity of discovering effective and efficient medicines in the event of a huge illness outbreak. The drug repurposing strategy is the process to find new applications for existing medicines or prodrugs. It can improve our readiness and fulfill our demand by using the arsenal of modern antimicrobials^2^. Most bacteria causing nosocomial infections regulate their virulence factor production by quorum sensing (QS)^3^. *Serratia marcescens*, a Gram‐negative, rod‐shaped bacterium of the *Enterobacteriaceae* family, is a well‐studied opportunistic pathogen that causes a wide range of diseases such as pneumonia, nosocomial bacteremia, or wound and urinary tract infections^4^. *S. marcescens* species can produce a wide range of N‐acyl‐homoserine lactones (AHLs), viz., C4‐HSL, C6‐HSL, 3‐oxo‐C6‐HSL, C7‐HSL, and C8‐HSL, and use QS by sensing external signals in order to regulate motility, biofilm formation, secondary metabolite production, and virulence factor production^5^. *S. marcescens* can release multiple extracellular virulence factors and, most critically, form exopolysaccharide protein polymers or biofilms^6^. Biofilms attach to biotic or abiotic surfaces to form protective barriers against the host immune system and antibacterials. Notably, *S. marcescens* produces a vital virulent factor, prodigiosin, a red pigment implicated in QS control, which plays a critical role in host invasion, survival, and pathogenicity^7^. Interference with QS systems in pathogenic bacteria has been widely studied as an effective way to manage *S. marcescens* infection^8^.

**Figure 1:**
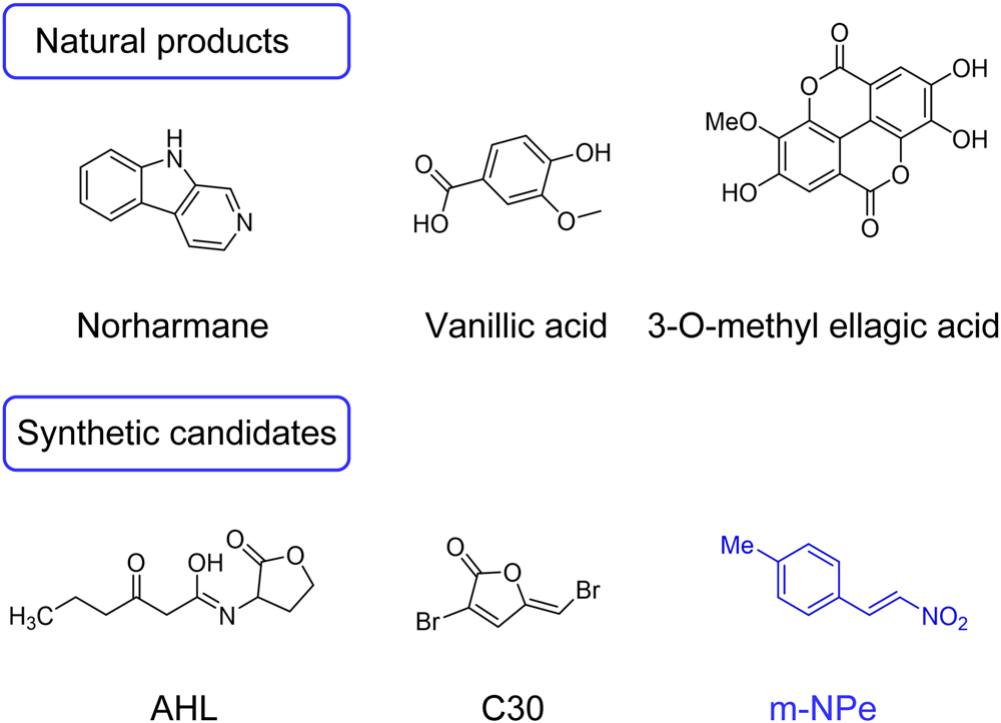
Representatives of natural products as QSIs and synthetic QSIs of *Serratia marcescens*. AHL, *N*‐acyl‐homoserine lactone; QSIs, quorum sensing inhibitors.

To date, a number of natural compounds, including alkaloids, organic acids, and their synthetic derivatives, have been identified as quorum sensing inhibitors (QSIs) and antibiofilm agents^9^, ^10^, ^11^, ^12^. The majority of the identified QSIs mimic AHL molecules or are originated from natural sources (Figure 1)^13^, ^14^, ^15^, and these compounds are often created by substituting the head group or linker of AHL molecules. Nonetheless, AHL molecules and their highly active analogs are usually unstable and prone to ring‐opening in vivo^16^. Interestingly, 10 µM of C30 (Figure 1), a synthetic halogenated furanone derivative, has been found to reduce the production of virulence factor and biofilm formation in drug‐resistant bacteria *Pseudomonas aeruginosa* PAO1, also improve the sensitivity to antimicrobials, and effectively remove the bacteria in the lungs of mice^17^. Even though C30 is relatively stable, its toxicity has been reported to be very high in HeLa cancer cells^18^. Therefore, to restore and/or improve drug sensitivity for drug‐resistant organisms, it is imperative to discover new QSIs and antibiofilm compounds that are both highly effective and safe.

For the aforementioned reason, a significant bioactive natural product, 1‐nitro‐2‐phenylethane (NPe), was first isolated from *Aniba canelilla* ^19^. β‐nitrostyrenes were reported to have antimicrobial activities^20^. According to a recent report, analogous compounds with nitroolefin structures that contain furan ring groups can enhance antibacterial activity by targeting QS^21^, and 2‐(2‐methyl‐2‐nitrovinyl)furan can interfere with the *Staphylococcus aureus* Agr QS system and amplified the effects of fusidic acid on biofilms^21^. Nevertheless, these studies were limited to the preliminary stage, and their QS‐inhibitory effects were moderate. It is necessary to synthesize new β‐nitrostyrene drugs that are highly selective as QSIs to treat infections caused by *S. marcescens* effectively.

**Figure 2:**
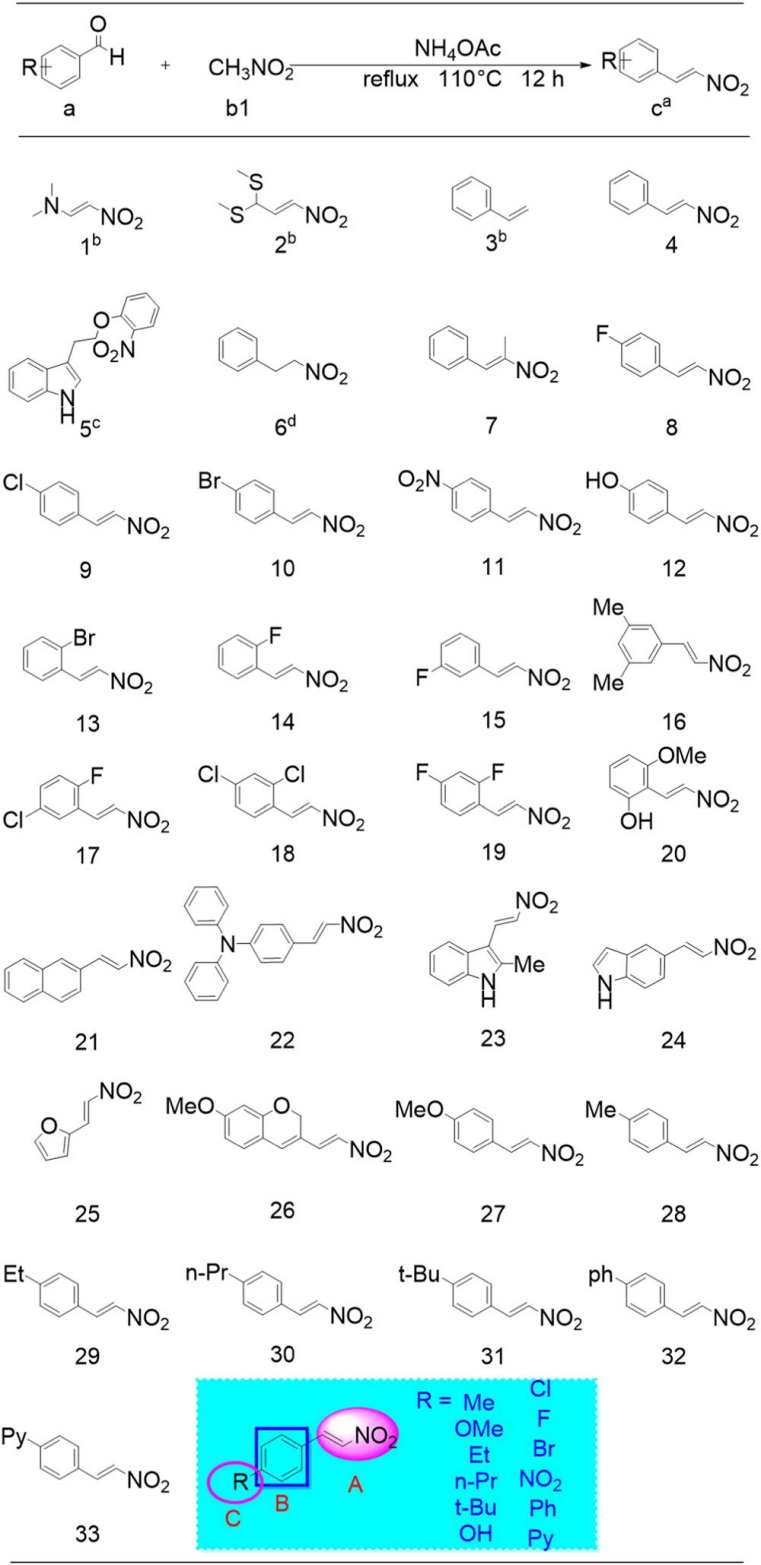
Design strategy and synthesized β‐nitrostyrene derivatives for QSIs against *Serratia marcescens*. The superscripts lowercase letters a, b, c, and d represent the conditions: ^a^Benzaldehyde a, ammonium acetate, dry nitromethane b1 (5 ml) at 110°C reflux for 5 h; ^b^compounds were purchased from Sigma‐Aldrich in United States; ^c^2‐nitrophenol, DIPEA, 3‐(2‐bromoethyl)‐1*H*‐indole, in dichloromethane, yielded the product, ^d^based on compound 4, and sodium tetrahydroborate reduction. DIPEA, *N*,*N*‐Diisopropylethylamine.

In the study, we applied a systematic strategy to design, synthesize, and evaluate β‐nitrostyrene candidates (designated as parts A, B, and C, as illustrated in Figure 2) as a new class of QSIs for *S. marcescens*. Initial research revealed that NPe showed QS inhibition against *C. violaceum* CV026 and *S. marcescens* NJ01 (Figures S1–S2). Building upon these findings, we explored the structure–activity relationship (SAR) of NPe derivatives using a ligand‐based design and synthesis approach. To further enhance the QS‐inhibitory effects against *S. marcescens*, we optimized the structure of NPe and synthesized 33 β‐nitrostyrene derivatives (designated as compounds 1–33). The QS activity of the synthesized compounds was evaluated by measuring the expression levels of virulence factors in *S. marcescens* NJ01, including prodigiosin, and performing biofilm formation inhibition assays. The results show that these compounds have a distinctive potential to competitively quench QS signaling, providing prospective ways for fighting microbial infections.

## RESULTS

### Synthesis of β‐nitrostyrene derivatives

To synthesize a highly effective QSI and efficient antibiofilm agent, a β‐nitrostyrene‐bearing nitroolefin with a heterocyclic ring group was designed, synthesized, and studied for its potential to inhibit QS. Applying potential SAR, the β‐nitrostyrene structure was divided into three components (parts A, B, and C) (Figure 2). Initially, the importance of the nitroolefin group in component A to the compound's activity was identified. Further, our projections show that the benzene ring in part B outperforms the natural heterocyclic aromatic ring. Lastly, in part C, modifications were made by introducing electron‐withdrawing or electron‐donating groups onto the benzene ring (or other groups), as well as adjusting the position or number of these substituents. Here, NPe was used as a lead compound to design and synthesize 33 NPe derivatives, and their QS‐inhibitory and antibiofilm formation capabilities were evaluated.

Specifically, the β‐nitrostyrene derivatives were synthesized as shown in Scheme S3 in the Supporting Information. Target product C was synthesized through the condensation reaction of several substituted benzaldehydes with nitromethane (both of which are commercially available). The majority of the target products C were observed in the form of light‐yellow solids. The key target compounds, 1, 2, and 3, were obtained from commercial sources. The key target compound 5 was synthesized by a condensation reaction of 2‐nitrophenol with 3‐(2‐bromoethyl)‐1*H*‐indole (both of which are commercially available). The key target compound 6 was synthesized through a reduction reaction based on compound 4. We provided detailed characterization data, including ^1^H NMR,^13^C NMR, and HRMS of all the compounds utilized in this study can be found in the Supporting Information section. Interestingly, the yields of the final target compounds were approximately 60% or higher. The β‐nitrostyrene derivatives used for testing QS‐inhibiting activities are listed in Figure 2. Detailed information about their chemical synthesis, identification, and their QS‐inhibitory activities is presented in the Supporting Information section.

### Screening of QS‐inhibitory β‐nitrostyrene derivatives

**Figure 3:**
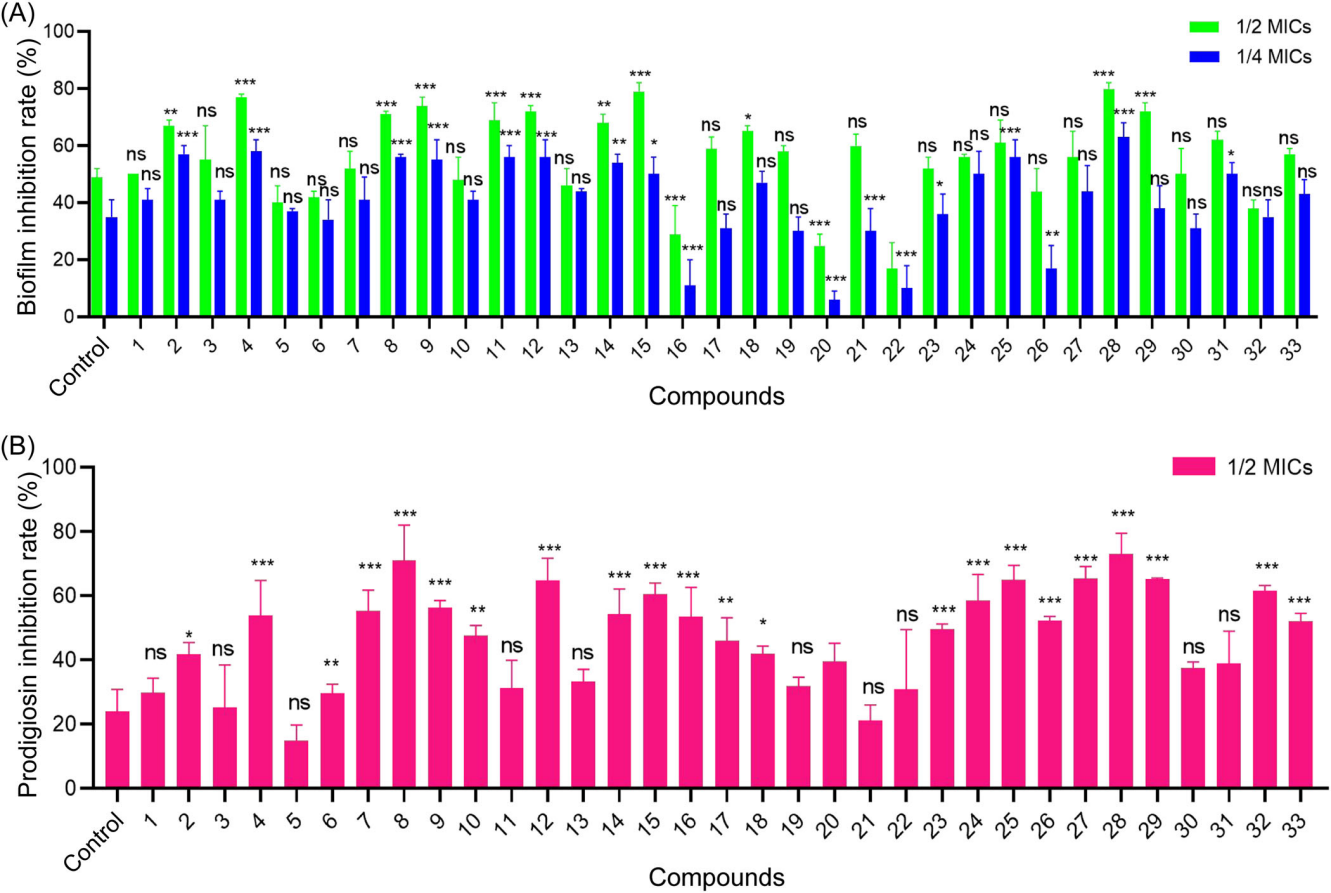
Effects of β‐nitrostyrene derivatives on biofilm and prodigiosin inhibition on *Serratia marcescens* NJ01 at subminimal inhibitory concentrations (MICs). (A) Effects of β‐nitrostyrene derivatives on biofilm inhibition on *S. marcescens* NJ01 at 1/2 MICs and 1/4 MICs. (B) Effects of β‐nitrostyrene derivatives on prodigiosin inhibition of *S. marcescens* NJ01 at 1/2 MICs. Compounds were dissolved in dimethyl sulfoxide, and the vanillic acid (VAN) was used as a positive control. All the data are represented with the mean and standard deviation of three independent experiments. The error bars represent the standard deviation of three biological replicates. Statistical differences were determined by analysis of variance, followed by the Tukey–Kramer test. *p* Values derived from these comparisons are highlighted with asterisks (*p < 0.05, **p < 0.01, and ***p < 0.001 versus the VAN control. ns, no significance, *p* > 0.05).

First, we assessed the QS‐inhibitory effects of β‐nitrostyrene derivatives on *C. violaceum* CV026, *S. marcescens* NJ01, and *S. marcescens* 4547. The results indicated that compounds 4, 9, 12, and 28 exhibited QS‐inhibitory effects on *C. violaceum* and *S. marcescens* (Figures S1–S4). Next, we investigated the minimum inhibitory concentrations (MICs) of all the synthesized β‐nitrostyrene derivatives (Table S1) and determined their inhibitory effects on biofilm formation of *S. marcescens* NJ01. Vanillic acid (VAN) from natural products was used as a positive control based on its effectiveness as a QSI against *S. marcescens* ^15^. The results showed concentration‐dependent inhibitory effects of the compounds on biofilm formation (Figure 3A). The analysis clearly demonstrated that (*E*)‐1‐methyl‐4‐(2‐nitrovinyl)benzene (m‐NPe, compound 28) had the most potent inhibitory effect on *S. marcescens* NJ01, with about 80% repression at 1/2 MIC. Based on these discoveries, m‐NPe was chosen as the target compound for subsequent investigation. Additional information is provided in Table S2.

### Inhibitory effects of β‐nitrostyrene derivatives on prodigiosin production

Next, we assessed the inhibitory efficiency of β‐nitrostyrene derivatives on prodigiosin, one of the critical virulent factors of *S. marcescens* ^22^, which may play a crucial role in pathogen invasion, survival, and pathogenicity. β‐nitrostyrene derivatives at 1/2 MICs significantly decreased the levels of prodigiosin (Figure 3B). The β‐nitrostyrene‐derivative compounds 4, 7, 8, 9, 12, 14, 15, 16, 24, 25, 26, 27, 28, 29, 32, and 33 decreased prodigiosin levels by more than 50% compared to the positive control VAN (24%). The β‐nitrostyrene derivatives 8, 28, and 29 showed substantial prodigiosin inhibition rates of 71%, 73%, and 65%, respectively. Clearly, m‐NPe not only acts as a potent QSI against *S. marcescens* (Figure 1) but it also has the ability to partially restore or boost antimicrobial drug sensitivity—an important factor in clinical administration. Additional information is provided in Table S3.

### Inhibitory effects of β‐nitrostyrene derivatives on biofilm formation

In this section, we investigated the inhibitory effects of the 33 newly synthesized β‐nitrostyrene derivatives on *S. marcescens* biofilm formation at 1/4 MICs (Figure 3A) and carried out the SAR analysis. First, we hypothesized that nitroolefin is essential for biofilm inhibition activity based on a previous study^20^. In comparison to nitroolefin, compound 3 (41%, biofilm inhibition rate) lacks the nitro structure, while compound 6 (34%) lacks the olefin structure. These compounds showed less inhibitory effects or were almost ineffective when compared to the lead compound 4 (58%). This result supports the hypothesis that the part A nitroolefin group is crucial for inhibiting biofilm formation. Further evidence that none of the substituents on the nitroolefin group are appropriate comes from compound 7 (41%), which had methyl substituents on the nitroolefin and likewise displayed reduced inhibitory effects. These results suggest that additional modifications in part B or C might be necessary and ought to be the main focus of future investigations.

Second, based on the results of compounds 21 (30%), 22 (10%), 23 (36%), 24 (50%), and 25 (57%) bearing the different substituents, we discovered that the compounds substituted with heterocycles, such as naphthalene ring (21), diphenylamine ring (22), indole ring (23, 24), and furan ring (25), were less effective than those substituted with benzene ring such as the lead compound 4 (58%). Furthermore, compounds 1 (41%) and 2 (57%) that have alkane substitutions were less effective than the lead compound 4. Compounds 5 (37%) and 26 (17%) also have fewer biofilm‐inhibitory effects than the lead compound 4, indicating that β‐nitrostyrene is the major skeleton structure.

Finally, in order to create a highly active molecule, we investigated the effects of substituents on the activity of the benzene ring in part B. The comparison of compounds 8 (56%), 14 (54%), and 15 (50%) showed that para‐substitution on the benzene ring has a greater impact than ortho‐ or meta‐positions, maybe because of the steric effects. This confirms that the para‐substitution of benzene is a dominant position. In part C, we found that the order of inhibition is F > NO_2_ > Cl > Br based on the results of compounds 8 (56%), 9 (55%), 10 (41%), and 11 (56%), which have different benzene ring substituents. Furthermore, the results of compounds 12 (56%), 27 (44%), 28 (63%), 29 (38%), 30 (31%), 31 (50%), 32 (35%), and 33 (43%), carrying different benzene ring substituents, revealed that the sequence of inhibition is Me > OH > *t*‐Bu > OMe > Py > Ph > Et > Pro. Compound 16 (11%) with different benzene ring double substitutions, on the other hand, showed weaker inhibitory effects. All of these findings suggest that the para‐methyl substitution of benzene comprises a potentially dominant structure.

It is suggested from the SAR analysis that parts A, B, and C are most closely linked to the biofilm inhibition properties of β‐nitrostyrene derivatives. To be more precise, the nitroolefin group in part A was necessary for the action, and it is not appropriate to be substituted. In part B, the benzene ring is better suited to the β‐nitrostyrene derivatives' biofilm‐inhibitory properties, and the para‐substitution of benzene rings results in increased activity. We found that the methyl substitution of benzene is important for part C. Lastly, we propose that compound 28 (m‐NPe, 63%), nitroolefin, which has a benzene ring and a p‐methyl group, is the best option for biofilm inhibition investigations against *S. marcescens* based on our exhaustive examination.

### Growth profiles of *S. marcescens* and effect of m‐NPe on AHL levels

The MIC of m‐NPe was determined to be 100 μg/ml. The growth profiles of *S. marcescens* NJ01 (Figure S5A) and *S. marcescens* 4547 (Figure S5B) showed that m‐NPe did not significantly suppress their growth in the concentration range of 12.5 to 50 μg/ml. The putative anti‐QS activity of m‐NPe was evaluated by measuring the levels of AHLs produced by *S. marcescens* NJ01. AHLs in the culture supernatants were measured by liquid chromatography‐tandem mass spectrometry (LC‐MS/MS) analysis, with a focus on C6‐HSL. A significant reduction in C6‐HSL peak heights and areas after exposure to m‐NPe at different doses (12.5–50 μg/ml) was found. Detailed information is provided in the Supporting Information section. Quantitative analysis showed that C6‐HSL levels were reduced by about 17%, 27%, and 31%, respectively, following m‐NPe treatments at doses of 12.5, 25, and 50 μg/ml, in comparison to the control (Figure S6). Notably, efforts to determine how m‐NPe therapy affected C4‐HSL levels were not successful. All of these results point to the possibility that m‐NPe has anti‐QS activity due to its disruption of AHL synthesis.

### Effects of m‐NPe on biofilm formation and destruction

**Figure 4:**
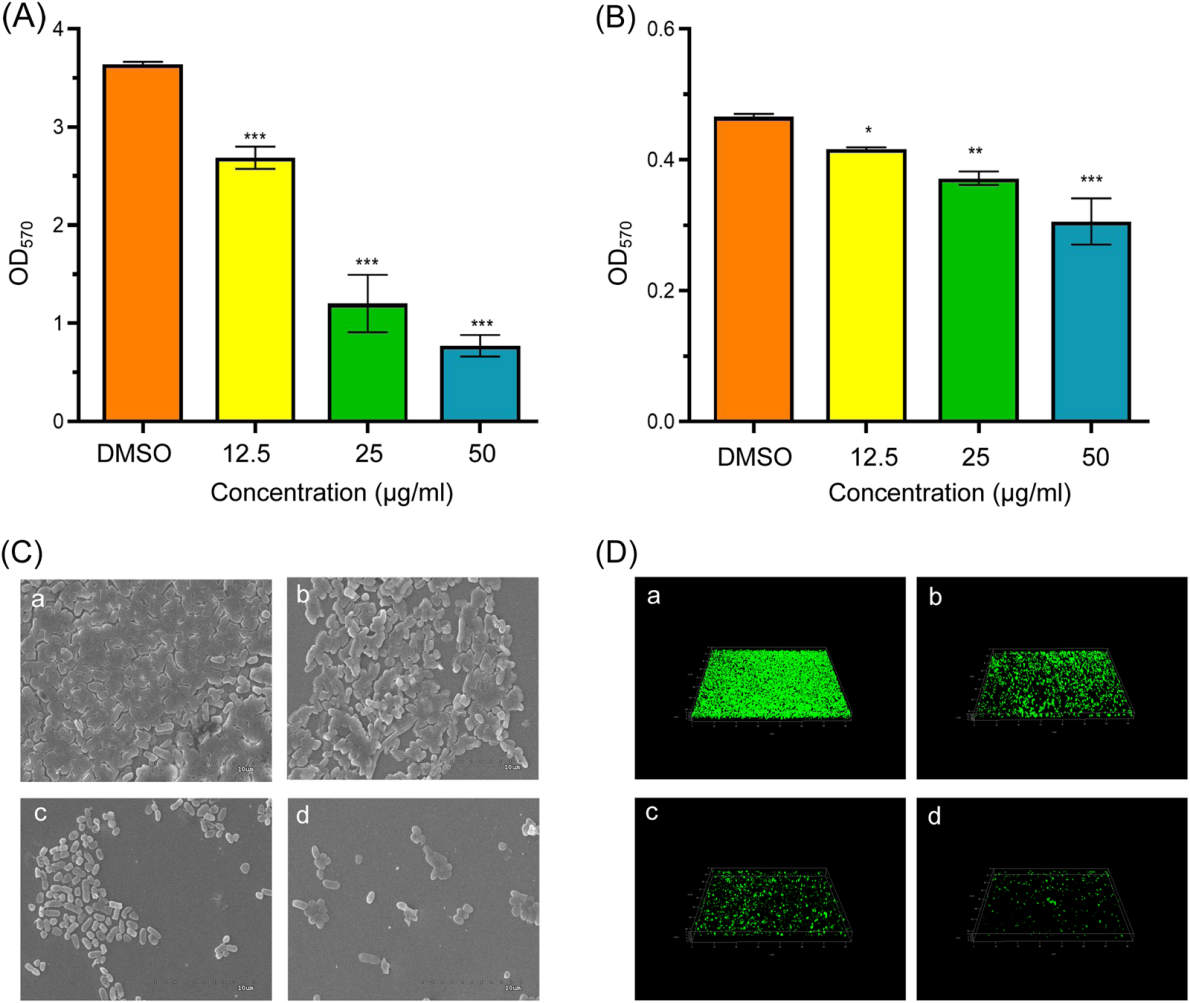
Inhibitory effects of (*E*)‐1‐methyl‐4‐(2‐nitrovinyl)benzene (m‐NPe) on biofilm formation of *Serratia marcescens*. (A, B) Inhibitory effects of m‐NPe (50, 25, and 12.5 μg/ml) on biofilm formation of *S. marcescens* NJ01 (A) and *S. marcescens* 4547 (B). Dimethyl sulfoxide (DMSO) was used as a negative control. (C, D) Scanning electron microscopy images (C) and confocal laser scanning microscopy images (D) of *S. marcescens* NJ01 treated with DMSO (a), 12.5 μg/ml (b), 25 μg/ml (c), and 50 μg/ml (d) of m‐NPe. All the data represent the means and standard deviations of three independent experiments. Statistical differences were determined by analysis of variance, followed by the Tukey–Kramer test (*p < 0.05, **p < 0.01, and ***p < 0.001 vs. the DMSO control).

**Figure 5:**
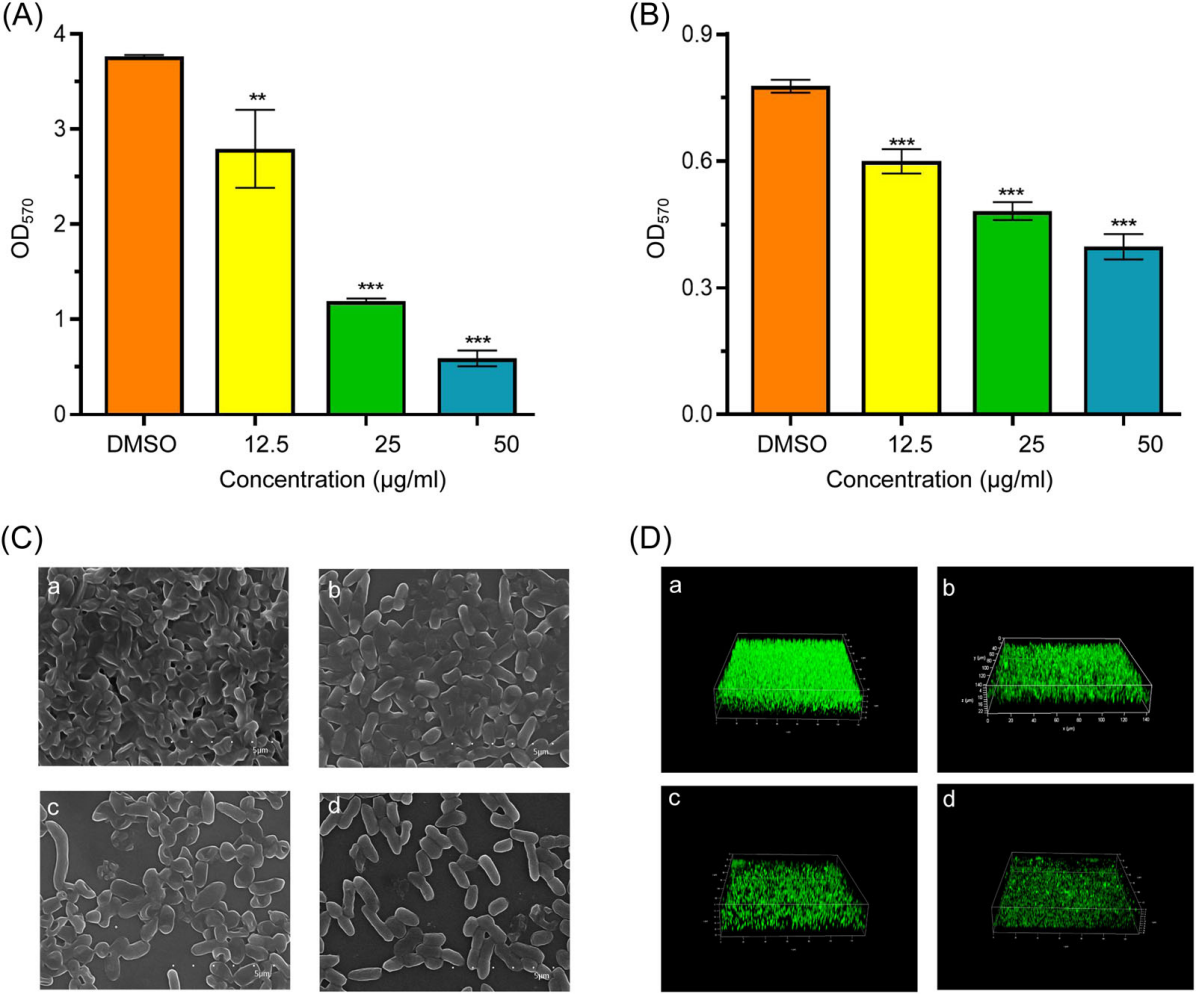
Effects of m‐NPe on destruction of formed biofilms of *Serratia marcescens*. (A, B) Destructive effects of m‐NPe (50, 25, and 12.5 μg/ml) on formed biofilm of *S. marcescens* NJ01 (A) and *S. marcescens* 4547 (B). (C, D) Scanning electron microscopy images (C) and Confocal laser scanning microscopy images (D) of *S. marcescens* NJ01 treated with DMSO (a), 12.5 μg/ml (b), 25 μg/ml (c), and 50 μg/ml (d) of m‐NPe. All the data represent the means and standard deviations of three independent experiments. Statistical differences were determined by analysis of variance, followed by the Tukey–Kramer test (**p < 0.01 and ***p < 0.001 vs. the DMSO control).

Using the crystal violet assay, the inhibitory effects of m‐NPe on biofilm formation and destruction were examined. It was found that m‐NPe at 12.5, 25, and 50 μg/ml greatly decreased the biofilm formation of *S. marcescens* NJ01 by 26%, 67%, and 79%, respectively (Figure 4A). Compared to the DMSO control, a notable suppression was actually observed in m‐NPe treatment group. Similarly, m‐NPe suppressed the biofilm formation of *S. marcescens* 4547 by 11%, 20%, and 34%, respectively (Figure 4B). Likewise, the destructive effect of m‐NPe (12.5, 25, and 50 μg/ml) on formed biofilms was examined; the m‐NPe treatments resulted in remarkable biofilm repression in each case. The biofilm reduction rates in *S. marcescens* NJ01 were 26%, 68%, and 84% (Figure 5A), while those in *S. marcescens* 4547 were 23%, 38%, and 49% (Figure 5B). Scanning electron microscopy (SEM) (Figures 4C and 5C) and confocal laser scanning microscopy (CLSM) (Figures 4D and 5D) were used to evaluate m‐NPe's inhibitory potential against biofilm. SEM images of biofilm in the blank DMSO group revealed a dense and network system linked by fibrous features, whereas the SEM and CLSM images after m‐NPe treatment (50 μg/ml) showed a significant reduction in biofilm biomass with a scattered appearance.

### Suppression of virulence factors after m‐NPe treatments

**Figure 6:**
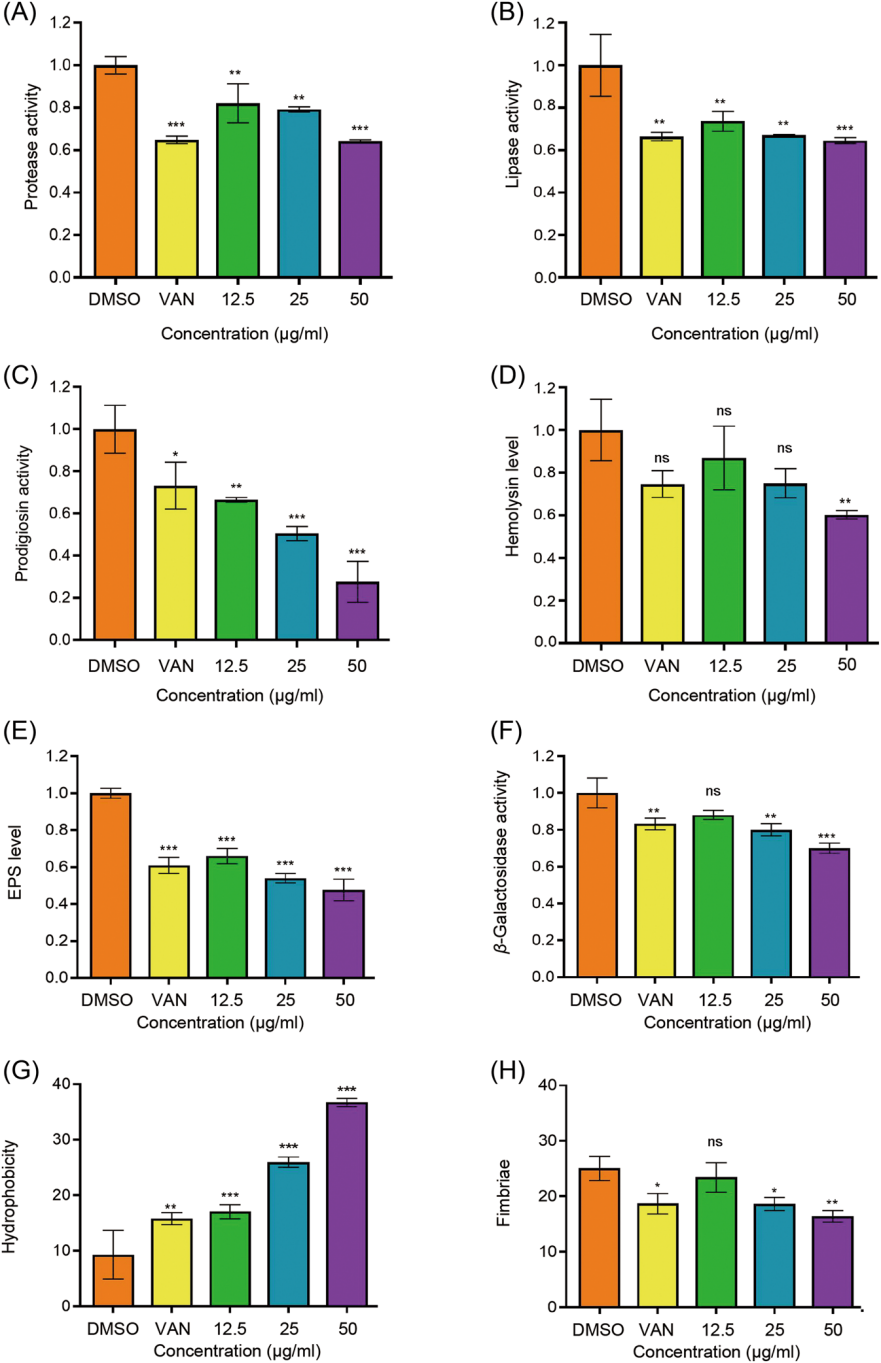
Inhibitory effects of m‐NPe on virulence factor production of *Serratia marcescens* NJ01. (A) Protease activities. (B) Lipase activities. (C) Prodigiosin activities. (D) Hemolysin levels. (E) Extracellular polysaccharide (EPS) levels. (F) β‐Galactosidase activities. (G) Fimbriae activities. (H) Hydrophobicity activities. Overnight *S. marcescens* NJ01 cultures were added to LB broth (1:100, v/v) supplemented with m‐NPe at increasing concentrations (12.5–50 μg/ml) cultivated at 28°C and 180 rpm. DMSO and VAN (50 μg/ml) served as the negative and positive controls, respectively. After incubation, 1 ml of each culture was centrifuged for 5 min at 4°C. The supernatants were stored at −20°C for further analysis. All the data represent the means and standard deviations of three independent experiments. Statistical differences were determined by analysis of variance, followed by the Tukey–Kramer test (*p < 0.05, **p < 0.01, and ***p < 0.001 vs. the negative control. ns, no significance, *p* > 0.05).

**Figure 7:**
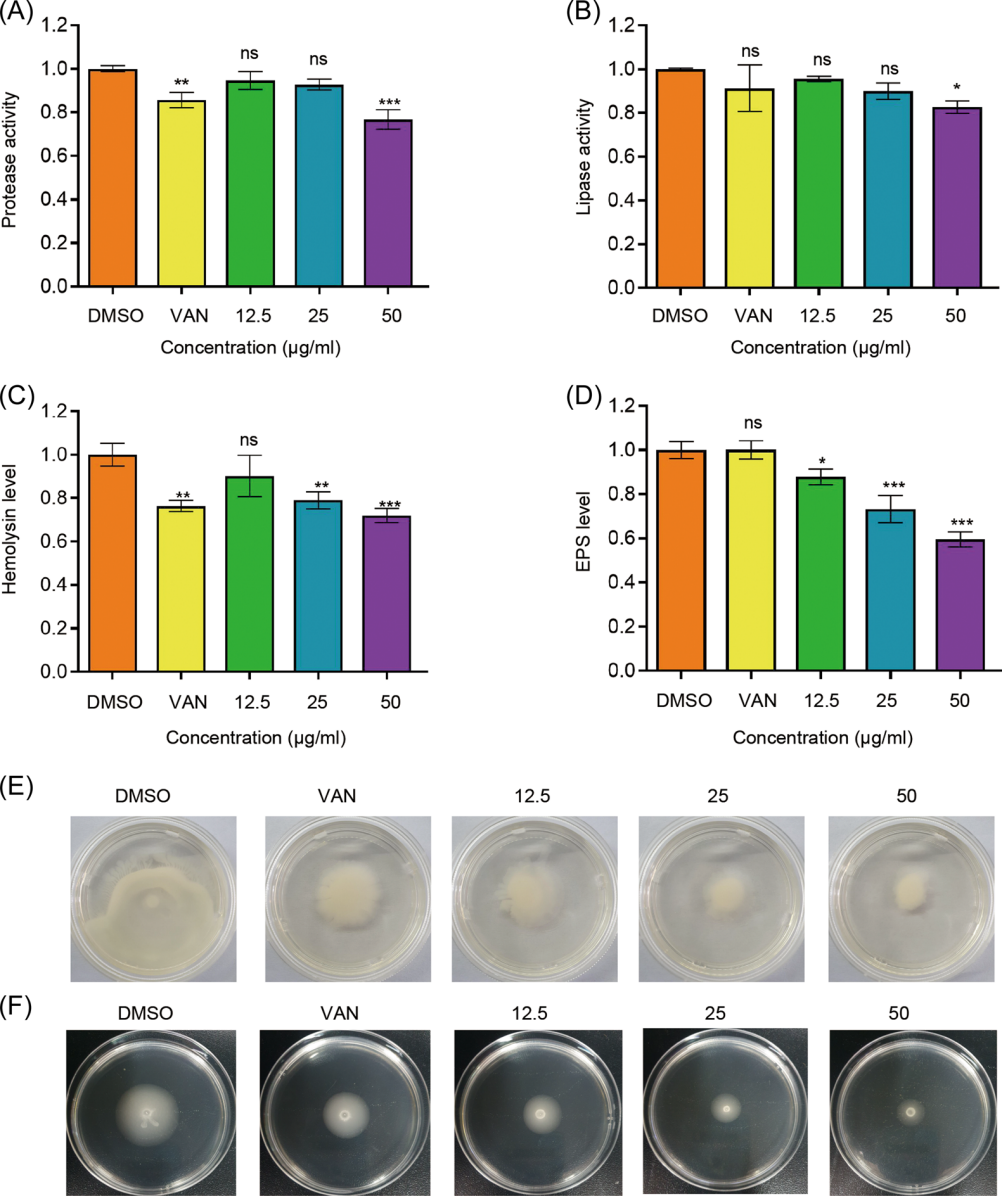
Inhibitory effects of m‐NPe on virulence factor production of *Serratia marcescens* 4547. (A) Protease activities. (B) Lipase activities. (C) Hemolysin levels. (D) EPS levels. (E, F) Swarming and swimming motilities, respectively, treated with DMSO, VAN (50 μg/mL), and m‐NPe (12.5, 25, and 50 μg/ml). Overnight *S. marcescens* NJ01 cultures were added to LB broth (1:100, v/v) supplemented with m‐NPe at increasing concentrations (12.5‐50 μg/ml) cultivated at 28°C and 180 rpm. DMSO and VAN (50 μg/ml) served as the negative and positive controls, respectively. After incubation, 1 ml of each culture was centrifuged for 5 min at 4°C. The supernatants were stored at −20°C for further analysis. All the data represent the means and standard deviations of three independent experiments. Statistical differences were determined by ANOVA, followed by the Tukey–Kramer test (*p < 0.05, **p < 0.01, and ***p < 0.001 vs. the negative control. ns, no significance, *p* > 0.05).

In this study, we discovered that m‐NPe may suppress virulence factors in both *S. marcescens* NJ01 (Figure 6) and *S. marcescens* 4547 (Figure 7). (i) Effects of m‐NPe on protease and lipase production: Treatment with m‐NPe at 50 μg/ml reduced protease activity by 36% in *S. marcescens* NJ01 compared to the control group (Figure 6A). Similarly, m‐NPe (50 μg/ml) suppressed the protease activity of *S. marcescens* 4547 by 23%, significantly higher than the positive control VAN (Figure 7A). Lipolytic enzymes are known to be involved in the decomposition of the phospholipid bilayer and influencing signaling pathways in host cells^15^. Lipase activity in *S. marcescens* NJ01 reduced by 36% and 34% (Figure 6B), respectively, upon treatments with m‐NPe and VAN (50 μg/ml), while in *S. marcescens* 4547, it reduced by 17% and 9%, respectively (Figure 7B). (ii) Effects of m‐NPe on prodigiosin and hemolysin production: Hemolysin, like prodigiosin, is also an important virulence factor by *S. marcescens* controls^23^. In the experiments, we examined how m‐NPe (50 μg/ml) affects prodigiosin and hemolysin production. As expected, m‐NPe inhibited prodigiosin activity by 72%, which was higher than that of the positive control VAN (23%) (Figure 6C). Similarly, m‐NPe inhibited hemolysin in *S. marcescens* NJ01 (Figure 6D) and S. *marcescens* 4547 (Figure 7C) by 40% (25% by VAN) and 28% (24% by VAN), respectively. (iii) Impact of m‐NPe on extracellular polysaccharide (EPS) production: Extracellular polymeric substances are highly hydrated polymers that play a critical role in biofilms by maintaining cohesion, acquiring nutrition, and preventing antimicrobials from entering cells^23^. Following m‐NPe treatment at 50 μg/ml, EPS generation in *S. marcescens* NJ01 decreased by 52%, while the positive control only reached 39% (Figure 6E). When *S. marcescens* 4547 was treated with m‐NPe at 50 μg/ml, its EPS production was reduced by up to 40% (Figure 7D). (iv) Effect of m‐NPe on β‐Galactosidase production: β‐Galactosidase is required for the opportunistic pathogen to survive on a host organism and cause tissue maceration^8^. However, some bacterial species have an organismal QS defense mechanism that drives the pathogens to produce a large amount of extracellular enzymes, such as β‐Galactosidase^24^. In the present research, m‐NPe at 50 μg/ml inhibited β‐Galactosidase activity by 30%, which was higher than the positive control VAN (17%) (Figure 6F). (v) Influence of m‐NPe on surface hydrophobicity: Bacteria's surface hydrophobicity and automatic aggregation ability are mostly determined by the number of nonpolar groups on their surface and are related to the structure of surface proteins, fimbriae, lipoteichoic acid, and capsule^25^. Upon 50 μg/ml of m‐NPe treatment, *S. marcescens* exhibited a surface hydrophobicity of 37%, which is much higher than that of the positive control VAN (16%) and negative control DMSO (9%) (Figure 6G). (vi) Impact of m‐NPe on fimbria‐mediated yeast agglutination and susceptibility to H_2_O_2_: In *S. marcescens*, the transcriptional regulator OxyR is required for the regulation of the oxidative stress response and also has an impact on the early phases of biofilm formation via the modulation of type I fimbria production^26^. We examined the effects of m‐NPe on fimbria‐mediated yeast agglutination. In the presence of 50 μg/ml of m‐NPe, *S. marcescens* NJ01 showed 16% agglutination, which is lower than both the positive control VAN (19%) and the negative control DMSO (25%) (Figure 6H). Furthermore, we found that the sensitivities of m‐NPe‐treated *S. marcescens* NJ01 to H_2_O_2_ were higher than those of the controls^27^ (Figure S7A). At a concentration of 12.5 μg/ml for m‐NPe, it can increase H_2_O_2_ production. The results indicated that *S. marcescens* NJ01 underwent severe oxidative stress upon exposure to m‐NPe. (vii) Impact of m‐NPe on swarming and swimming motility: Swarming and swimming in *S. marcescens* could also be affected by the QS system^28^, ^29^. Recently, it was reported that swimming is mostly regulated by the flagellar master operon (*flhDCSm*), the flagellin structural gene (*hagSm*), and the nuclease gene (*nucASm*) in *S. marcescens* ^28^. Besides, it was reported that swimming could be primarily affected by the *RsmA* gene in the QS system of *S. marcescens* ^29^. QSIs can also suppress motility and swarming. As a consequence, we investigated the effects of m‐NPe on swarming motility in *S. marcescens* NJ01 (Figure S7B) and *S. marcescens* 4547 (Figure 7E), and found that the results were comparable to prodigiosin. A similar pattern was also seen in swimming motility (Figure 7F).

### Expression of QS and biofilm‐related genes under m‐NPe treatments

**Figure 8:**
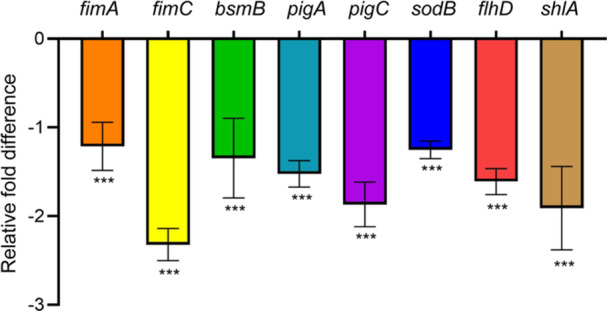
Relative fold difference in the expression levels of quorum sensing and the biofilm‐related genes of *Serratia marcescens* NJ01 after treatment with m‐NPe. The results are expressed as means ± SD (*n* = 3). Statistical differences were determined by analysis of variance, followed by the Tukey–Kramer test (***p < 0.001 vs. the *rplU* control).

To evaluate the effects of m‐NPe on QS‐mediated genes *sodB*, *fimA*, *bsmB, fimC, pigA, flhD, pigC*, and *shlA* in *S. marcescens* NJ01, we performed the reverse transcription‐quantitative real‐time polymerase chain reaction (RT‐qPCR) test (Figure 8). Downregulation was observed in genes *flhD, fimA*, and *fimC*, which are related to motility, fimbria production, and adherence, with reduction of 1.61‐, 1.21‐, and 2.32‐fold, respectively. Genes *sodB* and *bsmB*, which are responsible for biofilm formation, were downregulated by 1.25‐ and 1.35‐fold, respectively. Similarly, genes *pigA, pigC*, and *shlA* involved in the biosynthesis of prodigiosin and hemolysin were downregulated by 1.52‐, 1.87‐, and 1.91‐fold, respectively.

### Molecular docking, MD, and MST analysis of m‐NPe

In order to gain a better understanding of the anti‐virulence and antibiofilm activities of m‐NPe, molecular docking was performed. In light of the reporter strain used for QSI screening, we first performed molecular docking of m‐NPe with targeting proteins in *C. violaceum* CV026. CviR is a key receptor in *C. violaceum* CV026^31^, with the ligand‐binding domain (LBD) and the DNA‐binding domain (DBD) being two critical domains within the CviR QS system^32^. The benzene ring of m‐NPe interacts with the Leu100 of CviR to form a π‐type hydrogen bond. The benzene ring interacts with CviR's amino acid residues Phe 126 and Tyr 80 via π–π stacking and π–π T‐shaped interactions (Figure S8E). Next, we investigated the two major transcriptional activators (SmaR and LasR) of the *S. marcescens* QS system. Our results indicated that the nitryl group of m‐NPe forms a hydrogen bond with the Ser124 residue of SmaR (Figure S8A). In LasR, the m‐NPe interacts with Arg135 and Ala145 via hydrophobic interactions. Besides, the benzene ring interacts with Val150 to form a π‐type hydrogen bond (Figure S8D). Following this, we investigated two transcriptional regulators (Rh1I and Rh1R) in the *S. marcescens* QS systems. In RhlI, the nitryl group of m‐NPe forms a hydrogen bond with the Arg61 residue. Herein, the benzene ring interacts with Tyr56 and Trp88 of RhlI through a π‐type interaction (Figure S8B). In contrast, the nitryl group of m‐NPe forms a hydrogen bond with the Ser285 residue of RhlR (Figure S8C).

**Figure 9:**
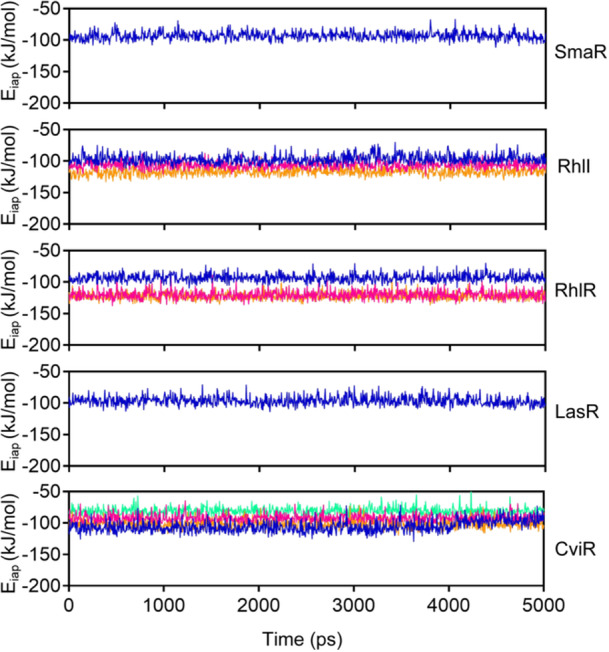
Interaction potential energy (E_iap_) profiles of m‐NPe with target proteins. Interaction potential energy (E_iap_) profiles of molecular dynamic simulations (MD) studies for target proteins bound with m‐NPe. Time‐dependent plots for E_iap_ of atomic positions, m‐NPe for the H‐bonds, H‐π interaction, and π‐π interaction with target proteins: SmaR, RhlI, RhlR, LasR, and CviR.

To analyze binding stability, MD simulations of m–NPe complexes comprising different target proteins participating in QS and biofilm formation were performed. Based on our molecular docking analysis, five proteins (SmaR, RhlI, RhlR, LasR, and CviR) were selected as target proteins for MD simulations. During the simulations, the root mean square deviation (RMSD) and interaction potential energy (E_iap_), which is the sum of Lennards–Jones and Coulombic potential energy, were calculated. The RMSD values of the heavy atom positions of ligands bound to target proteins were calculated to validate their binding stability to respective proteins (Figure S9). We found that the RMSD for all ligands binding to proteins remained less than 0.25 nm. For example, the RMSD of the SmaR–m–NPe complex was calculated to be 0.09 ± 0.028 nm (Figure S9A). After brief initial equilibration, the RMSD remained constant under all conditions, demonstrating that the ligands bind to target proteins in a persistent manner. In addition to the RMSD analysis, we also calculated E_iap_ between the proteins and ligands, which ranged from −80 to −120 kJ/mol (Figure 9). Specifically, the E_iap_ for the SmaR–m–NPe complex was calculated to be −93.31 ± 6.327 kJ/mol (Figure 9). Across all conditions, we found that the E_iap_ reached equilibrium and stabilized after a few picoseconds, further confirming the robustness and stability of the binding interactions between SmaR and m‐NPe.

Following the determination of m‐NPe binding stabilities with several target proteins via MD simulations, we used MST to demonstrate the interaction between m‐NPe and the target proteins. Initially, m‐NPe and the co‐crystallized natural ligand C4‐HSL were docked into the active site of the SmaR protein during the docking study^33^. SmaR is a common QSI target protein that binds to C4‐HSL and C6‐HSL to inhibit *S. marcescens* ^33^, ^34^. Therefore, the identification of target proteins is critical in our study. We applied the MST assay with pure recombinant SmaR protein and found that it binds to m‐NPe with a predicted dissociation constant (*K*_d_) of 38.95 μM (Figure S10). However, our study of RhlI, RhlR, and LasR proteins for m‐NPe using the MST test produced negative results, indicating a lack of binding. This highlights the necessity of m‐NPe binding for protein–compound interactions. As a result, we proposed that SmaR should be considered as a target protein in the screening of QSI against *S. marcescens*.

### Cytotoxicity assessment of m‐NPe

Based on the positive results of our in vitro experiments, we conducted additional research to determine the effects of m‐NPe on the mortality of *Tenebrio molitor* larvae. Notably, larvae injected with *S. marcescens* NJ01 died early in the incubation period, with nearly 80% perishing by the third incubation phase. In contrast, larvae treated with m‐NPe had a significantly higher survival rate, approaching 70% (Figure S11B). Similar effects were seen with larvae injected with *S. marcescens* 4547 (Figure S11A). Furthermore, we observed that the mortality of larvae treated with 12.5–50 μg/ml of m‐NPe increased proportional to the concentration of m‐NPe, underscoring the potential role of *shlA* in virulence factor production^30^. Additional details of the *T. molitor* experiment results are provided in Figure S12. Based on the in vitro and in vivo results, we believe that the pure *shlA* antagonist m‐NPe inhibits prodigiosin and hemolysin synthesis by downregulating the expression of genes (e.g., *pigA*, *pigC*, and *shlA*) involved in virulence factor production^30^. In addition, we performed cytotoxic tests on mouse embryonic fibroblasts (MEFs) and mouse erythrocytes to evaluate the safety profile of the investigated derivatives, affirming their lower hazard to cells, with all inhibition rates remaining less than 5%. Further details are provided in Figure S13.

## DISCUSSION

Based on previous studies on QSIs^9^, ^10^, ^11^, ^12^, ^13^, we aimed to synthesize an effective QSI against *S. marcescens* by creating new β‐nitrostyrene compounds^35^. In this study, the rate of biofilm inhibition^36^ was measured at 1/2 and 1/4 MICs (Figure 3A). In *S. marcescens* NJ01, the 1/2 MIC concentration was detrimental as most of the β‐nitrostyrene derivatives inhibited the formation of biofilms. At 1/4 MICs, the rate of inhibition declined. Interestingly, the m‐NPe showed an exceptional effect at 1/4 MIC as it inhibited biofilm formation by 63%. Compared to m‐NPe, other β‐nitrostyrene derivatives exerted less promising biofilm‐inhibitory effects. However, a number of the compounds (8, 9, 11, 12, 25, and 31) were still effective (Table S2). Specifically, we aimed to synthesize β‐nitrostyrene derivatives to inhibit prodigiosin in the study, which is one of the vital virulent factors of *S. marcescens* ^37^. We evaluated the inhibition rates and QSI efficacy of our β‐nitrostyrene derivatives using prodigiosin, a QS molecule that may contribute to invasion, survival, and pathogenicity (Figure 3B). Interestingly, the prodigiosin assay revealed that m‐NPe could significantly block prodigiosin, resulting in a reduction of biofilm formation (Table S3). Given its significant involvement in suppressing prodigiosin synthesis, particularly at sub‐MICs, we propose that m‐NPe may be a promising candidate for QS inhibition against *S. marcescens*.

Furthermore, the SAR analysis showed that compounds with a 2‐nitroethenyl benzene backbone (part B) were generally more potent than those with a 2‐nitroethenyl heterocyclic core (Figure 2). Furthermore, in part C, a 4‐electron‐donating substituent group of the benzene ring for the hydrophobic alkyl chain of no more than C‐2 length is preferred. The structural information highlights the importance of β‐nitrostyrene and hydrophobic side chains. Upon assessing 33 β‐nitrostyrene derivatives for structural stability, we found that m‐NPe could be a significant and promising antibiofilm agent.

Interestingly, the suppression of QS‐associated gene expression validated m‐NPe's potent QSI activities (Figure 8). m‐NPe not only inhibited biofilm formation but also displayed the capability to decompose existing biofilms (Figures 4 and 5). To gain a better understanding of its antivirulence and antibiofilm potential, molecular docking and MD were performed on proteins implicated in the QS system (SmaR, RhlI, RhlR, LasR, and CviR) (Figure S8), and conformed the binding stability and interaction energy with QS target proteins (Figures 9 and S9). Finally, the in vivo toxicity of m‐NPe was assessed with *T. molitor* larvae and showed a safe effect (Figure S11).

Furthermore, the substantial QSI capabilities of m‐NPe were evaluated by assessing the suppression of major *S. marcescens* virulence components associated with QS, including prodigiosin, lipase, protease, and swarming motility (Figures 6 and 7). Notably, m‐NPe significantly suppressed the two virulence factors hemolysin and prodigiosin, resulting in downregulation of related genes. Moreover, m‐NPe could inhibit the production of lipase and extracellular protease of *S. marcescens* NJ01 and *S. marcescens* 4547, which are implicated in cytolytic activities and host infection^38^. Swarming, primarily orchestrated by flagella and governed by *flhD*, is evidently critical in pathogen colonization, particularly in facilitating initial attachment and biofilm formation^26^. On the one hand, genes *fimA* and *fimC* regulate fimbriae subunits, which play a key role in the adhesin attachment and colonization of *S. marcescens* ^26^. On the other hand, genes *bsmA* and *bsmB* can significantly enhance biofilm formation in *S. marcescens* ^39^. Our results clearly demonstrated that m‐NPe exposure causes downregulation of key genes (such as *fimA*, *bsmB*, *fimC*, *flhD*) (Figure 8), which facilitates the suppression of biofilm formation, attachment, and swarming motility.

Biofilms can be formed on both biotic and abiotic surfaces, and the bacterial population creates a complex network structure that is either attached to the surface or embedded in a scaffold by the extracellular matrix^40^. EPS, an essential component of biofilm^41^, is significantly suppressed after exposure to m‐NPe (50 μg/ml), with an inhibition rate of up to 52% (Figure 5E). Remarkably, m‐NPe (50 μg/ml) demonstrated an outstanding effect of biofilm decomposition by 84% in *S. marcescens* NJ01. m‐NPe also showed similar potential activity in other strains, such as *S. marcescens* 4547. In addition, the effects of m‐NPe on biofilm decomposition were further confirmed by SEM (Figure 5C) and CLSM (Figure 5D). All of these findings suggest that m‐NPe, at varying concentrations, possesses essential characteristics and potential to inhibit and destruct biofilms. The superimposition of protein and m‐NPe produced similar interactions, with the aromatic substituent methyl group precisely aligning with the alkyl chain position, as predicted. The successful interaction of m‐NPe with SmaR was verified by MST (Figure S10). This supports our claim that SmaR is the target protein for *S. marcescens*.

In summary, this study used a systematic strategy to design, synthesize, and analyze β‐nitrostyrene derivatives to create a new class of QSIs targeting *S. marcescens*. A ligand‐based design was adopted to explore the SAR of NPe derivatives. The β‐nitrostyrene scaffold displayed obvious QS activity with increased effectiveness. The comprehensive evaluation showed that m‐NPe is the most effective of 33 synthesized β‐nitrostyrene derivatives, suppressing biofilms by 80% and prodigiosin by 73% at the sub‐MIC level. When using m‐NPe against the reporter strain *C. violaceum* CV026, it showed inhibitory effects. Moreover, tested the mortality ratio of *T. molitor* larvae, m‐NPe showed minimal toxicity. Molecular docking and MD analysis were used to elucidate the potential mechanisms underlying the antivirulence and antibiofilm properties of m‐NPe. Furthermore, RT‐qPCR studies showed that m‐NPe reduced the expression of QS‐ and biofilm‐related genes by up to 2.32‐fold. These results significantly confirmed m‐NPe's potential as an effective QSI and antibiofilm agent against antimicrobial‐resistant *S. marcescens*, with implications for enhancing medication sensitivity and clinical therapeutic administration, and provided m‐NPe as a promising candidate for future development of new medicines against microbial infections.

## MATERIALS AND METHODS

### Synthesis of β‐nitrostyrene derivatives

The β‐nitrostyrene derivatives used in this study were designed and synthesized as compounds targeting QS for antibiofilm drug development (detailed information is provided in the Supporting Information section).

### Bacterial strains

The bacterial strains used in this study included the reporter strain *C. violaceum* CV026^31^, the plant pathogen *S. marcescens* NJ01, and the human pathogen *S. marcescens* 4547. *C. violaceum* CV026 was sourced from the Guangdong Provincial Center for Microbial Strains in Guangzhou, China. The *S. marcescens* NJ01 strains (GenBank accession No. MK092719) were kindly provided by W. Wang (Nanjing Agricultural University, Nanjing, China), and the *S. marcescens* 4547 strain was a gift from H. Huang (Haikou Municipal People's Hospital, Haikou, China). Unless otherwise stated, all strains were grown at 28°C in Luria Bertani (LB) broth (pH 7.0) medium.

### QS‐inhibitory screening of the β‐nitrostyrene derivatives

To investigate the QS‐inhibitory activities of the β‐nitrostyrene derivatives, the purity of all of the target compounds was determined to be greater than 95% (for more details, see the Supporting Information section). We initially evaluated the QS‐inhibitory activities of the β‐nitrostyrene derivatives against *S. marcescens* as described previously^42^. For the screening of QSIs^38^, *C. violaceum* CV026 was utilized as the reporter strain and the pathogen *S. marcescens* was used as the test strain.

### Determination of MICs and growth curves of the β‐nitrostyrene derivatives

The determination of MICs and growth curves of the β‐nitrostyrene derivatives has been carried out as described previously^43^. For details of the procedure, please refer to the Supporting Information section.

### Determination of AHL levels

The potential anti‐QS capability of the compound m‐NPe was evaluated by measuring the levels of secreted C4‐HSL and C6‐HSL^33^, ^34^ by *S. marcescens* NJ01. In brief, 0.1% overnight cultures of *S. marcescens* NJ01 were inoculated into 50 ml of LB, either with or without varying concentrations of compound m‐NPe (12.5, 25, and 50 μg/ml), and were then cultured at 28°C for 24 h. The negative control consisted of an equal volume of DMSO. Cells were extracted after culture by centrifugation at 4°C for 15 min. Three extractions of the supernatant were performed using acidified ethyl acetate (1:1, v/v). After that, the solvent was evaporated at decreased pressure and the residue was dissolved in methanol. AHLs were quantified using LC‐MS/MS^12^. Peaks corresponding to C4‐HSL and C6‐HSL were found using their MS/MS fragmentations and the retention duration of AHL standards. The ion *m*/*z* 102 was chosen for measurement because of its selectivity and high signal‐to‐noise ratio. Peak area calculations were performed on the extracted ion chromatograms, and the findings were standardized to the DMSO control to allow for relative quantification.

### Biofilm formation inhibition assay

Biofilms were cultured on 24‐well plates in LB broth with or without m‐NPe^15^. Following 24 h of static incubation, the cell cultures and planktonic cells were discarded, and the sessile cells were treated with 0.05% crystal violet and washed with distilled water. Biofilm biomass was determined at OD_570_ after dissolving with 95% ethanol.

Biofilms of *S. marcescens* NJ01 were cultivated in 24‐well plates with circular glass coverslips, as mentioned above. After cultivation, coverslips were washed with phosphate‐buffered saline (PBS), fixed with 2.5% glutaraldehyde, and dehydrated with ethanol. Samples were then freeze‐dried, gold‐coated, and detected with SEM (JSM6360; JEOL). Alternatively, the samples were also stained with 0.1% (w/v) acridine orange and imaged using CLSM (LEICA TCS SP8).

### Biofilm destruction assay

Biofilm was grown in LB broth on 24‐well plates overnight at 28°C^44^. The resulting biofilm was then rinsed in PBS before being transferred to LB broth and m‐NPe. After another overnight growth, the biofilm was rinsed with PBS, fixed with methanol, colored with crystal violet, dissolved in ethanol, and then measured using OD_570_.

As above stated, *S. marcescens* biofilms were grown in LB broth on 24‐well plates using a circular slide for microscopy analysis. Then, following fixation and dehydration, several biofilms were identified using SEM. The remaining biofilms were treated with acridine orange (AO) and ethidium bromide (EB), AO/EB (1:1, v/v), and identified by CLSM.

### Virulence factor assay

The virulence factor assay was carried out as described previously^5^, ^14^, ^23^, ^26^, ^27^, ^45^, ^46^, ^47^, ^48^, ^49^, ^50^. For details of the procedure, please refer to the Supporting Information section.

### RT‐qPCR

Briefly, 40 μl of *S. marcescens* of 24 h culture was added to 4 ml of fresh LB broth supplemented with DMSO or 50 μg/ml of m‐NPe. Overnight, cells were harvested by centrifugation. Total RNA was isolated from the *S. marcescens* pellet cells with an RNA extraction kit (Sangon Biotech). Isolated RNA was reverse‐transcribed into cDNA using the RT6 cDNA synthesis kit (Sangon Biotech). The RT‐qPCR reactions were accomplished using the LineGene 9600 Plus (Sangon Biotech). The *rplU* gene was used as the internal control^30^. The primers are shown in Table S4.

### Molecular docking and MD analysis

Molecular docking and MD analysis were carried out as described previously^8^, ^31^, ^33^, ^44^, ^51^, ^52^, ^53^, ^54^, ^55^, ^56^. For details of the procedure, please refer to the Supporting Information section.

### Mortality experiment of *T. molitor* larvae

For details of the procedure^57^, please refer to the Supporting Information section.

### Statistical analysis

Data are expressed as means ± standard deviation (SD) after each experiment was repeated at least three times. Graphs were created using GraphPad Prism (GraphPad Software). To compare differences across groups, a one‐way analysis of variance (ANOVA) was done using SPSS 18.0 software (SPSS, Inc.), followed by the Tukey–Kramer test. A *p* ≤ 0.05 was considered statistically significant.

## AUTHOR CONTRIBUTIONS

**Jiang Wang**: Conceptualization (lead); data curation (lead); formal analysis (equal); methodology (equal); project administration (equal); resources (lead); software (equal); supervision (equal); writing—original draft (equal); writing—review and editing (equal). **Jingyi Yang**: Conceptualization (lead); data curation (lead); resources (equal). **Pradeepraj Durairaj**: Conceptualization (equal); data curation (equal); resources (equal); software (equal); writing—original draft (lead); writing—review and editing (lead). **Wei Wang**: Conceptualization (equal); resources (equal). **Dongyan Wei**: Conceptualization (equal); resources (equal). **Shi Tang**: Conceptualization (equal); data curation (equal); resources (equal); software (equal); funding acquisition (lead). **Haiqing Liu**: Conceptualization (equal); resources (equal). **Dayong Wang**: Conceptualization (equal); data curation (equal); resources (equal); software (equal); funding acquisition (lead); writing—review and editing (lead). **Ai‐Qun Jia**: Data curation (equal); formal analysis (equal); funding acquisition (lead); project administration (lead); writing—original draft (lead); writing—review and editing (lead).

## ETHICS STATEMENT

This study did not involve human subjects and animals.

## CONFLICT OF INTERESTS

The authors declare no conflicts of interests.

## Supporting information

Supporting information.

## Data Availability

The protein structural databases used in this study were obtained from the Protein Data Bank (PDB) at https://www.rcsb.org. The CCDC 2179910 contains the supplemental crystallographic data. The data can be accessed at www.ccdc.cam.ac.uk/data_request/cif.
