# Supplementary material for: Discovery of β‐nitrostyrene derivatives as potential quorum sensing inhibitors for biofilm inhibition and antivirulence factor therapeutics against Serratia marcescens
Source: mLife. 2024 Sep 6;3(3):445–58. doi: 10.1002/mlf2.12135 (PMC11442132; doi:10.1002/mlf2.12135)
Supplement: Supplementary file 1 — Supporting information. [file MLF2-3-445-s001.pdf]

Supplementary Information

Discovery of *β*-nitrostyrene derivatives as potential quorum sensing inhibitors for biofilm inhibition and anti-virulence factor therapeutics against *Serratia marcescens*

Jiang Wang [a, b, d †], Jing-Yi Yang [c, d †], Pradeepraj Durairaj [b, e], Wei Wang [d], Dong-Yan Wei [d], Shi Tang [d, *], Hai-Qing Liu [d], Dayong Wang [d, *], Ai-Qun Jia [a, d *]

[a] Hainan Affiliated Hospital of Hainan Medical University, Hainan General Hospital, Haikou 570311, China.

[b] Center for Translational Research, Shenzhen Bay Laboratory, Shenzhen 518132, China.

[c] Hainan Branch, Shanghai Children's Medical Center, School of Medicine, Shanghai Jiao Tong University, Sanya 572022, China.

[d] Key Laboratory of Tropical Biological Resources of Ministry of Education, School of Pharmaceutical Sciences, Hainan University, Haikou 570228, China.

[e] National High Magnetic Field Laboratory, FAMU-FSU College of Engineering, Florida State University, Tallahassee, Florida 32310, USA.

[†] These authors contributed equally to this study.

* To whom the correspondence should be addressed:

Ai-Qun Jia, Hainan Affiliated Hospital of Hainan Medical University, Hainan General Hospital, Haikou 570311, China. Email: aqjia@hainmc.edu.cn;

Dayong Wang, Key Laboratory of Tropical Biological Resources of Ministry of Education, School of Pharmaceutical Sciences, Hainan University, Haikou 570228, China. Email: wangdy@hainanu.edu.cn;

Table of Contents

1 Synthesis of compounds S3
2. Screening of compounds inhibiting quorum sensing S12
4. Inhibition of biofilm formation S16
5. Prodigiosin inhibition rates S18
6. Primers for RT-qPCR S22
7. Molecular docking S23
8. Molecular dynamics analysis of binding stability of m-NPe S27
9. Cytotoxicity of m-NPe S29
10. NMR spectra of compounds S43
11. X-ray crystallographic data S60

1 Synthesis of compounds

1.1 General information General information

^1H and ^{13}C Nuclear Magnetic Resonance (NMR) spectra were recorded on Bruker Avance 400 Ultrashield NMR spectrometers. ^{19}F NMR spectra were recorded on a Varian 400 instrument spectrometer. Chemical shifts (δ) were measured using tetramethylsilane (TMS) as a standard. High-resolution mass spectrometry (HRMS) data were obtained on an FTICR-MS instrument (Ionspec 7.0 T). The melting points were determined on an SGW X-4 melting point apparatus with microscope. Reactions were monitored by thin layer chromatography (TLC). Flash column chromatography was performed using silica gels (200-300 mesh).

1.2 Synthesis of β-nitrostyrene derivatives

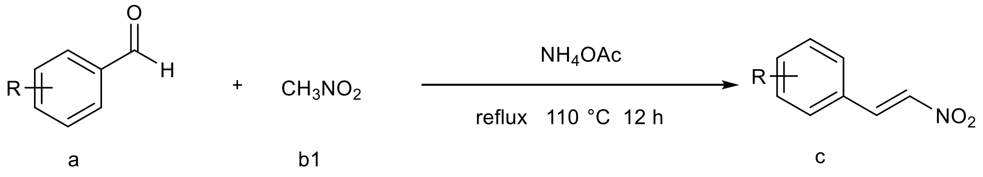

Scheme S1

Synthetic method for compound c [1]: Benzaldehyde (3.00 mmol) was added to a stirred solution of ammonium acetate (58 mg, 0.75 mmol) in dry nitromethane b1 (5 mL) at 110 ℃. The mixture was heated and refluxed for 5 h, poured into water and extracted with ethyl acetate (3 × 50 mL). The extract was washed with brine, dried by Na_2SO_4, filtered and evaporated under reduced pressure. The residue was crystallized in ethanol to yield the final product (c). Alternatively, the extract was concentrated under vacuum, and purified by flash chromatography on SiO_2 (PE/EA = 20:1) to yield the product (c), which is a light-yellow solid.

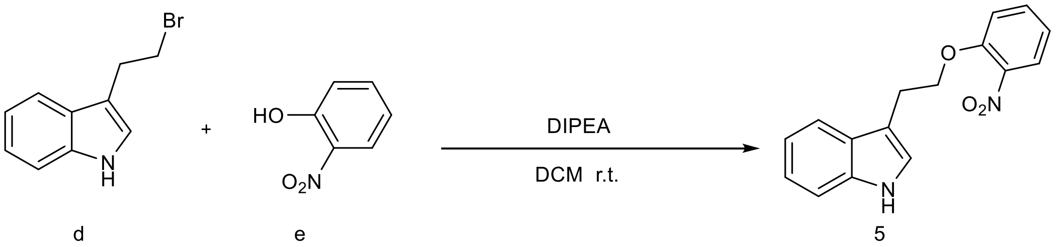

Scheme S2

Synthetic method for compound 5: 417 mg of 2-Nitrophenol (d) was added to 5 mL of dichloromethane, then 695 μL of *N,N*-diisopropylethylamine was dropped into the solution. After stirring for 5 min, 448 mg of 3-(2-bromoethyl)-1*H*-indole (e) was added. The mixture was stirred for 8 h at room temperature, poured into water and extracted with ethyl acetate (3 × 50 mL). The extract was washed with brine, dried by Na_2SO_4, filtered and evaporated under reduced pressure. The residue was concentrated under vacuum, and purified by flash chromatography on SiO_2 (PE/EA = 20:1). The final product (5) was green solid.

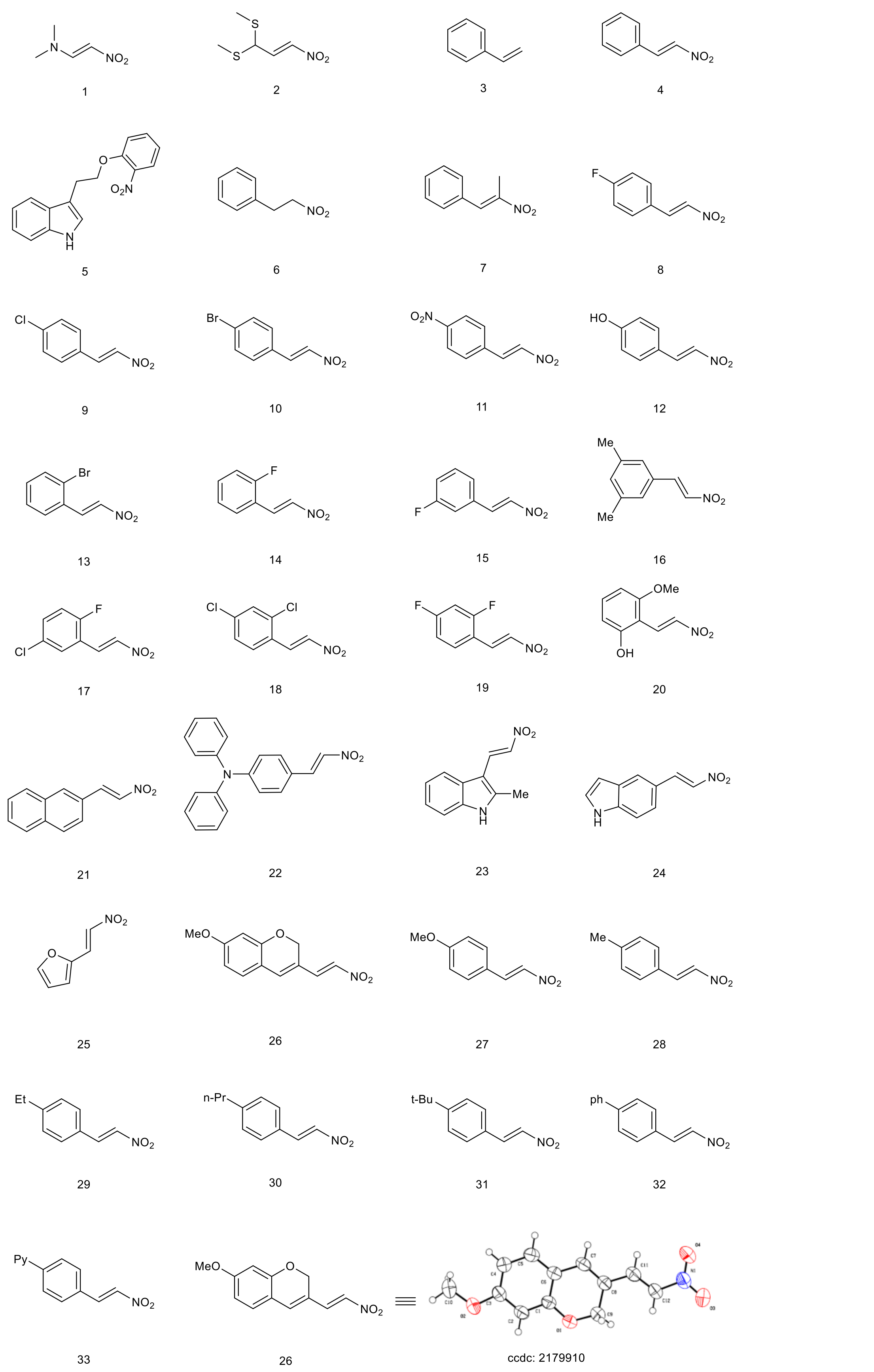

Scheme S3. Chemical structures of all *β*-nitrostyrene derivatives used for QS inhibitory testing in *S. marcescens.*

1.3 Characterization data

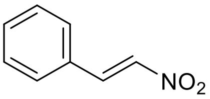

(E)-(2-Nitrovinyl)benzene (4), colorless solid (0.366 g, 82%); mp 56 °C. ^{1}H NMR (400 MHz, $CDCl_3$) δ 8.01 (d, *J* = 13.7 Hz, 1H), 7.64 – 7.52 (m, 3H), 7.51 – 7.42 (m, 3H). MS (ESI) m/z: calculated for $C_8H_7NO_2$ $[M + H]^+$, 150.0555; found, 150.06;

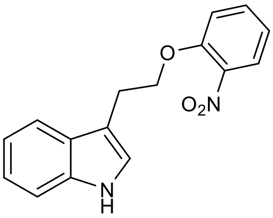

3-(2-(2-Nitrophenoxy)ethyl)-1H-indole (5), green solid (0.316 g, 56%); mp 150 °C. ^{1}H NMR (400 MHz, $CDCl_3$) δ 8.08 (s, 1H), 7.82 (dd, *J* = 8.1, 1.7 Hz, 1H), 7.63 (d, *J* = 8.2 Hz, 1H), 7.53 – 7.43 (m, 1H), 7.39 – 7.34 (m, 1H), 7.25 – 7.16 (m, 2H), 7.18 – 7.10 (m, 1H), 7.07 – 6.93 (m, 2H), 4.34 (t, *J* = 6.8 Hz, 2H), 3.32 (t, *J* = 6.8 Hz, 2H). ^{13}C NMR (100 MHz, $CDCl_3$) δ 152.5, 140.1, 136.2, 134.2, 127.4, 125.7, 123.2, 122.2, 120.3, 119.6, 118.6, 114.5, 111.7, 111.4, 69.8, 25.2. HRMS (ESI) m/z: calculated for $C_{16}H_{14}N_2O_3$ $[M + H]^+$, 283.1083; found, 283.1080;

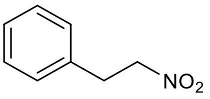

(2-Nitroethyl)benzene (6), colorless solid (0.508 g, 75%); mp 58 °C. ^{1}H NMR (400 MHz, $CDCl_3$) δ 7.34 – 7.24 (m, 3H), 7.22 – 7.12 (m, 2H), 4.57 (t, *J* = 7.4 Hz, 2H), 3.28 (t, *J* = 7.4 Hz, 2H). MS (ESI) m/z: calculated for $C_8H_9NO_2$ $[M + H]^+$, 152.0712; found, 152.07;

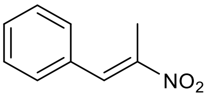

(E)-(2-Nitroprop-1-en-1-yl)benzene (7), colorless solid (0.342 g, 70%); mp 62 °C. ^{1}H NMR (400 MHz, $CDCl_3$) δ 8.04 (s, 1H), 7.84 – 7.01 (m, 5H), 2.41 (d, *J* = 1.4 Hz, 3H). MS (ESI) m/z: calculated for $C_9H_9NO_2$ $[M + H]^+$, 164.0712; found, 164.07;

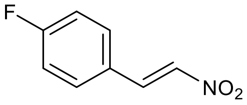

(E)-1-Fluoro-4-(2-nitrovinyl)benzene (8), yellow solid (0.376 g, 75%); mp 100 °C. ^{1}H NMR (400 MHz, $CDCl_3$) δ 7.99 (d, *J* = 13.7 Hz, 1H), 7.64 – 7.50 (m, 3H), 7.16 (t, *J* = 8.5 Hz, 2H). MS (ESI) m/z: calculated for

$C_8H_6FNO_2$ [M + H]$^+$, 168.0461; found, 168.04;

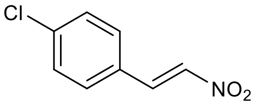
(E)-1-Chloro-4-(2-nitrovinyl)benzene (9), green solid (0.450 g, 82%); mp 115 °C. ^{1}H NMR (400 MHz, $CDCl_3$) δ 7.96 (d, *J* = 13.7 Hz, 1H), 7.56 (d, *J* = 13.7 Hz, 1H), 7.51 – 7.47 (m, 2H), 7.46 – 7.40 (m, 2H). MS (ESI) m/z: calculated for $C_8H_6ClNO_2$ [M + H]$^+$, 184.0165; found, 184.02;

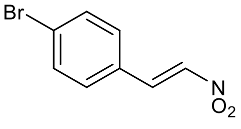
(E)-1-Bromo-4-(2-nitrovinyl)benzene (10), yellow solid (0.513 g, 75%); mp 150 °C. ^{1}H NMR (400 MHz, $CDCl_3$) δ 7.94 (d, *J* = 13.7 Hz, 1H), 7.66 – 7.54 (m, 3H), 7.42 (d, *J* = 8.2 Hz, 2H). MS (ESI) m/z: calculated for $C_8H_6BrNO_2$ [M + H]$^+$, 227.9660; found, 227.97;

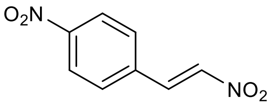
(E)-1-Nitro-4-(2-nitrovinyl)benzene (11), yellow solid (0.466 g, 80%); mp 200 °C. ^{1}H NMR (400 MHz, $CDCl_3$) δ 8.32 (d, *J* = 8.7 Hz, 2H), 8.05 (d, *J* = 13.7 Hz, 1H), 7.75 (d, *J* = 8.7 Hz, 2H), 7.66 (d, *J* = 13.8 Hz, 1H). MS (ESI) m/z: calculated for $C_8H_6N_2O_4$ [M + H]$^+$, 195.0406; found, 195.04;

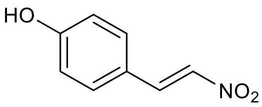
(E)-4-(2-Nitrovinyl)phenol (12), yellow solid (0.371 g, 75%); mp 170 °C. ^{1}H NMR (400 MHz, MeOH-*d*$_4$) δ 7.97 (d, *J* = 13.5 Hz, 1H), 7.73 (d, *J* = 13.6 Hz, 1H), 7.56 – 7.46 (m, 2H), 6.98 – 6.79 (m, 2H). MS (ESI) m/z: calculated for $C_8H_7NO_3$ [M + H]$^+$, 166.0505; found, 166.05;

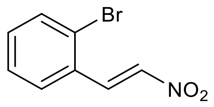
(E)-1-Bromo-2-(2-nitrovinyl)benzene (13), yellow solid (0.520 g, 76%); mp 87 °C. ^{1}H NMR (400 MHz, $CDCl_3$) δ 8.39 (d, *J* = 13.7 Hz, 1H), 7.69 (dd, *J* = 7.8, 1.6 Hz, 1H), 7.61 – 7.49 (m, 2H), 7.45 – 7.29 (m, 2H). MS (ESI) m/z: calculated for $C_8H_6BrNO_2$ [M + H]$^+$, 227.9660; found, 227.96;

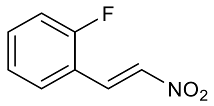
(E)-1-Fluoro-2-(2-nitrovinyl)benzene (14), yellow solid (0.366 g, 73%); mp 57 °C. ^{1}H NMR (400 MHz, $CDCl_3$) δ 8.05 (d, *J* = 13.8 Hz, 1H), 7.73 (d, *J* = 13.8 Hz, 1H), 7.56 – 7.43 (m, 2H), 7.33 – 7.12 (m, 2H). MS (ESI) m/z: calculated for $C_8H_6FNO_2$ [M + H]$^+$, 168.0461; found, 168.05;

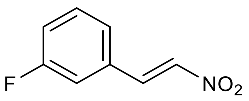 *(E)*-1-Fluoro-3-(2-nitrovinyl)benzene (15), yellow solid (0.508 g, 75%); mp 48 °C. ^{1}H NMR (400 MHz, $CDCl_3$) δ 7.97 (d, *J* = 13.7 Hz, 1H), 7.57 (d, *J* = 13.7 Hz, 1H), 7.45 (td, *J* = 8.0, 5.7 Hz, 1H), 7.35 (dt, *J* = 7.7, 1.3 Hz, 1H), 7.29 – 7.15 (m, 2H). MS (ESI) m/z: calculated for $C_8H_6FNO_2$ $[M + H]^+$, 168.0461; found, 168.05;

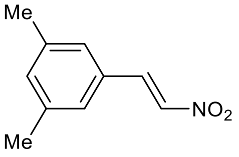 *(E)*-1,3-Dimethyl-5-(2-nitrovinyl)benzene (16), yellow solid (0.350 g, 66%); mp 110 °C. ^{1}H NMR (400 MHz, $CDCl_3$) δ 7.95 (d, *J* = 13.6 Hz, 1H), 7.57 (d, *J* = 13.7 Hz, 1H), 7.15 (d, *J* = 10.9 Hz, 3H), 2.35 (s, 6H). MS (ESI) m/z: calculated for $C_{10}H_{11}NO_2$ $[M + H]^+$, 178.0868; found, 178.08;

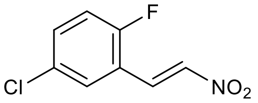 *(E)*-4-Chloro-1-fluoro-2-(2-nitrovinyl)benzene (17), yellow solid (0.452 g, 75%); mp 120 °C. ^{1}H NMR (400 MHz, $CDCl_3$) δ 8.01 (d, *J* = 13.7 Hz, 1H), 7.72 (d, *J* = 13.8 Hz, 1H), 7.49 (t, *J* = 8.2 Hz, 1H), 7.36 – 7.19 (m, 2H). MS (ESI) m/z: calculated for $C_8H_5ClFNO_2$ $[M + H]^+$, 202.0071; found, 202.01;

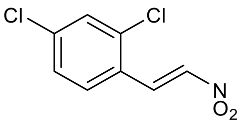 *(E)*-2,4-Dichloro-1-(2-nitrovinyl)benzene (18), green solid (0.458 g, 70%); mp 115 °C. ^{1}H NMR (400 MHz, $CDCl_3$) δ 8.33 (d, *J* = 13.7 Hz, 1H), 7.63 – 7.49 (m, 3H), 7.34 (dd, *J* = 8.5, 2.1 Hz, 1H). MS (ESI) m/z: calculated for $C_8H_5Cl_2NO_2$ $[M + H]^+$, 217.9776; found, 217.98;

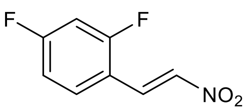 *(E)*-2,4-Difluoro-1-(2-nitrovinyl)benzene (19), yellow solid (0.416 g, 75%); mp 60 °C. ^{1}H NMR (400 MHz, $CDCl_3$) δ 8.34 (d, *J* = 13.7 Hz, 1H), 7.71 – 7.47 (m, 3H), 7.34 (dd, *J* = 8.5, 2.1 Hz, 1H). MS (ESI) m/z: calculated for $C_8H_5F_2NO_2$ $[M + H]^+$, 186.0367; found, 186.04;

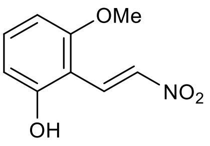 *(E)*-3-Methoxy-2-(2-nitrovinyl)phenol (20), yellow solid (0.398 g,

68%); mp 80 °C. ^{1}H NMR (400 MHz, $CDCl_3$) δ 10.65 (s, 1H), 9.83 (d, *J* = 0.7 Hz, 1H), 7.13 (dd, *J* = 9.0, 3.1 Hz, 1H), 6.99 (d, *J* = 3.1 Hz, 1H), 6.91 (d, *J* = 9.1 Hz, 1H), 3.80 (s, 3H). MS (ESI) m/z: calculated for $C_9H_9NO_4$ $[M + H]^+$, 168.0461; found, 168.04;

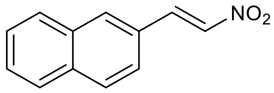

(E)-2-(2-Nitrovinyl)naphthalene (21), yellow solid (0.478 g, 80%); mp 122 °C. ^{1}H NMR (400 MHz, $CDCl_3$) δ 8.84 (d, *J* = 13.4 Hz, 1H), 8.14 (d, *J* = 8.8 Hz, 1H), 8.01 (d, *J* = 8.2 Hz, 1H), 7.92 (d, *J* = 6.7 Hz, 1H), 7.76 (d, *J* = 5.5 Hz, 1H), 7.68 – 7.49 (m, 4H). MS (ESI) m/z: calculated for $C_{12}H_9NO_2$ $[M + H]^+$, 200.0712; found, 200.07;

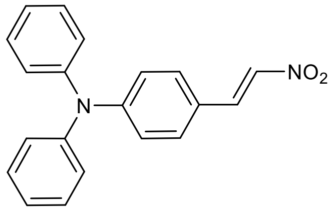

(E)-4-(2-Nitrovinyl)-N,N-diphenylaniline (22), yellow solid (0.569 g, 60%); mp 150 °C. ^{1}H NMR (400 MHz, $CDCl_3$) δ 7.94 (d, *J* = 13.5 Hz, 1H), 7.49 (d, *J* = 13.5 Hz, 1H), 7.38 – 7.29 (m, 6H), 7.18 – 7.09 (m, 6H), 6.98 (d, *J* = 8.8 Hz, 2H). MS (ESI) m/z: calculated for $C_{20}H_{16}N_2O_2$ $[M + H]^+$, 317.1290; found, 317.13;

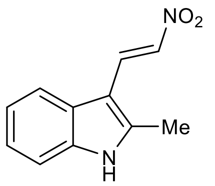

(E)-2-Methyl-3-(2-nitrovinyl)-1H-indole (23), yellow solid (0.455 g, 75%); mp 192 °C. ^{1}H NMR (400 MHz, $CDCl_3$) δ 8.81 (s, 1H), 8.34 (d, *J* = 13.3 Hz, 1H), 7.79 (d, *J* = 13.3 Hz, 1H), 7.73 – 7.64 (m, 1H), 7.43 – 7.35 (m, 1H), 7.32 – 7.26 (m, 2H), 2.64 (s, 3H). MS (ESI) m/z: calculated for $C_{11}H_{10}N_2O_2$ $[M + H]^+$, 203.0821; found, 203.08;

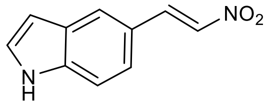

(E)-5-(2-Nitrovinyl)-1H-indole (24), yellow solid (0.451 g, 80%); mp 155 °C. ^{1}H NMR (400 MHz, $CDCl_3$) δ 8.55 (s, 1H), 8.17 (d, *J* = 13.5 Hz, 1H), 7.86 (s, 1H), 7.64 (d, *J* = 13.5 Hz, 1H), 7.48 – 7.36 (m, 2H), 7.32 – 7.26 (m, 1H), 6.66 – 6.60 (m, 1H). MS (ESI) m/z: calculated for $C_{10}H_8N_2O_2$ $[M + H]^+$, 189.0664; found, 189.06;

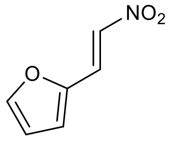

(E)-2-(2-Nitrovinyl)furan (25), colorless solid (0.313 g, 75%); mp 75 °C. ^{1}H NMR (400 MHz, $CDCl_3$) δ 7.78 (d, *J* = 13.2 Hz, 1H), 7.65 – 7.45 (m, 2H), 6.90 (d, *J* = 3.5 Hz, 1H), 6.58 (dd, *J* = 3.5, 1.8 Hz, 1H). MS (ESI) m/z: calculated for $C_6H_5NO_3$ $[M + H]^+$, 140.0348; found, 140.03;

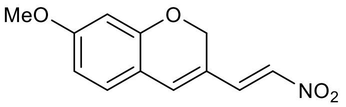

(E)-7-Methoxy-3-(2-nitrovinyl)-2H-chromene (26), yellow solid (0.524 g, 75%); mp 120 °C. ^{1}H NMR (400 MHz, $CDCl_3$) δ 7.68 (d, *J* = 13.5 Hz, 1H), 7.05 (d, *J* = 8.4 Hz, 1H), 6.95 (d, *J* = 13.4 Hz, 2H), 6.51 (dd, *J* = 8.5, 2.4 Hz, 1H), 6.41 (d, *J* = 2.4 Hz, 1H), 4.89 (s, 2H), 3.81 (s, 3H). ^{13}C NMR (100 MHz, CDCl3) δ 163.4, 156.6, 136.6, 136.5, 134.2, 129.9, 121.2, 114.7, 108.8, 101.5, 64.8, 55.6. HRMS (ESI) m/z: calculated for $C_{12}H_{11}NO_4$ $[M + H]^+$, 234.0766; found, 234.0763;

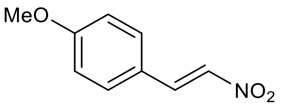

(E)-1-Methoxy-4-(2-nitrovinyl)benzene (27), colorless solid (0.424 g, 79%); mp 88 °C. ^{1}H NMR (400 MHz, $CDCl_3$) δ 7.95 (d, *J* = 13.6 Hz, 1H), 7.63 – 7.40 (m, 3H), 6.95 (d, *J* = 8.5 Hz, 2H), 3.86 (s, 3H). MS (ESI) m/z: calculated for $C_9H_9NO_3$ $[M + H]^+$, 180.0661; found, 180.06;

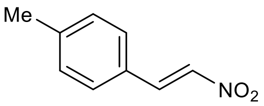

(E)-1-Methyl-4-(2-nitrovinyl)benzene (28), colorless solid (0.367 g, 75%); mp 103 °C. ^{1}H NMR (400 MHz, $CDCl_3$) δ 7.95 (d, *J* = 13.7 Hz, 1H), 7.54 (d, *J* = 13.6 Hz, 1H), 7.47 – 7.36 (m, 2H), 7.24 (d, *J* = 8.0 Hz, 2H), 2.39 (s, 3H).

^{13}C NMR (100 MHz, CDCl3) δ 143.2, 139.2, 136.3, 130.2, 129.3, 127.3, 21.7. MS (ESI) m/z: calculated for $C_9H_9NO_2$ $[M + H]^+$, 164.0712; found, 164.07;

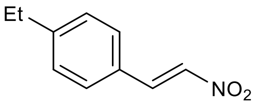

(E)-1-Ethyl-4-(2-nitrovinyl)benzene (29), colorless solid (0.414 g, 78%); mp 72 °C. ^{1}H NMR (400 MHz, $CDCl_3$) δ 7.99 (d, *J* = 13.6 Hz, 1H), 7.57 (d, *J* = 13.6 Hz, 1H), 7.47 (d, *J* = 8.2 Hz, 2H), 7.28 (d, *J* = 8.0 Hz, 2H), 2.70 (q, *J* = 7.6 Hz, 2H), 1.26 (t, *J* = 7.6 Hz, 3H). MS (ESI) m/z: calculated for $C_{10}H_{11}NO_2$ $[M + H]^+$, 178.0868; found, 178.09;

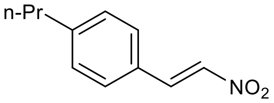

(E)-1-(2-Nitrovinyl)-4-propylbenzene (30), colorless solid (0.430 g, 75%); mp 90 °C. ^{1}H NMR (400 MHz, $CDCl_3$) δ 7.99 (d, *J* = 13.7 Hz, 1H), 7.57 (d, *J* = 13.6 Hz, 1H), 7.46 (d, *J* = 8.2 Hz, 2H), 7.26 (d, *J* = 8.3 Hz, 2H), 2.66 – 2.59 (m, 2H), 1.66 (m, 2H), 0.95 (t, *J* = 7.3 Hz, 3H). MS (ESI) m/z: calculated for $C_{11}H_{13}NO_2$ $[M + H]^+$, 192.1025; found, 192.10;

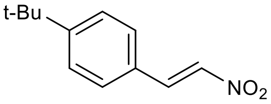

(E)-1-(*tert*-Butyl)-4-(2-nitrovinyl)benzene (31), yellow solid (0.430 g, 70%); mp 50 °C. ^{1}H NMR (400 MHz, $CDCl_3$) δ 8.01 – 7.90 (m, 1H), 7.57 (dd, *J* = 13.6, 1.5 Hz, 1H), 7.46 (d, *J* = 3.8 Hz, 4H), 1.36 – 1.27 (m, 9H). MS (ESI) m/z: calculated for $C_{12}H_{15}NO_2$ $[M + H]^+$, 206.1181; found, 206.12;

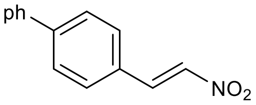

(E)-4-(2-Nitrovinyl)-1,1'-biphenyl (32), yellow solid (0.520 g, 77%); mp 127 °C. ^{1}H NMR (400 MHz, $CDCl_3$) δ 8.03 (d, *J* = 13.6 Hz, 1H), 7.67 (d, *J* = 8.4 Hz, 2H), 7.61 (tt, *J* = 5.8, 2.2 Hz, 5H), 7.47 (t, *J* = 7.4 Hz, 2H), 7.43 – 7.37 (m, 1H). MS (ESI) m/z: calculated for $C_{14}H_{11}NO_2$ $[M + H]^+$, 226.0868; found, 226.08;

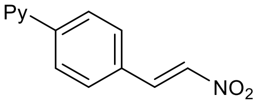

(E)-2-(4-(2-Nitrovinyl)phenyl)pyridine (33), yellow solid (0.340 g, 75%); mp 103 °C. ^{1}H NMR (400 MHz, $CDCl_3$) δ 8.89 (dd, *J* = 2.5, 0.9 Hz, 1H), 8.66 (dd, *J* = 4.8, 1.6 Hz, 1H), 8.06 (d, *J* = 13.7 Hz, 1H), 7.92 (m, 1H), 7.70 – 7.60 (m, 5H), 7.42 (m, 1H). MS (ESI) m/z: calculated for $C_{13}H_{10}N_2O_2$ $[M + H]^+$, 227.0821; found, 227.08;

Note: Compounds 1, 2, 3 was purchased from Sigma Aldrich in US.

References

[1] Xia, X. F., Shu, X. Z., Ji, K. G., Yang, Y. F., Shaukat, A., Liu, X. Y., & Liang, Y. M.. Platinum-Catalyzed Michael Addition and Cyclization of Tertiary Amines with Nitroolefins by Dehydrogenation of α, *β*-sp^3 C−H Bonds. *J. Org. Chem*. 2010, 75, 2893-2896.

2. Screening of compounds inhibiting quorum sensing

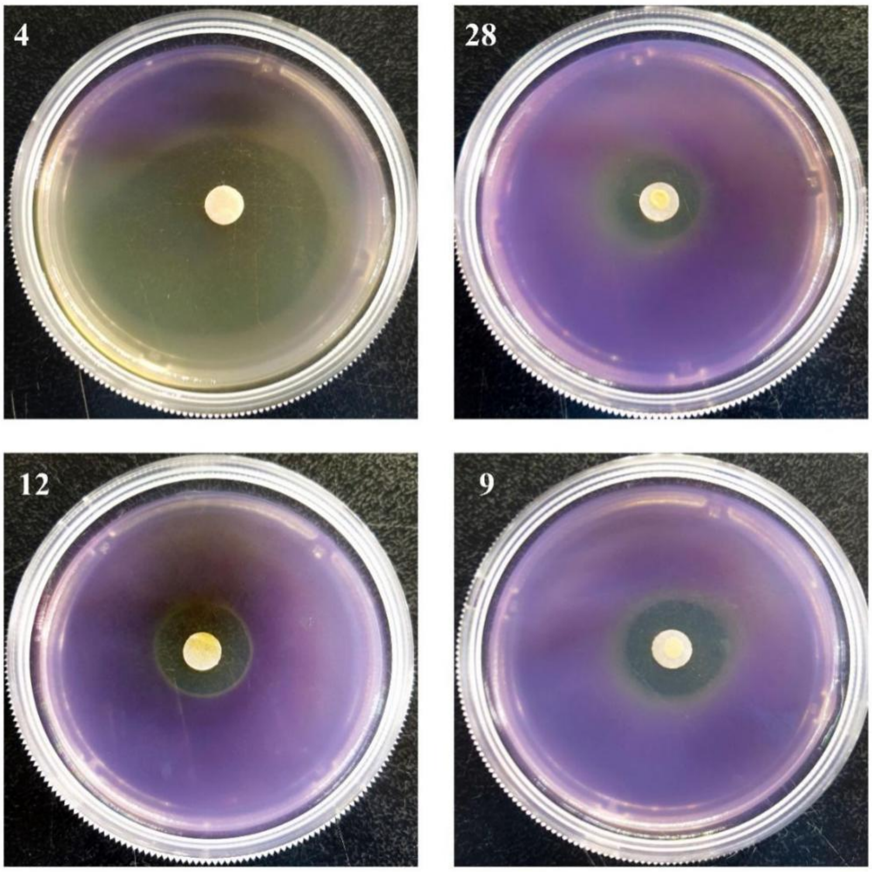

Figure S1. Inhibitory effects of compounds 4, 9, 12 and 28 on quorum sensing in *C. violaceum* CV026. The concentrations of the of compounds were 100 mg/mL. The strain *C. violaceum* CV026 was cultured at 28 °C on LB medium (pH 7.0). All assays were repeated at least three times.

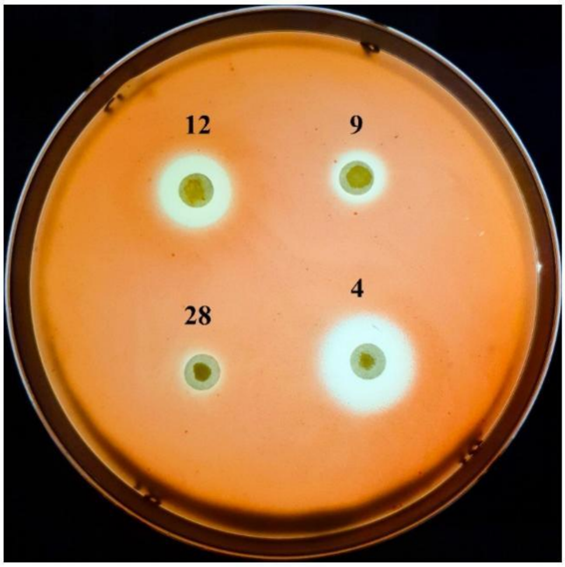

Figure S2. Inhibitory effects of compounds 4, 9, 12 and 28 on quorum sensing in *S. marcescens* NJ01. The concentrations of the compounds were 100 mg/mL. The strain *S. marcescens* NJ01 was cultured at 28 °C on LB medium (pH 7.0). All assays were repeated at least three times.

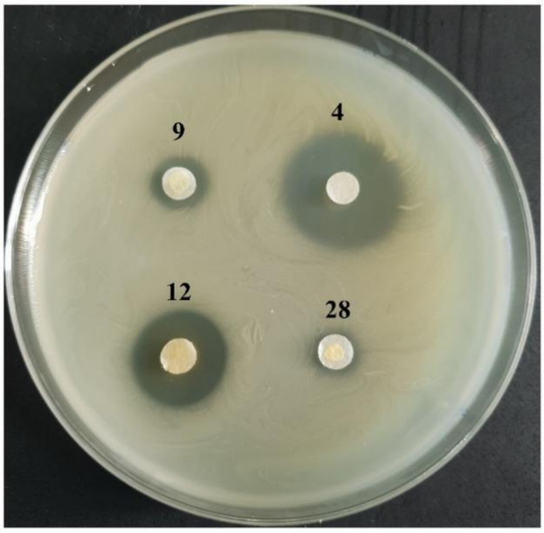

Figure S3. Inhibitory effects of compounds 4, 9, 12 and 28 on quorum sensing in *S. marcescens* 4547. The concentration was 100 mg/mL of compounds. The strain *S. marcescens* 4547 was cultured at 28 °C on LB medium (pH 7.0). All assays were repeated at least three times.

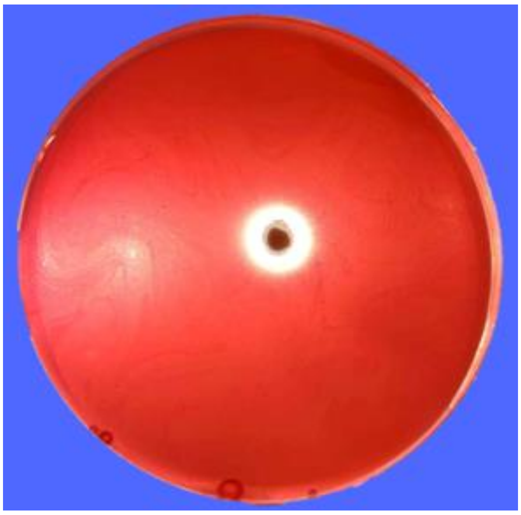

Figure S4 Inhibitory effects of the compound m-NPe (28) on quorum sensing in *S. marcescens* NJ01. The concentration of m-NPe was 100 mg/mL. The strain *S. marcescens* NJ01 was cultured at 28 °C on LB medium (pH 7.0). The assay was repeated three times.

3. Determination of MICs

Table S1. MICs of compounds 1–33

Compounds	MICs (μg/mL)	
	NJ01	4547
CPF	0.1	0.1
VAN	>96	>96
1	>96	>96
2	96	96
3	>96	>96
4	3	6
5	>96	>96
6	>96	>96
7	6	96
8	96	24
9	3	12
10	6	6
11	6	12
12	6	12
13	3	12
14	6	6
15	3	12
16	6	>96
17	3	24
18	6	24
19	3	24
20	96	>96
21	3	96
22	>96	>96
23	>96	>96
24	3	24
25	6	12
26	>96	>96

27	48	96
28	96	48
29	96	96
30	48	>96
31	6	>96
32	>96	>96
33	3	>96

Control: Ciprofloxacin (CPF), vanillic acid (VAN).

4. Inhibition of biofilm formation

Table S2. Inhibition rates of biofilm formation in *S. marcescens* NJ01 at 1/2 and 1/4 MICs of the compounds

Compounds	Biofilm inhibition	Biofilm inhibition
	1/4 MICs	1/2 MICs
1	0.41±0.041	0.50±0.004
2	0.57±0.033	0.67±0.016
3	0.41±0.029	0.55±0.121
4	0.58±0.042	0.77±0.014
5	0.37±0.012	0.40±0.060
6	0.34±0.071	0.42±0.017
7	0.41±0.077	0.52±0.062
8	0.56±0.012	0.71±0.008
9	0.55±0.072	0.74±0.032
10	0.41±0.033	0.48±0.084
11	0.56±0.037	0.69±0.064
12	0.56±0.055	0.72±0.024
13	0.44±0.009	0.46±0.062
14	0.54±0.027	0.68±0.030
15	0.50±0.063	0.79±0.029
16	0.11±0.092	0.29±0.096
17	0.31±0.053	0.59±0.038
18	0.47±0.038	0.65±0.020
19	0.30±0.051	0.58±0.024
20	0.06±0.033	0.25±0.041
21	0.30±0.076	0.60±0.044
22	0.10±0.081	0.17±0.085
23	0.36±0.068	0.52±0.039
24	0.50±0.081	0.56±0.005
25	0.56±0.058	0.61±0.080
26	0.17±0.077	0.44±0.077
27	0.44±0.087	0.56±0.091

28	0.63±0.050	0.80±0.020
29	0.38±0.080	0.72±0.034
30	0.31±0.050	0.50±0.087
31	0.50±0.044	0.62±0.029
32	0.35±0.056	0.38±0.032
33	0.43±0.051	0.57±0.021
Control	0.35±0.062	0.49±0.028

Control: Vanillic acid (VAN).

5. Prodigiosin inhibition rates

Table S3. Prodigiosin inhibition rates in *S. marcescens* NJ01 at 1/2 MIC of the compounds

Compounds	Prodigiosin inhibition
Control	0.24±0.069
1	0.30±0.045
2	0.42±0.036
3	0.25±0.132
4	0.54±0.109
5	0.15±0.048
6	0.30±0.028
7	0.55±0.034
8	0.71±0.109
9	0.56±0.023
10	0.48±0.031
11	0.31±0.086
12	0.65±0.068
13	0.33±0.037
14	0.54±0.079
15	0.61±0.034
16	0.53±0.091
17	0.46±0.071
18	0.42±0.023
19	0.32±0.027
20	0.40±0.056
21	0.21±0.049
22	0.31±0.187
23	0.50±0.015
24	0.59±0.081
25	0.65±0.045
26	0.52±0.014
27	0.65±0.036

28	0.73±0.064
29	0.65±0.003
30	0.38±0.018
31	0.40±0.100
32	0.62±0.017
33	0.52±0.024

Control: Vanillic acid (VAN).

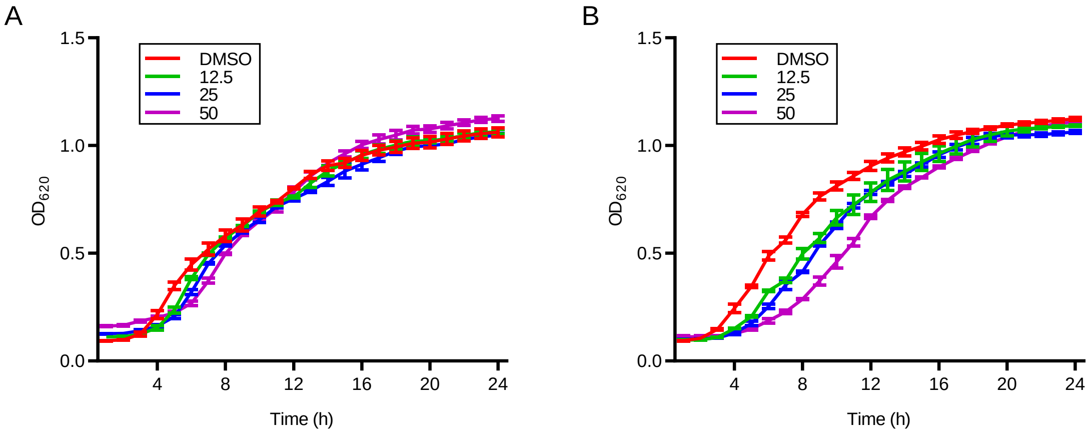

Figure S5. Growth profile of *S. marcescens* treated with m-NPe. (A) Growth profile of *S. marcescens* NJ01. (B) Growth profile of *S. marcescens* 4547. Growth was measured at different concentrations of m-NPe (12.5, 25, 50 μg/mL) for 24 h. DMSO was used as a negative control. Results are expressed as mean ± SD, n=3.

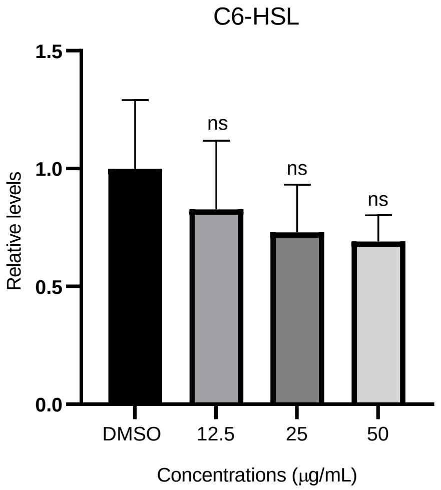

Figure S6. Effects of m-NPe on C6-HSL levels in *S. marcescens* tested by LC−MS/MS. Quantitative analysis of m-NPe (12.5, 25 and 50 μg/mL) on C6-HSL of *S. marcescens* using LC−MS/MS chromatograms. Results are expressed as mean ± SD, n=3. ns: not significant, analyzed by one-way ANOVA, followed by Tukey-Kramer post-hoc test for multiple comparisons.

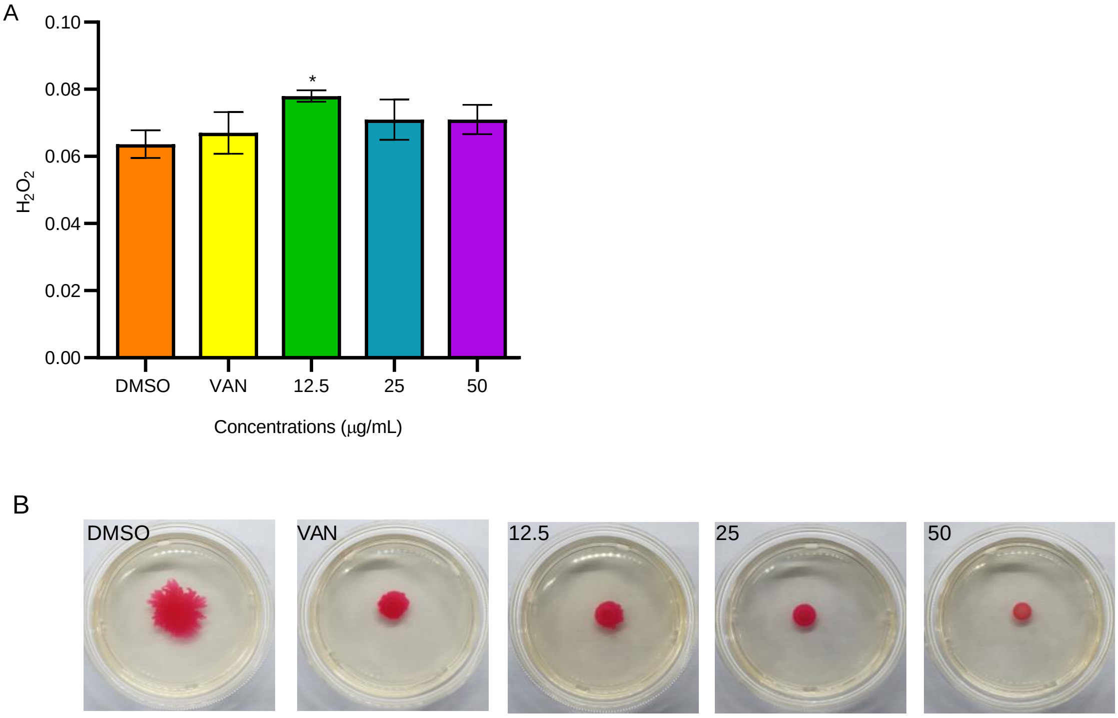

Figure S7. Inhibitory effects of m-NPe on virulence factor production in *S. marcescens* NJ01 (A) Levels of H_2O_2 produced in *S. marcescens* NJ01. (B) Effects of m-NPe on swarming motility. DMSO was used as a negative control. VAN: vanillic acid, the positive control. Results are expressed as mean ± SD, n=3. * $p < 0.05$, analyzed by one-way ANOVA, followed by Tukey-Kramer post-hoc test for multiple comparisons.

6. Primers for RT-qPCR

Table S4. Primers for RT-qPCR

genes	primer direction	sequence (5′ → 3′)
fimA	*fimA* forward	TTAGCCTGGAGAAATGTGAAGC
	fimA Reverse	GGCAGAGTAGAGCCGTTGTTAT
fimC	*fimC* Forward	AGCAGTTCAACACCTCCTTCAT
	fimC Reverse	CGGATATTTACCCGGCAGA
bsmB	*bsmB* Forward	CGGAAGTGACGCTGGAACACG
	bsmB Reverse	TGCTGCTGTTGATGGTGTAATCGG
pigA	*pigA* Forward	ATGGCTTTATGGGCGTGTC
	pigA Reverse	TGAAGGTCAGTTCGCTCCAC
pigC	*pigC* forward	TTCGTCACAAACCGCACTATT
	pigC reverse	CGTCTTTCACCGCCCATT
sodB	*sodB* forward	CTGCTGACCGTTGACGTGTGG
	sodB reverse	CGCTGCGAAGGTCCAGTTGAC
rplU	*rplU* forward	GCTTGGAAAAGCTGGACA
	rplU reverse	TACGGTGGTGTTTACGACGA
flhD	*flhD* forward	TGTCGGGATGGGGAATATGG
	flhD reverse	CGATAGCTCTTGCAGTAAATGG
shlA	*shlA* forward	GCGGCGATAACTATCAAAAT
	shlA reverse	ATTGCCAGGAGTAGAACCAG

7. Molecular docking

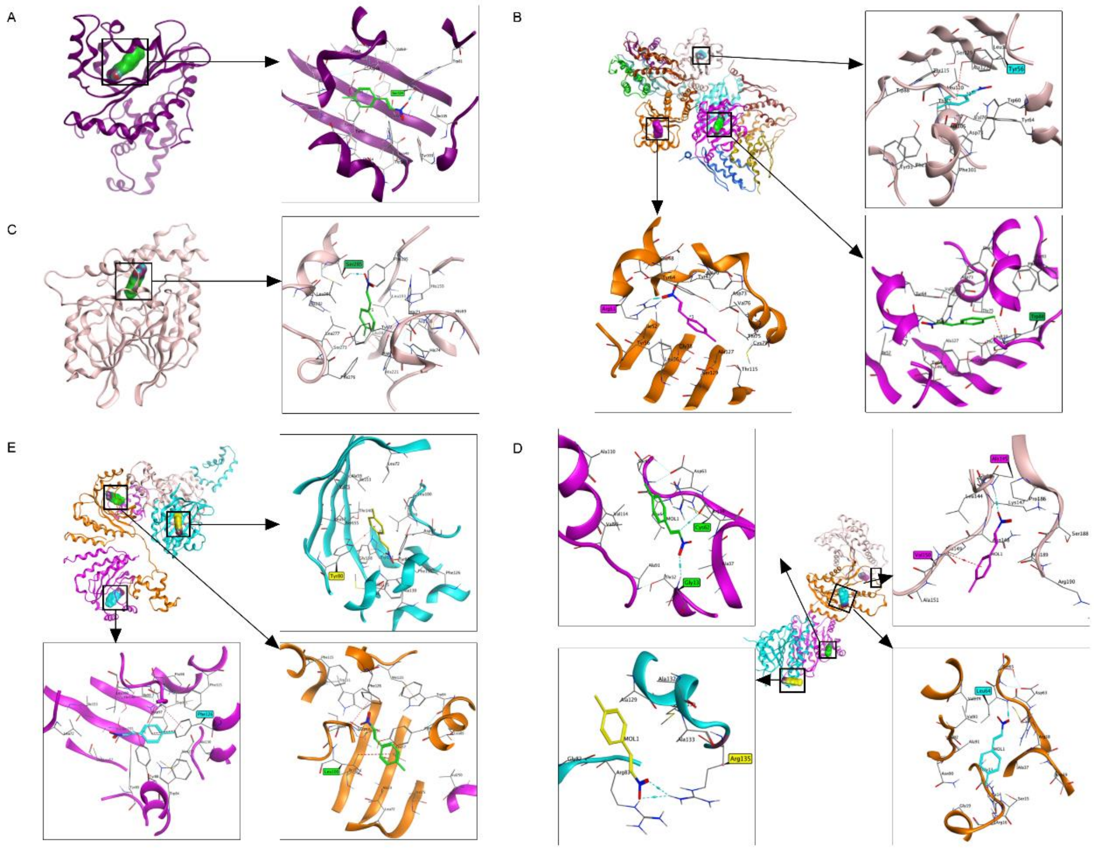

Figure S8. Molecular docking profiles. The 3D schematics of receptor-ligand interactions of different protein with m-NPe respectively were made using the Discovery Studio 4.0 program. (A) m-NPe with SmaR. (B) m-NPe with RhlI. (C) m-NPe with. RhlR. (D) m-NPe with LasR. (E) m-NPe with CviR.

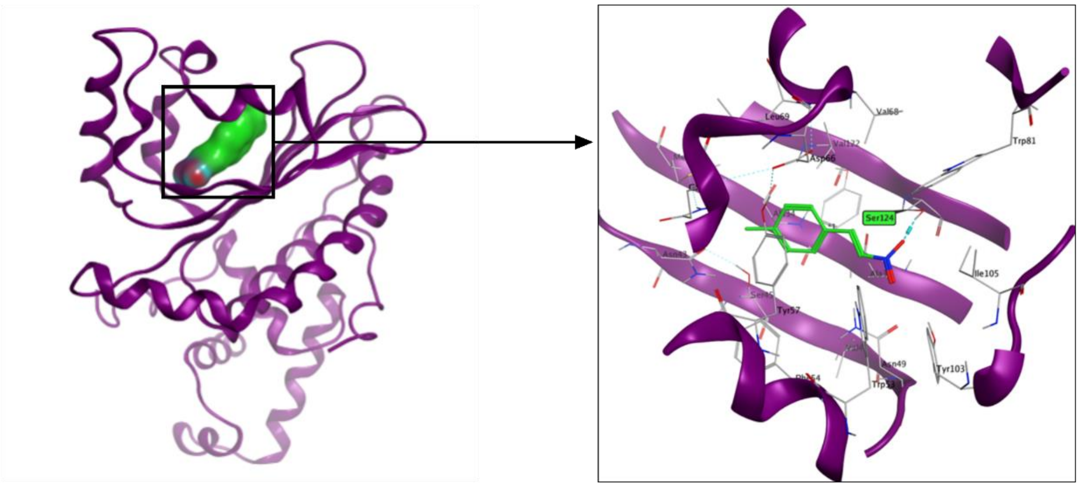

(A) m-NPe with SmaR.

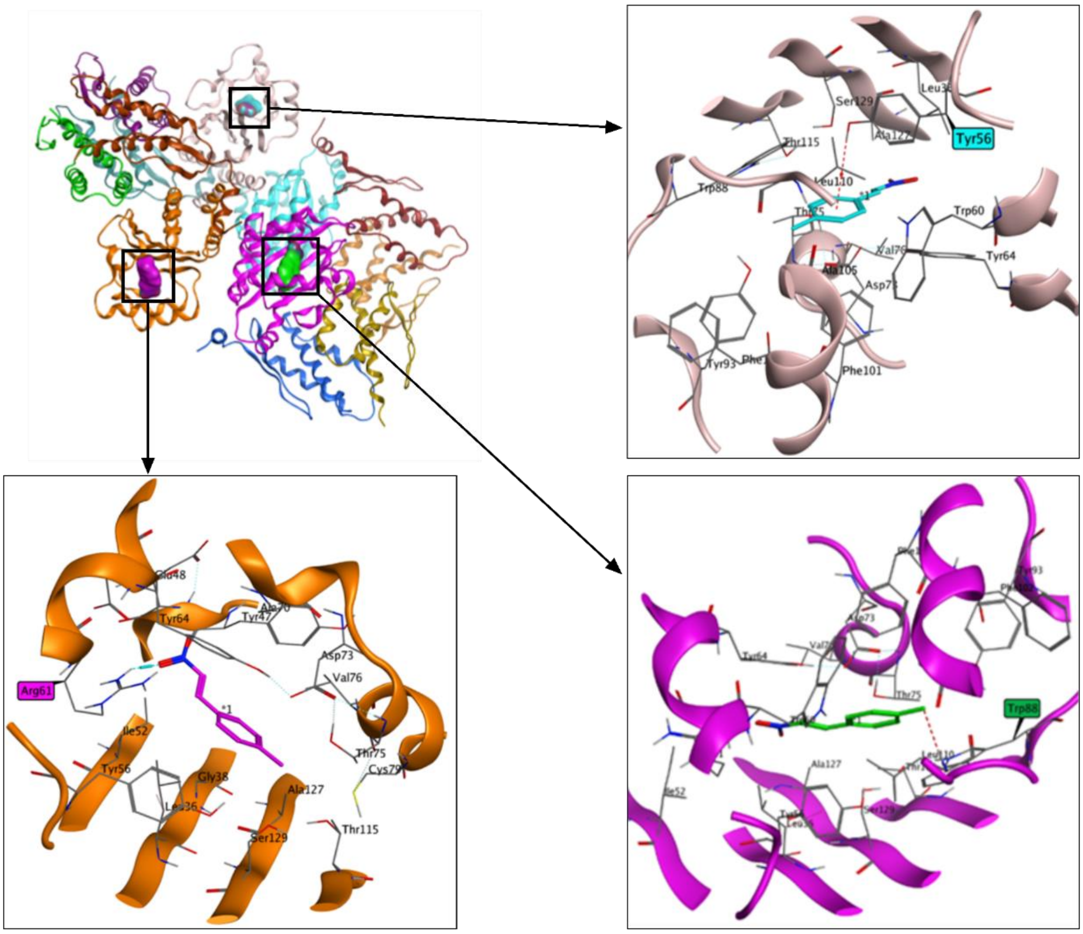

(B) m-NPe with RhlI.

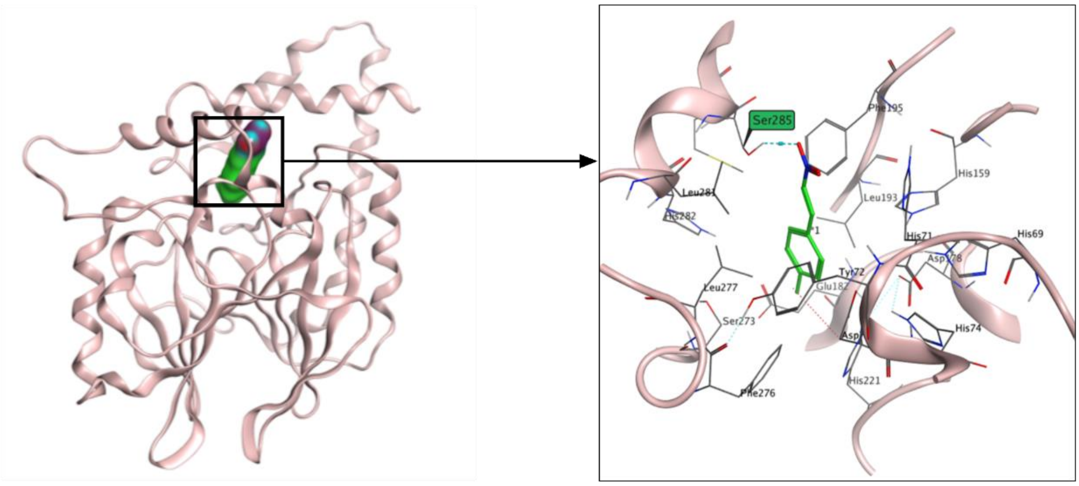

(C) m-NPe with. RhlR.

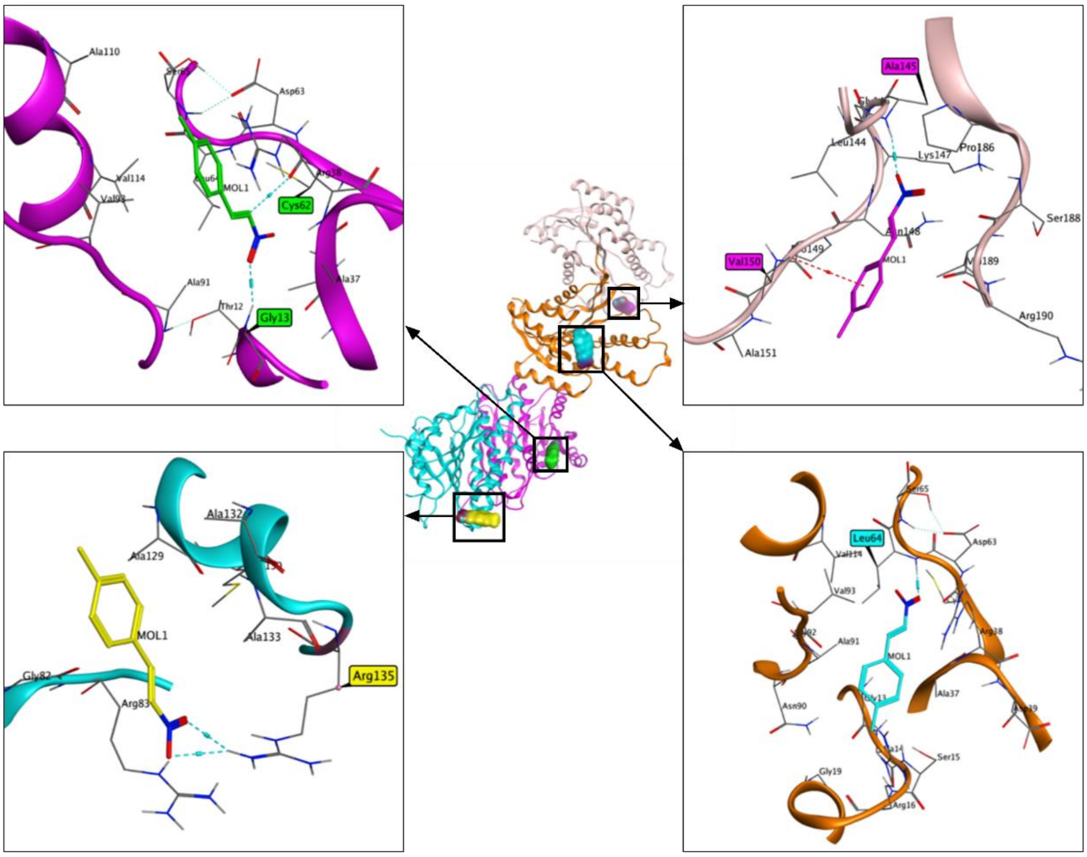

(D) m-NPe with LasR.

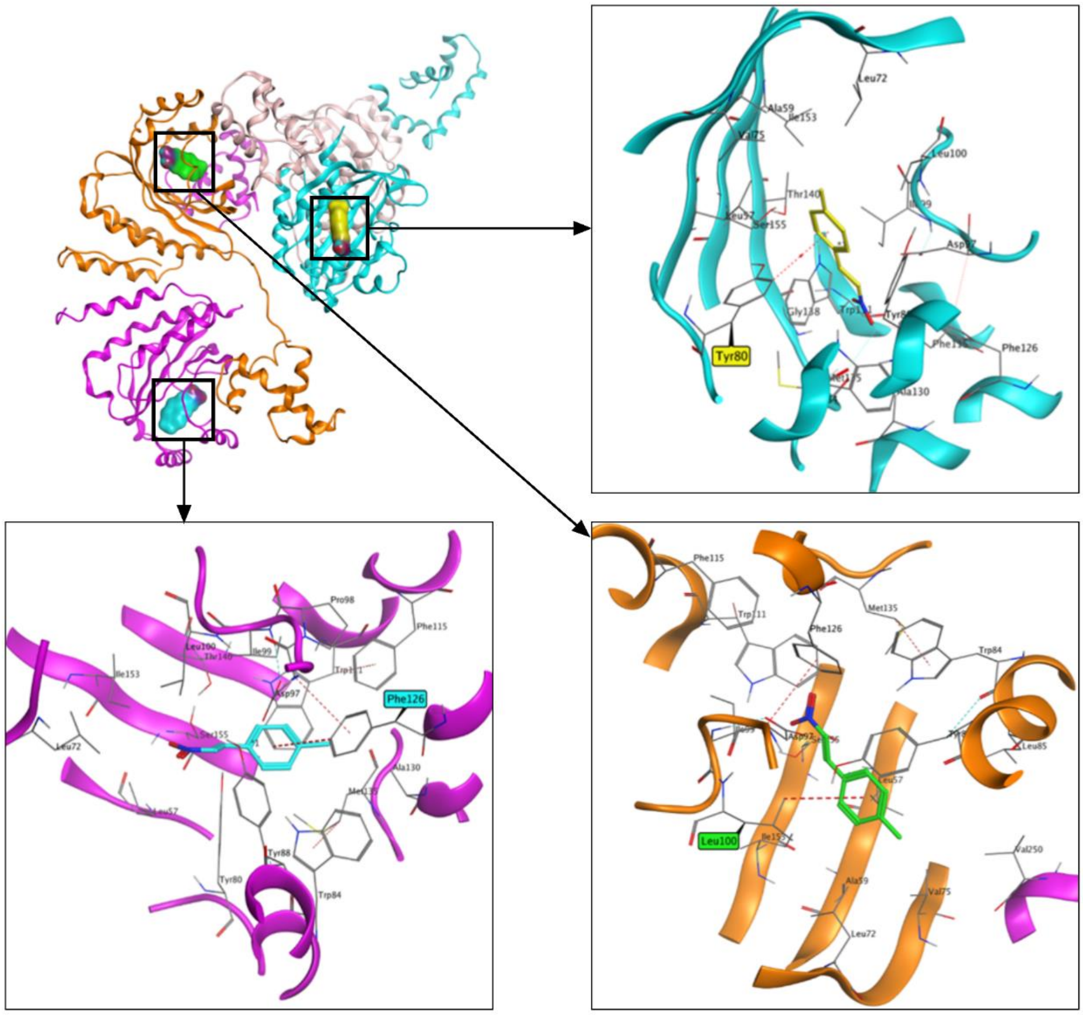

(E) m-NPe with CviR.

8. Molecular dynamics analysis of binding stability of m-NPe

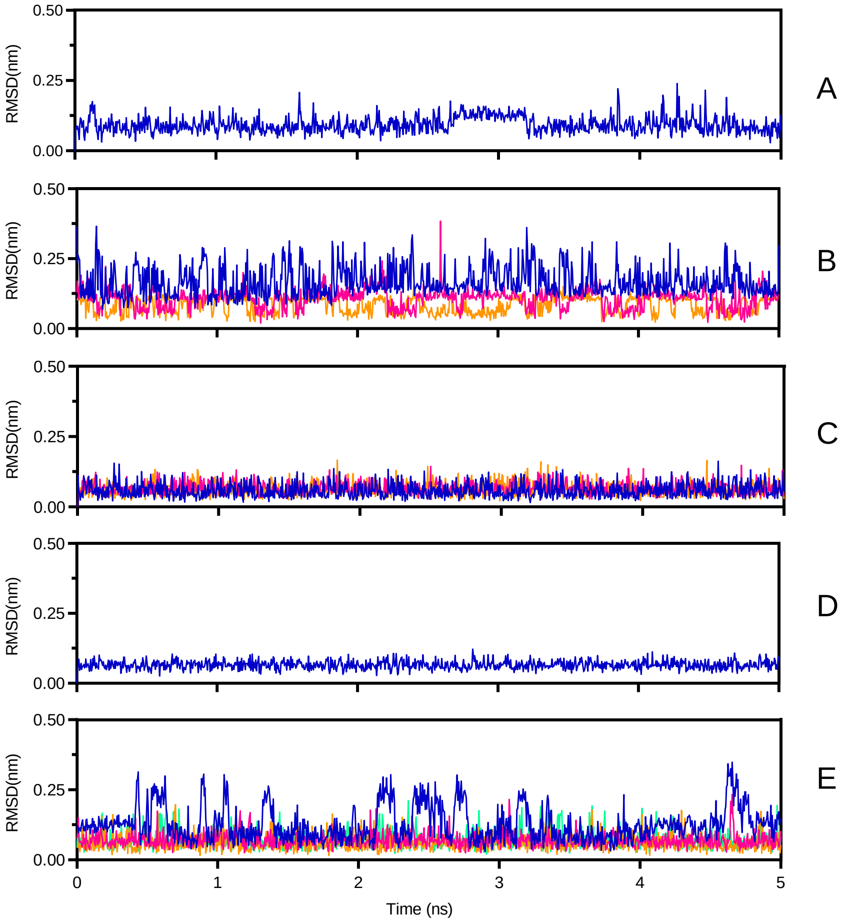

Figure S9. Profiles of RMSD over a 5-ns period. RMSD, root mean square deviation of the heavy atoms of m-NPe. RMSD profiles of m-NPe bound to SmaR (A), RhlI (B), RhlR (C), LasR (D) and CviR (E).

Table S5. SmaR protein sequence used in the study

Protein sequence	MSNFFFNNETINASFKEELERGLSKYNNIKYAYAIMNKR NPANFSIVSNRTEWFEFYIKNNYQFIDPVLITASHRITPF TWDKDLEIGSGLKLPKIFDMAKNYNIINGYTFVLHDHHH NLVVLSIMLDKHCDTDVEQQIDNNKAEIQMLLITMHGK MTALYQEMSTPADFEKMNQREFFSKRENEIIYWASLG KSYQEIALILGIKLTTVKYHIGNAVKKLGVTNAKHAIRLG VELQLIRPLLADAEG
GenBank ID	CAJ86500.1

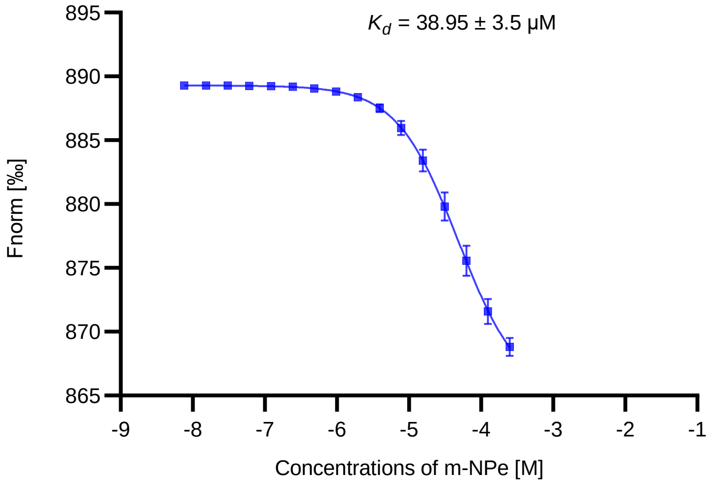

Figure S10. Microscale thermophoresis (MST) assay of the binding of m-NPe to SmaR. The curve was fitted using the standard K_d fit function. Results are expressed as mean ± SD, n=3.

9. Cytotoxicity of m-NPe

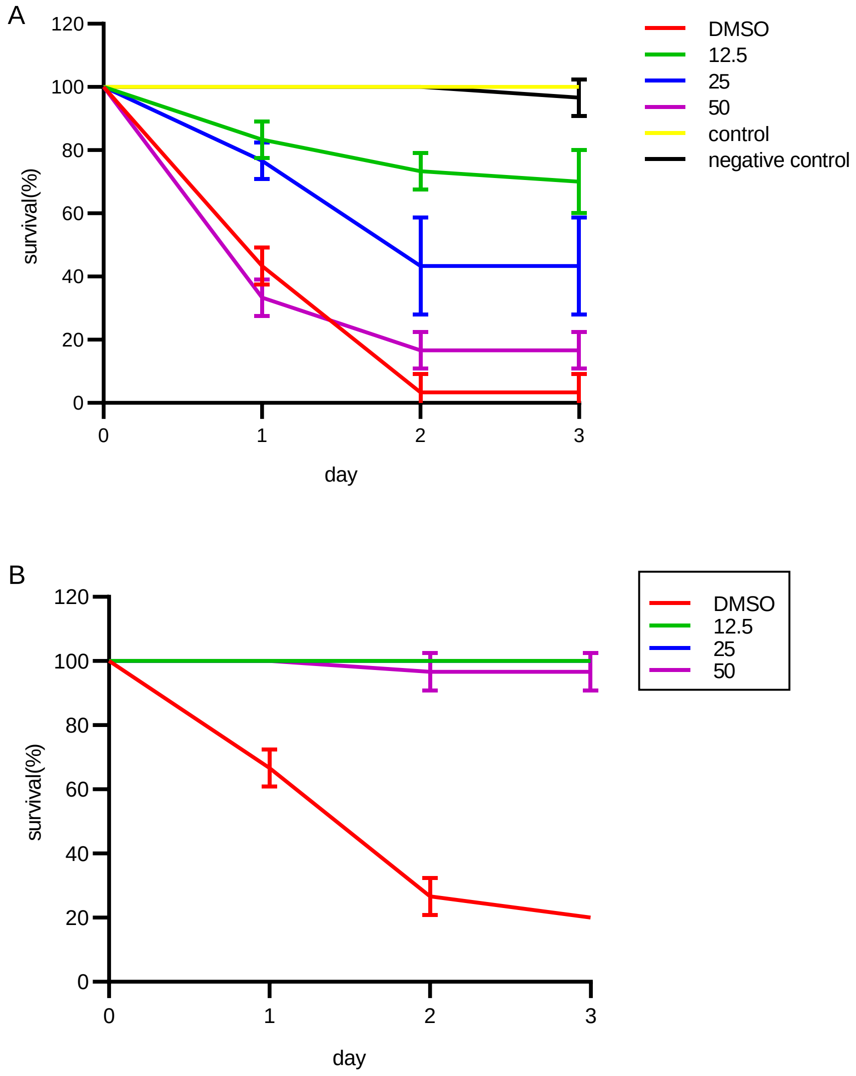

Figure S11. Mortality of *T. molitor* larvae injected *S. marcescens* 4547 (A) and *S. marcescens* NJ01 (B) and treated with m-NPe at 12.5, 25 and 50 μg/mL. DMSO: DMSO injected together with bacteria. Control: DMSO injected without bacteria. Negative control: 50 μg/mL m-NPe injected without bacteria. Results are expressed as mean ± SD, n=3.

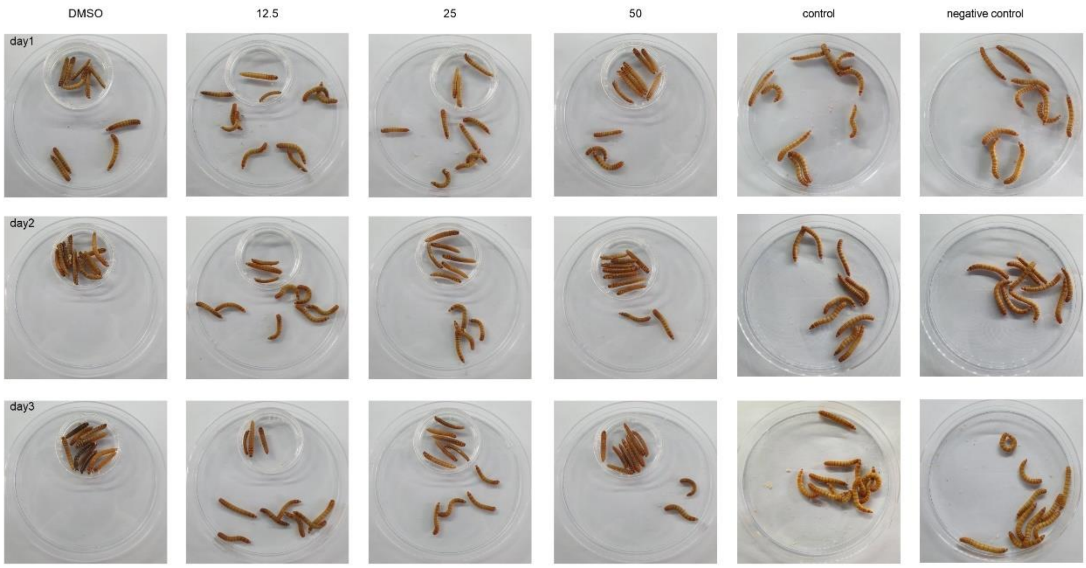
DMSO
12.5
25
50
control
negative control
day1
day2
day3

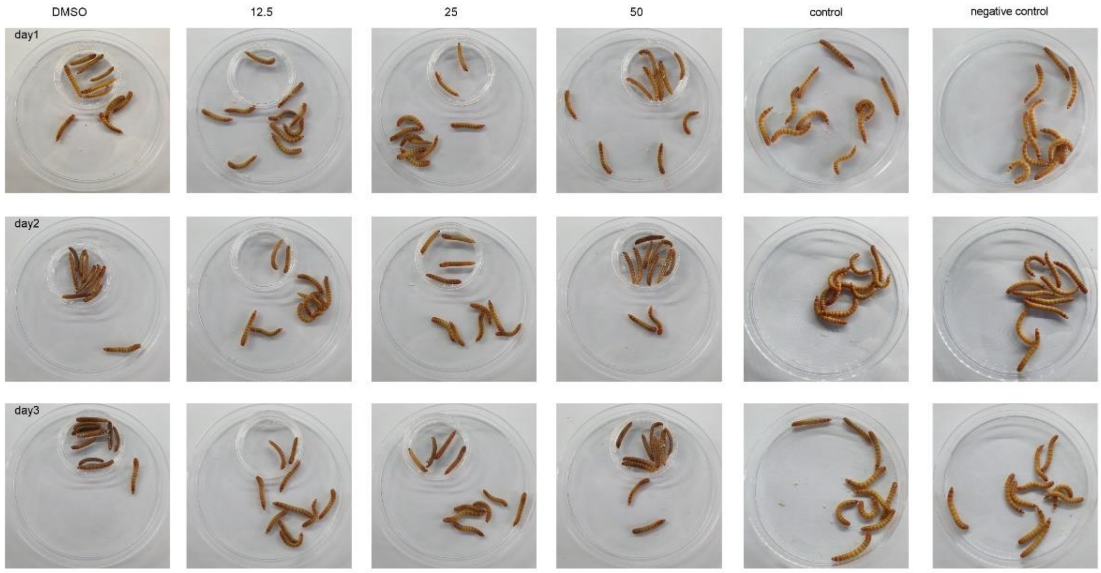
DMSO
12.5
25
50
control
negative control
day1
day2
day3

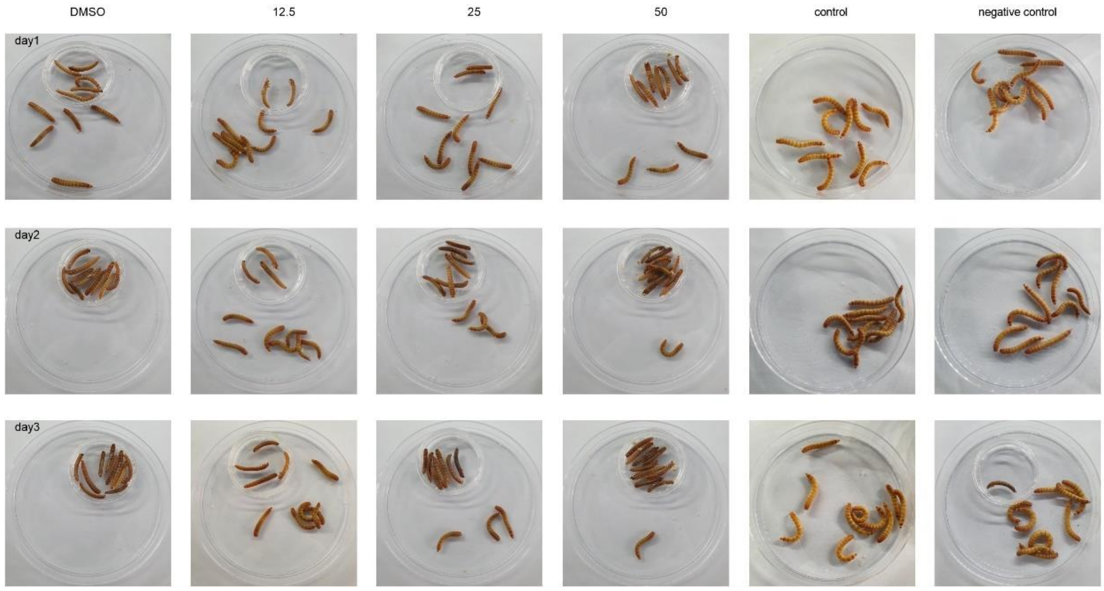
DMSO
12.5
25
50
control
negative control
day1
day2
day3

Figure S12 (A). Representative figures showing the mortality of *T. molitor* larvae injected *S. marcescens* 4547 and treated with m-NPe at 12.5, 25 and 50 μg/mL. DMSO: DMSO injected with bacteria. Control: DMSO injected without bacteria. Negative control: 50 μg/mL m-NPe injected without bacteria. Results are expressed as mean ± SD, n=3.

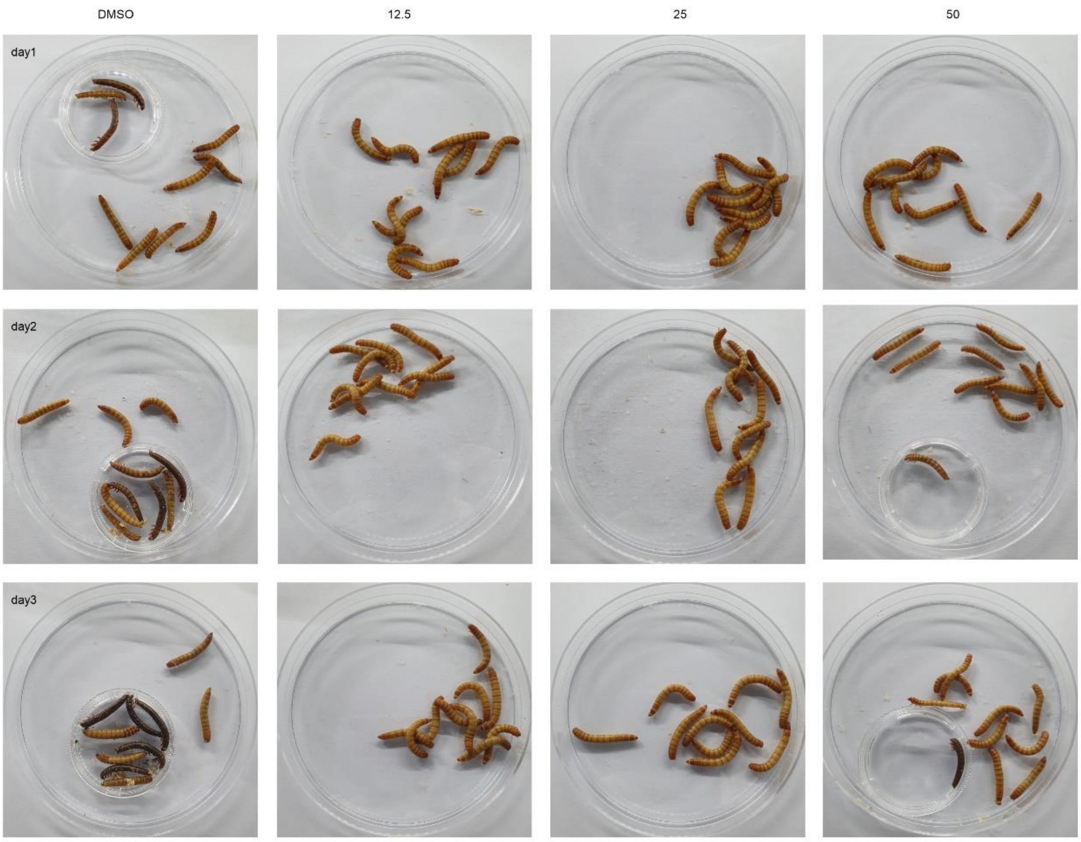
DMSO
12.5
25
50
day1
day2
day3

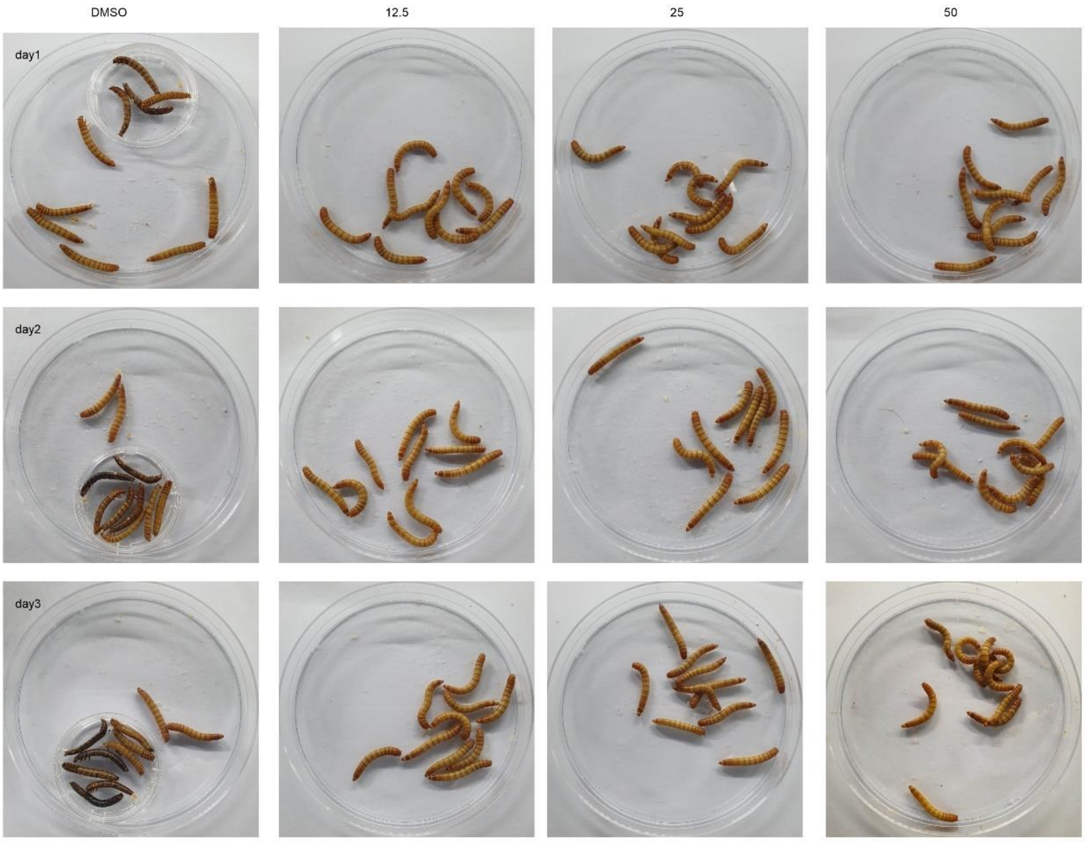
DMSO
12.5
25
50
day1
day2
day3

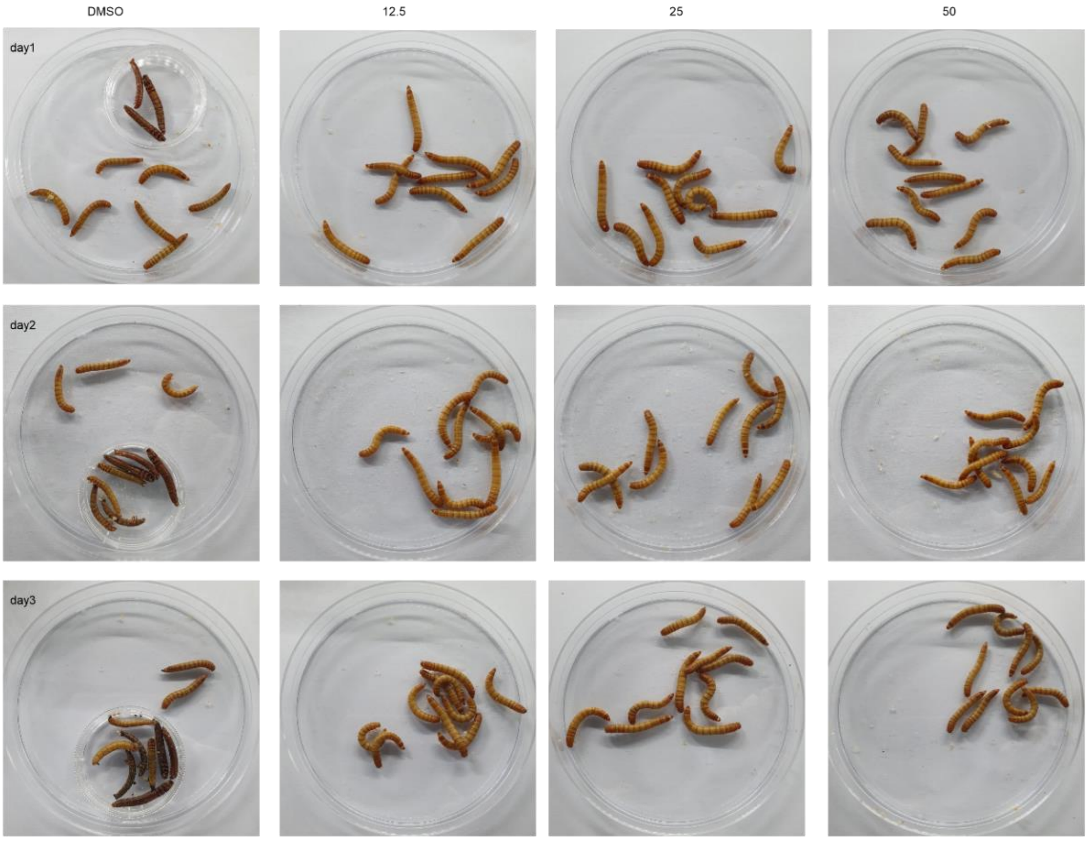

Figure S12 (B). Representative figures showing the mortality of *T. molitor* larvae injected *S. marcescens* NJ01 and treated with m-NPe at 12.5, 25 and 50 μg/mL. DMSO: DMSO injected with bacteria. Control: DMSO injected without bacteria. Negative control: 50 μg/mL m-NPe injected without bacteria. Results are expressed as mean ± SD, n=3.

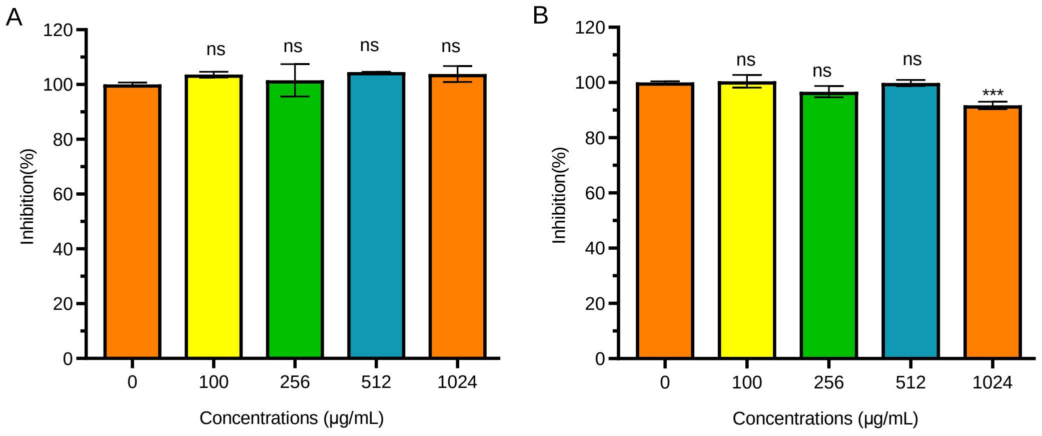

Figure S13. Inhibitory effects of m-NPe on mouse embryonic fibroblast (MEF) (A) and mouse erythrocytes (B). Compounds were dissolved in DMSO which was used as a control. Results are expressed as mean ± SD, n=3. ns: not significant, analyzed by one-way ANOVA, followed by Tukey-Kramer post-hoc test for multiple comparisons.

Methods

1.1 Methods of virulence factor assay

S. marcescens NJ01 cultured overnight were 1:100 (v/v) added to LB medium (1:100, v/v) containing m-NPe (12.5-50 μg/mL), and cultivated at 28 °C while rotation at 180 rpm. Dimethyl sulfoxide (DMSO) and vanillic acid (VAN) (50 μg/mL) served as negative and positive controls, respectively. After incubation, they were centrifuged for 5 min at 4 °C. The supernatants were stored at -20 °C for further use.

For determination of protease activity[1], 75 μL of the above supernatants were mixed with 125 μL of 2% azocasein dissolved in 1M Tris-HCl (pH 8.0), and incubated at 37 °C for 15 min. After incubation, 600 μL of 10% trichloroacetic acid were added to each tube, 30 min later, the mixtures were centrifuged. The reactions were terminated by adding 700 μL of 1M NaOH. The activities of the protease were determined by measuring OD_{440} values.

For determination of lipase activity [2], 100 μL of the supernatants were added to 900 μL of the substrate solution made up by mixing 1 volume of 0.3% (w/v) *p*-nitrophenyl palmitate (*p*-NPP) dissolved in isopropanol and 9 volumes of 0.2% (w/v) sodium deoxycholate and 0.1% (w/v) gummi arabicum dissolved in 50 mM Na_2PO_4 buffer, and incubated in the dark for 1 h. The solutions were cleared by centrifugation, and the OD_{410} values of the supernatants were measured.

For determination of hemolysin [3], 100 μL of the supernatants were mixed with 900 μL of sheep's blood cell suspension and incubated at 37 °C for 1 h. The OD_{530} values of the supernatants were measured after centrifugation.

To measure EPS [4], *S. marcescens* was incubated at 28 °C while rotated at 180 rpm for 24 h in 24-well plates. Biofilms were washed with distilled water, followed by addition of 500 μL 0.9% NaCl, 5% phenol, and 2.5 mL of 0.2% hydrazine sulfate. After incubated for 1 h in the dark, OD_{490} values of the solution were measured.

To determine hydrophobicity index [5], 1 mL of *S. marcescens* NJ01 suspension

was added to tubes, and an equal volume of toluene was added. The mixture was vortexed for 3 min and incubated for 15 min at room temperature for phase separation. Then, the OD_{530} of the aqueous phase was measured, and the hydrophobicity index was calculated.

In prodigiosin assay [6], the *S. marcescens* was grown in the absence or presence of m-NPe at 30 °C for 20 h and centrifuged at 12,000 rpm for 10 min. Acidified ethanol containing 40 mM HCl was added to the cell pellets, and vortexed vigorously to extract prodigiosin. After centrifugation, The OD_{534} values of the supernatants were measured.

Effects of m-NPe on *S. marcescens'* fimbria activity were accessed using *Saccharomyces cerevisiae* (Sigma, product No. YSC2) as previously described [7]. Briefly, yeast agglutination was measured spectrophotometrically by adding 1.5 mL of PBS containing 0.5 mL of *S. cerevisiae* (2% w/v in PBS) and 0.4 mL of *S. marcescens* cells in PBS (OD_{600} = 0.5). To achieve a uniform mixture of *Saccharomyces cerevisiae* and *S. marcescens* suspension, the reaction tubes were gently vortexed for 5 s and the initial OD_{600} was measured. After 10 min of incubation at room temperature, 100 mL of cells suspension was transferred to a 96-well plate and OD_{600} was measured. Formation of the agglutination of *S. marcescens* with *S. cerevisiae* indicates the presence of fimbria on *S. marcescens* cell surface. After vigorously vortexed for 30 s, the second OD_{600} values were measured. Percentage of agglutination was calculated using the equation: Percentage = 100 × (1 - $OD_{600,1st}$ / $OD_{600, 2nd}$).

H_2O_2 levels were determined according to previous publication by Gonzalez-Flecha and Demple with minor modification [8]. Briefly, bacteria were pelleted and resuspended in PBS. After 1-min centrifugation at 6,000 g, the suspensions were used for H_2O_2 measurement employing the horseradish peroxidase-scopoletin method.

For swarming motility [9], 1 μL of bacteria suspension was inoculated in the swarming medium. The plates were cultivated at 28 °C overnight, then swarming was analyzed.

For swimming motility assay [10], 1 μL of bacterial culture was inoculated in the swimming medium. The plates were cultivated at 28 °C, overnight, then swimming was determined.

For detection of prodigiosin [11], cell pellets were washed and resuspended with the same volume of acidified ethanol (4 mM HCl), followed by centrifugation for 5 min. Then, the pigments levels were determined by measuring OD_{534} values.

1.2 Determination of MICs and growth curves

The minimum inhibitory concentrations (MICs) of *β*-nitrostyrene derivatives against the *S. marcescens* was determined by broth microdilution, following the Clinical and Laboratory Standards Institute (CLSI 2015) guidelines with minor modifications. The strain was inoculated in 5 mL of LB medium (Sangon Biotech Co., Ltd., Shanghai, China) for 17 h, then adjusted to OD_{620} = 0.05, with a resulting bacterial concentration of about 1.5×10^5 CFU/mL. The *β*-nitrostyrene derivatives were then added, and the mixtures were cultured at 28°C, 150 rpm for 24 h. DMSO was used as a negative control. For each treatment, 200 μL suspension was transferred to 96-well plates (Costar™ 3599, Corning Inc., Corning, NY, USA), which were then cultured at 28 °C, 150 rpm for 24 h. OD_{620} was measured using a microplate reader (BioTek ELx800, BioTek, Winooski, VT, USA). Three replicates were performed for each test.

1.3 Protein purification and analysis.

cDNA of SmaR was PCR-amplified using the primers listed in Table S5 and cloned to the expression vector pGEX-6p-1 (Amersham). The constructed plasmid was transformed into the *E. coli* BL21 strain. Affinity purification of SmaR with the GST tag was performed using the method described previously [12]. The GST tag was cleaved using the PreScission Protease (GE Healthcare; 2 units/100 pL of bound proteins) at 4 °C overnight. The cleaved proteins were eluted and analyzed by SDS-PAGE.

1.4 Analysis of the interaction of m-NPe with proteins by microscale thermophoresis (MST) assay

MST assay was used to quantify m-NPe's binding affinities to SmaR protein as previously published with minor modifications [13]. In brief, recombinant protein SmaR with a His-tag were purified from *E. coli* BL21 strain. 100 μL of 10 μM purified SmaR was added into the labeling buffer, and labeled with the dye Monolith NT.115 (NanoTemper Technologies GmbH) at room temperature for 30 min in the dark. The labeled protein was diluted in MST buffer containing 50 mM Tris-HCl (pH 8.0), 150 mM NaCl, and 0.05% (v/v) Tween-20. Sixteen micro reaction tubes were labeled from 1 to 16. m-NPe (final conc. 250 μM) diluted in MST buffer (20 μL) was added to the first micro reaction tube, and 16 serial dilutions (1:2) were made. 10 μL of labeled protein was added to the diluted m-NPe solution and incubated at room temperature for 10 min, then loaded into silica capillaries (Polymicro Technologies). Binding reactions were measured using a microscale thermophoresis instrument (NanoTemper Technologies GmbH) at 25 °C, 40% MST power and 20% LED power. The MO affinity analysis (V2.3) software was used to fit the curve and calculate the value of the dissociation constant (K_d).

1.5 The experiment methods for cytotoxicity.

Mouse embryonic fibroblasts (MEFs) or mouse erythrocytes were purchased from Type Culture Collection (ATCC, Rockefeller, Maryland, USA). Cells were cultured in Dulbecco's modified Eagle's medium containing 10% fetal bovine serum (Invitrogen, Shanghai, China) and 10 U/mL penicillin/streptomycin at 37 °C. The effects of compounds on cell viability were assessed by MTT method after treating cells for 24 h (Beyotime, Cat# C0009, Chengdu, China). Briefly, after treatments, cells were incubated with 0.5 mg/mL MTT at 37 °C for 4 h, the supernatant was discarded, and cells were resuspended in DMSO solution, and OD_{490} were measured.

1.6 Molecular docking

The three-dimensional structures of SmaR [16], RhlI (PDB ID: 4NG2), RhlR (PDB ID: 7KGW), LasR (PDB ID: 3RKR), and CviR (PDB ID: 3QP5), were retrieved from Protein data bank (PDB). These target proteins were individually docked with m-NPe to the binding pocket for their native ligands using the AutoDock

software[16,18]. Chemical structures of all ligands were constructed using ChemDraw (version 15.1).

1.7 Molecular dynamic studies on binding stability and interaction energy

Molecular dynamics analyses were made using the Groningen Machine for Chemical Simulation (GROMACS, 2020.03) software installed on a computer with the Ubuntu (18.06) Linux OS and nVidia CUDA hybrid parallel computational environment. In the analyses, the Amber99SB force field and TIP3P explicit water model was adopted. Each target protein was positioned in the center of a dodecahedron unit cell, with a spacing of 3.0 nm between the proteins and the edge. The cells were filled with H_2O, then neutralized by random replacing water molecules with Na^+ and Cl^-, the final concentration of NaCl was 0.1 M. After a brief energy minimization followed by temperature and pressure equilibration, the MD formal analysis was carried out. Each formal analysis consists of 50,000,000 time steps, generating 8 Gb data approximately. The root mean square deviation (RMSD) of the position of the heavy atoms in a ligand was calculated to evaluate the binding stability of the ligands [20,21]. The interaction energy between a protein and a ligand is the sum of Lennard-Jones potential and Coulombic energy [22,23].

1.8 Evaluation of mortality of *T. molitor* larvae

In the mortality assay, the yellow mealworm (*Tenebrio molitor*) was used as a model [24] in this study to evaluate cellular toxicity of the m-NPe against *S. marcescens*. In brief, *S. marcescens* was cultured overnight and centrifuged. Supernatants were filtered through a 0.22 μm filter, and 20 uL of the filtered supernatant was injected into the larvae. The temperature and relative humidity were maintained at 25-28 °C and 60-70%, respectively. Yellow mealworms of the same instar stage were used for evaluation of survival rates under different treatments for 3 d. During the period, larval mortality was recorded. Dead larvae were removed from all treatments every day and recorded for analysis. Experiments were repeated at least three times.

References

1. Coulthurst SJ, Williamson NR, Harris AKP, Spring DR, Salmond GPC. Metabolic and regulatory engineering of *Serratia marcescens*: mimicking phage-mediated horizontal acquisition of antibiotic biosynthesis and quorum-sensing capacities. *Microbiol.* 2006; 152:1899–911.

2. Patel U, Chandpura J, Chauhan K, Gupte S. Screening and Isolation of an Organic Solvent Tolerant Lipase Producing Bacteria from Various Oil Contaminated Sites. *IJAM*. 2018; 21:22–36.

3. Padmavathi AR, Abinaya B, Pandian SK. Phenol, 2,4-bis(1,1-dimethylethyl) of marine bacterial origin inhibits quorum sensing mediated biofilm formation in the uropathogen *Serratia marcescens*. *Biofouling*. 2014; 30:1111–22.

4. Badireddy AR, Korpol BR, Chellam S, Gassman PL, Engelhard MH, Lea AS, Rosso KM. Spectroscopic characterization of extracellular polymeric substances from Escherichia coli and *Serratia marcescens*: suppression using sub-inhibitory concentrations of bismuth thiols. *Biomacromolecules*. 2008; 9:3079–89.

5. Sethupathy S, Sathiyamoorthi E, Kim Y-G, Lee J-H, Lee J. Antibiofilm and Antivirulence Properties of Indoles Against *Serratia marcescens*. *Front. Microbiol.* 2020.

6. Slater H, Crow M, Everson L, Salmond GPC. Phosphate availability regulates biosynthesis of two antibiotics, prodigiosin and carbapenem, in *Serratia* via both quorum-sensing-dependent and -independent pathways. *Mol. Microbiol.* 2003; 47:303–20.

7. Shanks RMQ, Stella NA, Kalivoda EJ, Doe MR, O'Dee DM, Lathrop KL, et al. A *Serratia marcescens* OxyR homolog mediates surface attachment and biofilm formation. *J. Bacteriol.* 2007; 189:7262–72.

8. B. Gonzalez-Flecha, B. Demple, Homeostatic regulation of intracellular hydrogen peroxide concentration in aerobically growing *Escherichia coli*, *J. Bacteriol*. 1997 179 382–388.

9. Pearson MM. Methods for Studying Swarming and Swimming Motility. *Methods Mol Biol.* 2019; 2021:15–25.

10. P.M. Merritt, T. Danhorn, C. Fuqua, Motility and chemotaxis in *Agrobacterium tumefaciens* surface attachment and biofilm formation, *J. Bacteriol.* 2007 8005–8014.

11. R. Salini, S.K. Pandian, Interference of quorum sensing in urinary pathogen *Serratia marcescens* by Anethum graveolens, *Pathog. Dis.* 2015 73 ftv038.
12. He, Y.‐W., Boon, C., Zhou, L., and Zhang, L.‐H. Co‐regulation of Xanthomonas campestris virulence by quorum sensing and a novel two‐component regulatory system RavS/RavR, *Molecular microbiology* 2009 71, 1464–1476.
13. Gao, M., He, Y., Yin, X., Zhong, X., Yan, B., Wu, Y., Chen, J., Li, X., Zhai, K., and Huang, Y. Ca2+ sensor-mediated ROS scavenging suppresses rice immunity and is exploited by a fungal effector, *Cell* 2021 184, 5391-5404.
14. Bakkiyaraj D, Sivasankar C, Pandian SK. Inhibition of quorum sensing regulated biofilm formation in *Serratia marcescens* causing nosocomial infections. *Bioorg & Med Chem Lett.* 2012; 22(9):3089–94.
15. Chen G, Swem LR, Swem DL, Stauff DL, O'Loughlin CT, Jeffrey PD, et al. A strategy for antagonizing quorum sensing. *Mol. Cell* 2011; 42(2):199–209.
16. Khayyat AN, Hegazy WAH, Shaldam MA, Mosbah R, Almalki AJ, Ibrahim TS, et al. Xylitol inhibits growth and blocks virulence in *Serratia marcescens*. *Microorganisms* 2021; 9(5).1083
17. Ramanathan S, Ravindran D, Arunachalam K, Arumugam VR. Inhibition of quorum sensing-dependent biofilm and virulence genes expression in environmental pathogen *Serratia marcescens* by petroselinic acid. *Antonie van Leeuwenhoek* 2018; 111(4):501–15.
18. Liu L, Zeng X, Zheng J, Zou Y, Qiu S, Dai Y. AHL-mediated quorum sensing to regulate bacterial substance and energy metabolism: A review. *Microbiological Research* 2022; 262:127102.
19. Fan H, Dong Y, Wu D, Bowler MW, Zhang L, Song H. QsIA disrupts LasR dimerization in antiactivation of bacterial quorum sensing. PANS 2013; 110(51):20765–70.
20. Pronk S, Pall S, Schulz R, Larsson P, Bjelkmar P, Apostolov R, et al. GROMACS 4.5: a high-throughput and highly parallel open source molecular simulation toolkit. *Bioinformatics* 2013; 29(7):845–54.
21. Markidis S, Laure E. Solving software challenges for exascale, 8759. Cham: *Springer International Publishing;* 2015. 25p.

22. Abraham MJ, Murtola T, Schulz R, Páll S, Smith JC, Hess B, et al. GROMACS: High performance molecular simulations through multi-level parallelism from laptops to supercomputers. *SoftwareX* 2015; 1-2:19–25.
23. Sun Y, Wang BC, Pei JL, Luo Y, Yuan N, Xiao ZP, et al. Molecular dynamic and pharmacological studies on protein-engineered hirudin variants of *Hirudinaria manillensis* and *Hirudo medicinalis*. *British J Pharmocol.* 2022; 179(14):3740–53.
24. Jin L, Feng P, Cheng Z, Di Wang. Effect of biodegrading polyethylene, polystyrene, and polyvinyl chloride on the growth and development of yellow mealworm (*Tenebrio molitor*) larvae. *Envir Sci. Poll. Res. Int.* 2023; 30(13):37118–26.

10. NMR spectra of compounds.

^{1}H NMR (400 MHz, $CDCl_3$) spectrum of compound 4

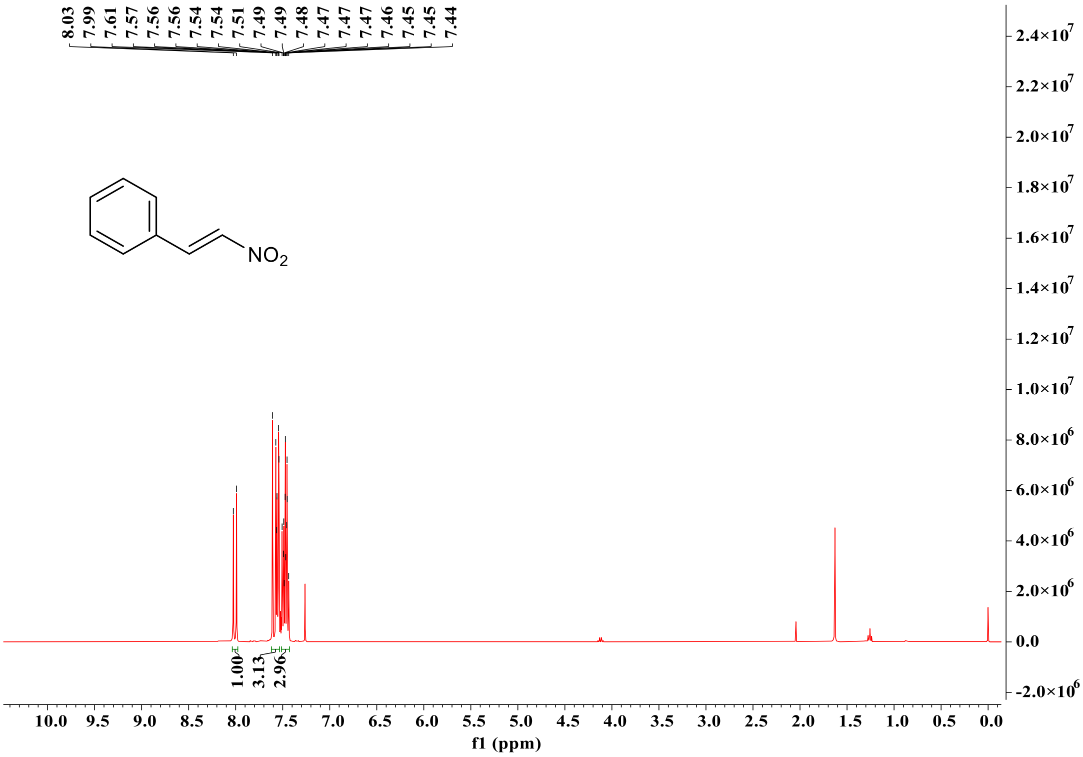

^{1}H NMR (400 MHz, $CDCl_3$) spectrum of compound 5

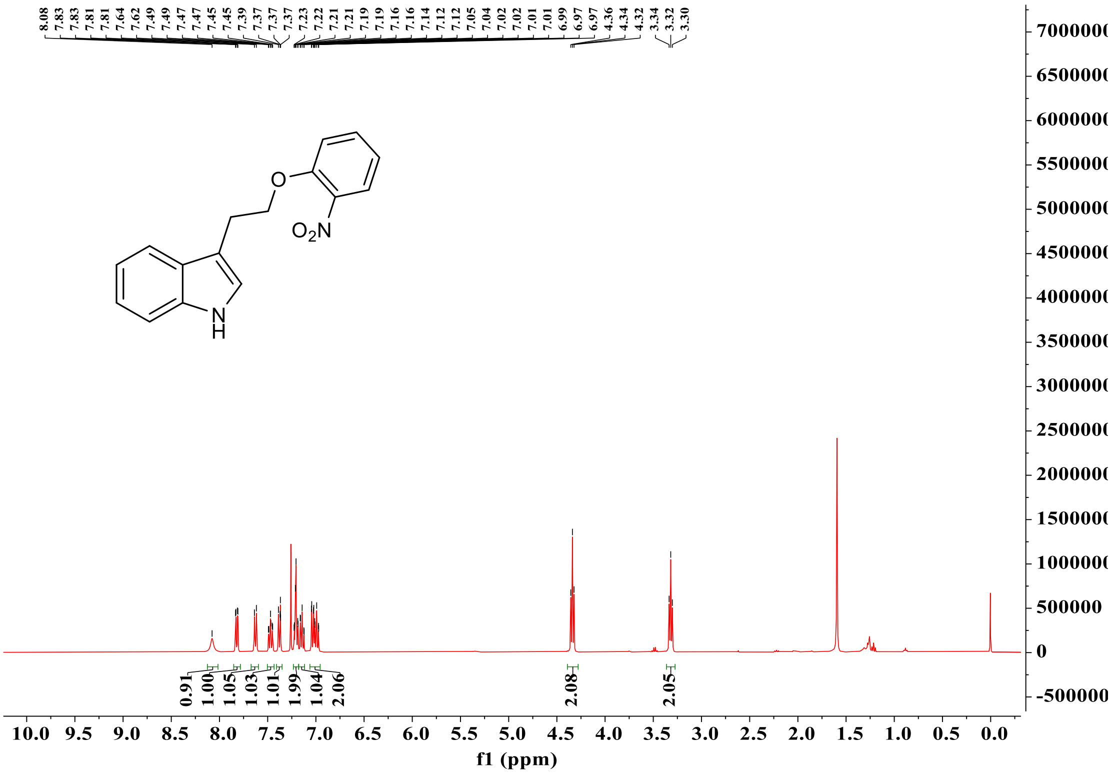

^{13}C NMR (100 MHz, $CDCl_3$) spectrum of compound 5

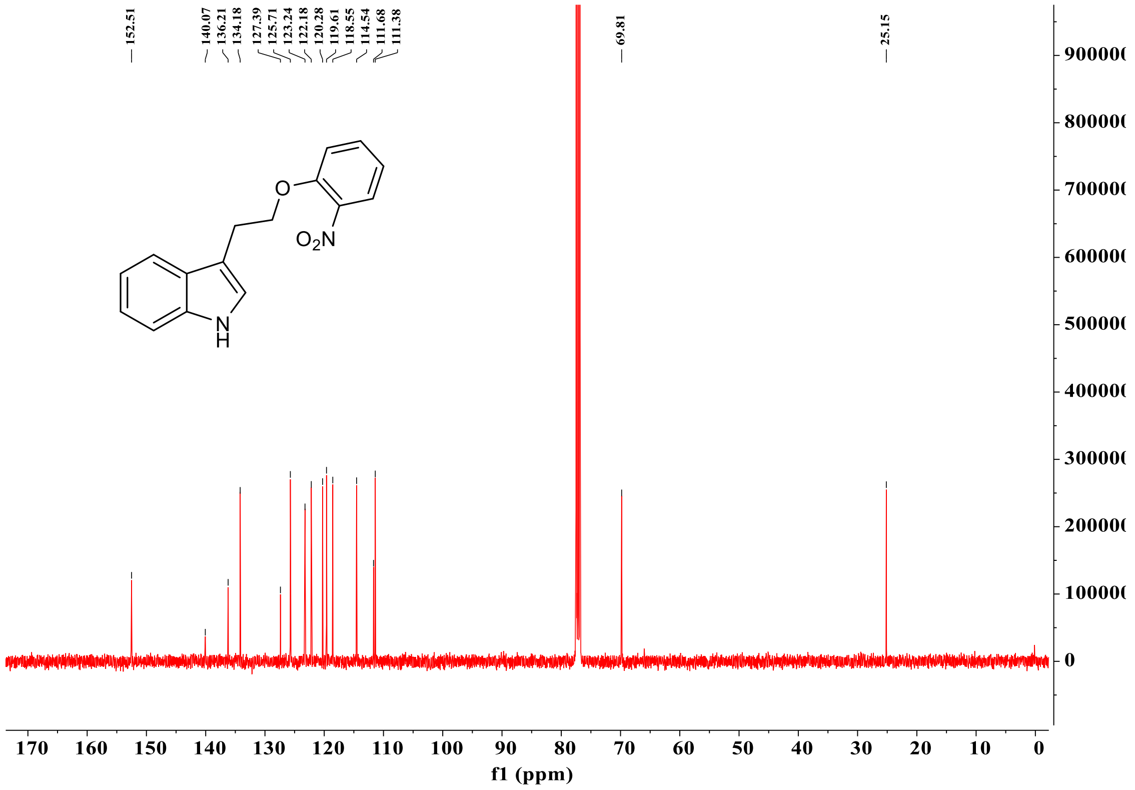

^{1}H NMR (400 MHz, $CDCl_3$) spectrum of compound 6

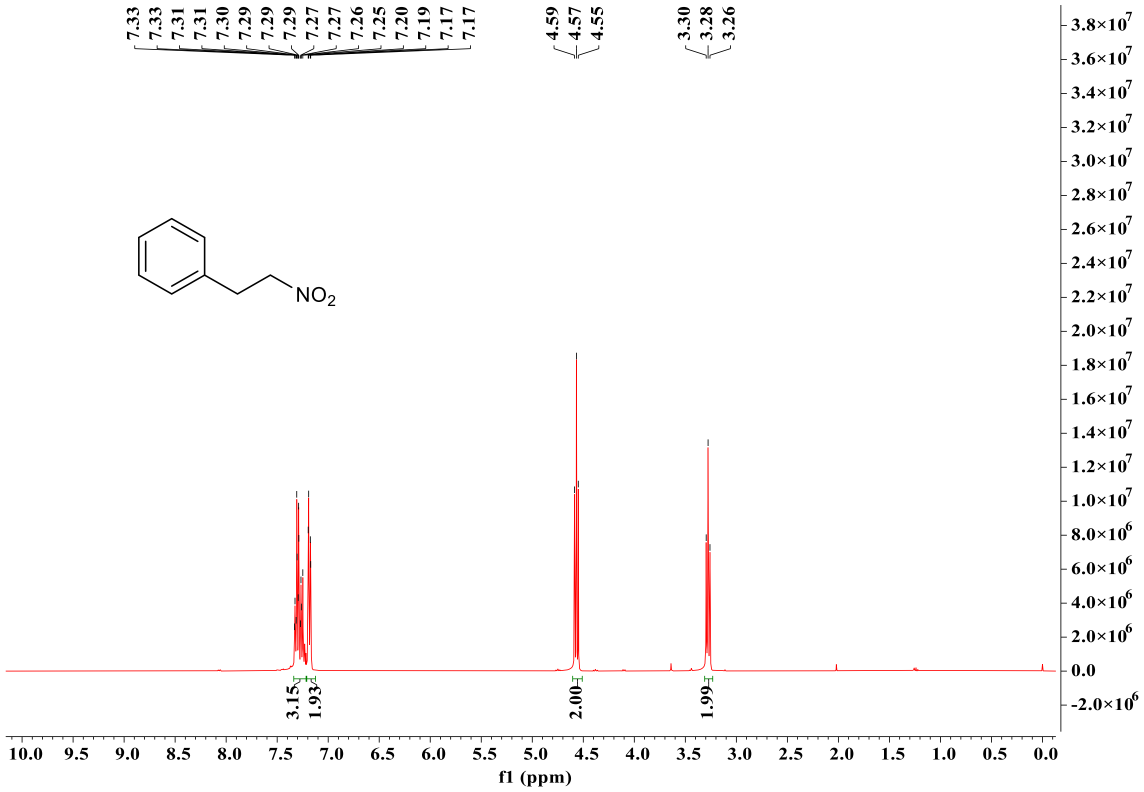

^1H NMR (400 MHz, $CDCl_3$) spectrum of compound 7

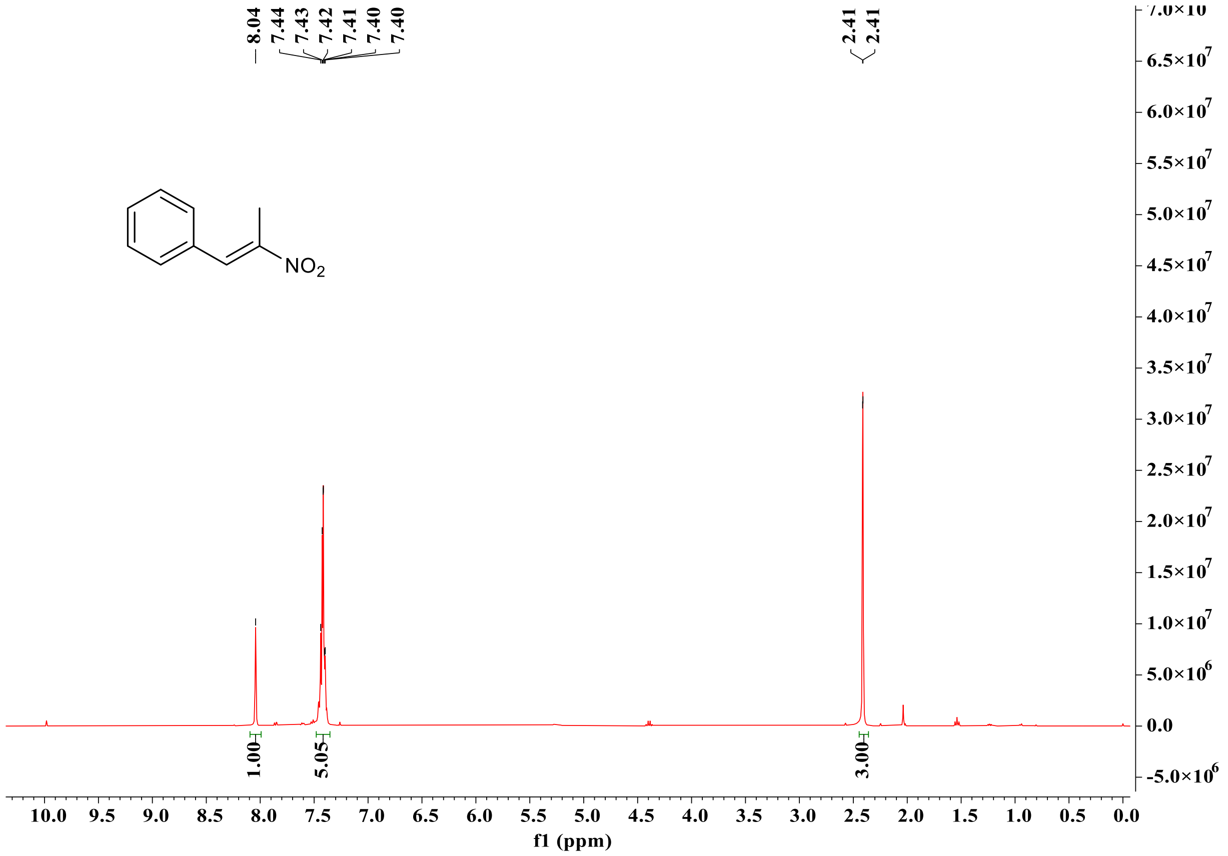

^1H NMR (400 MHz, $CDCl_3$) spectrum of compound 8

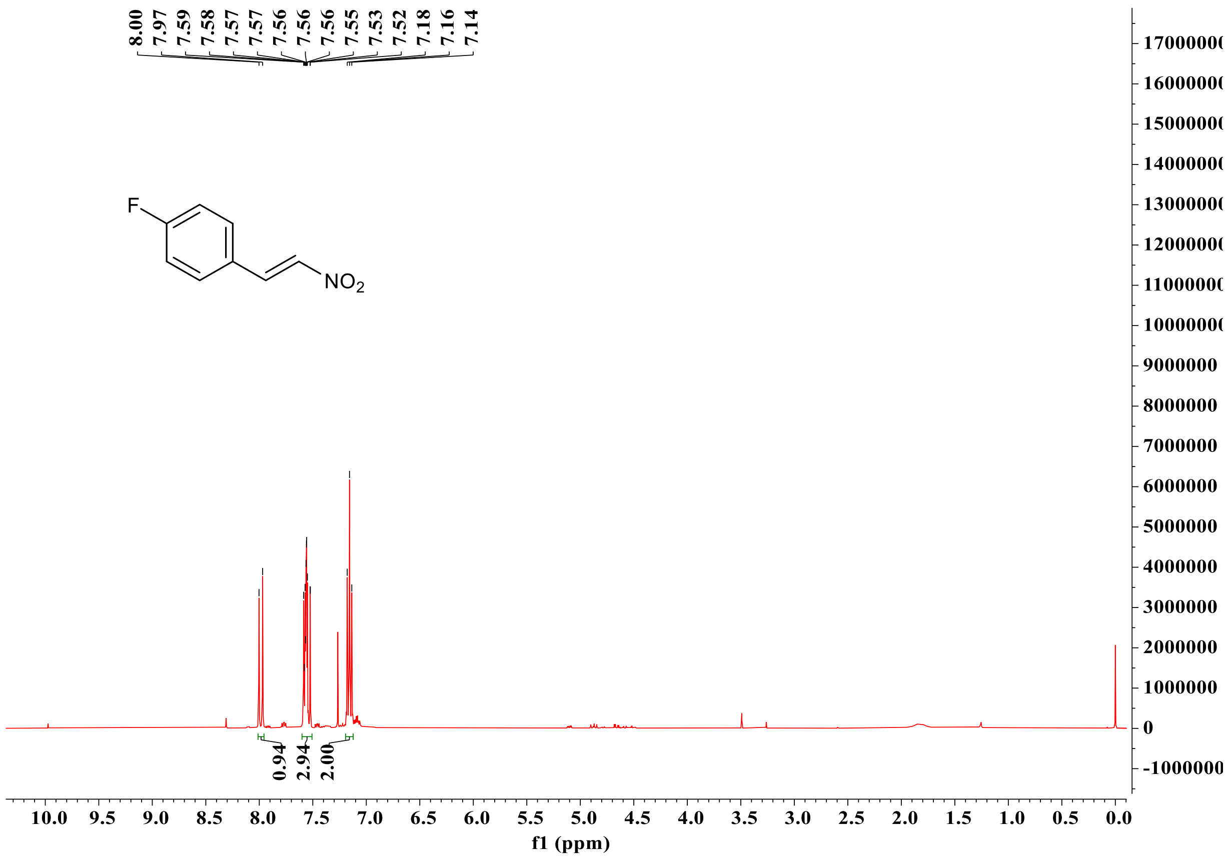

^1H NMR (400 MHz, $CDCl_3$) spectrum of compound 9

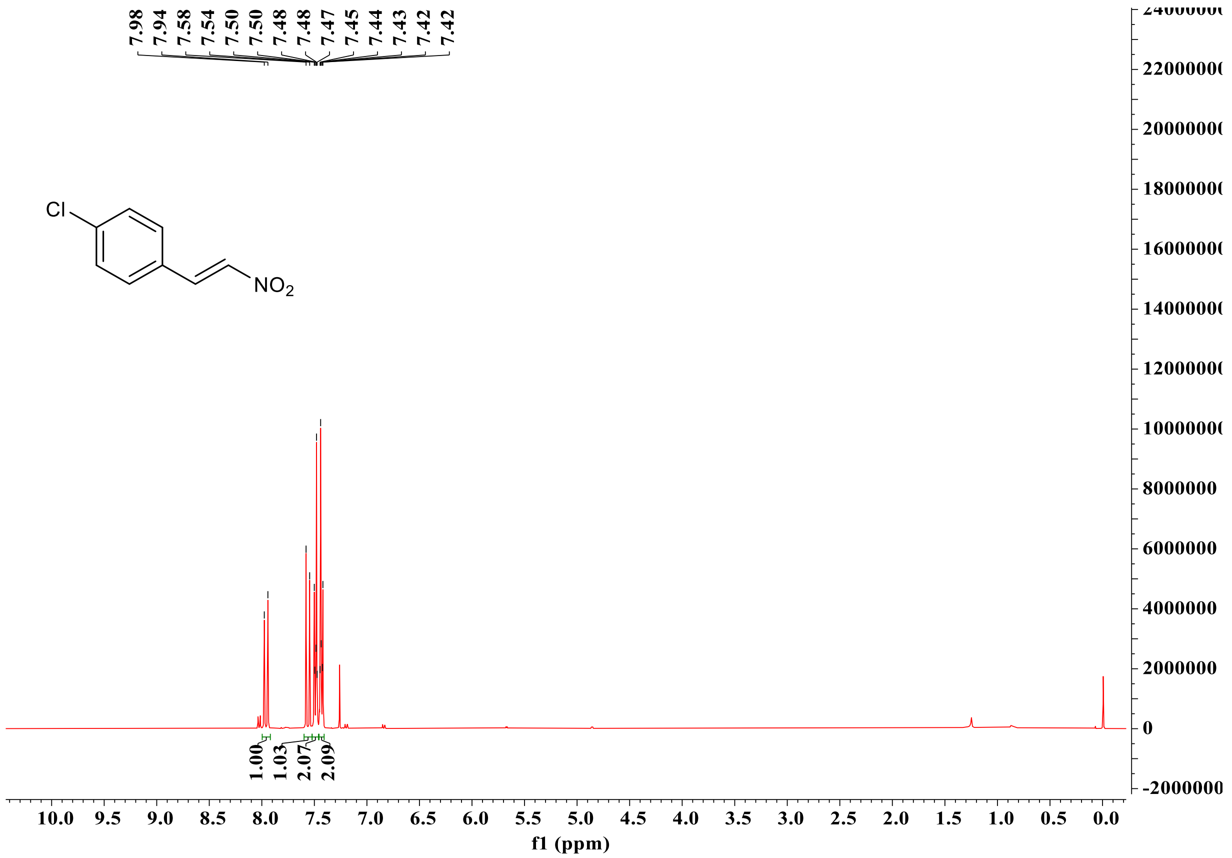

^1H NMR (400 MHz, $CDCl_3$) spectrum of compound 10

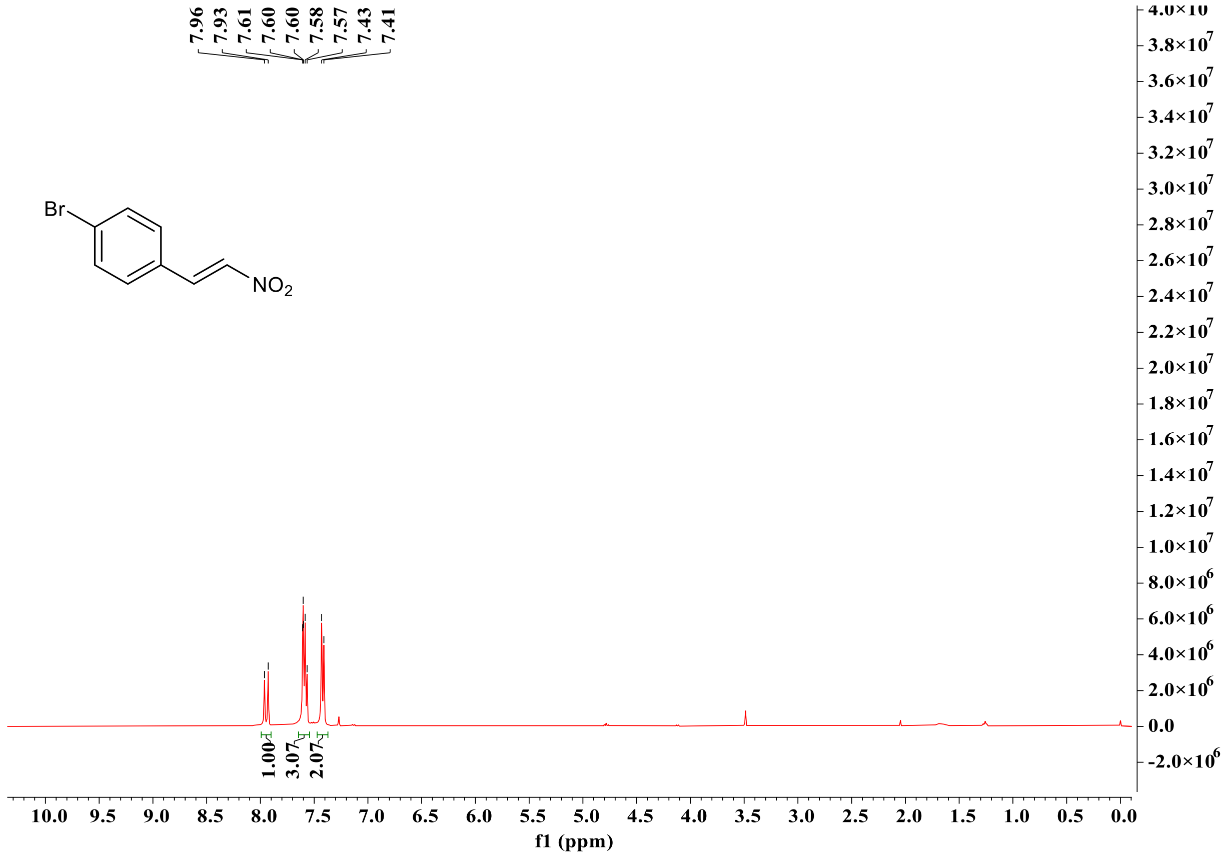

^{1}H NMR (400 MHz, $CDCl_3$) spectrum of compound 11

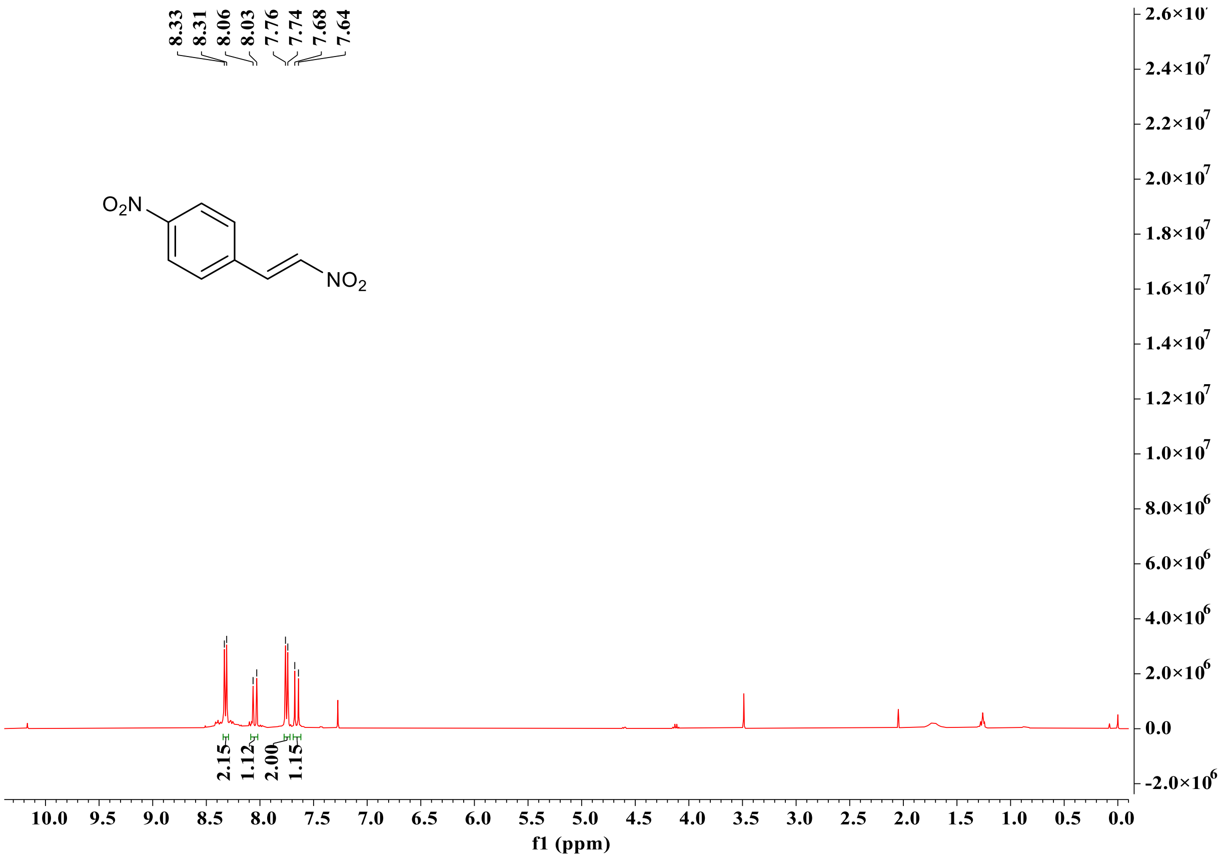

^{1}H NMR (400 MHz, MeOH) spectrum of compound 12

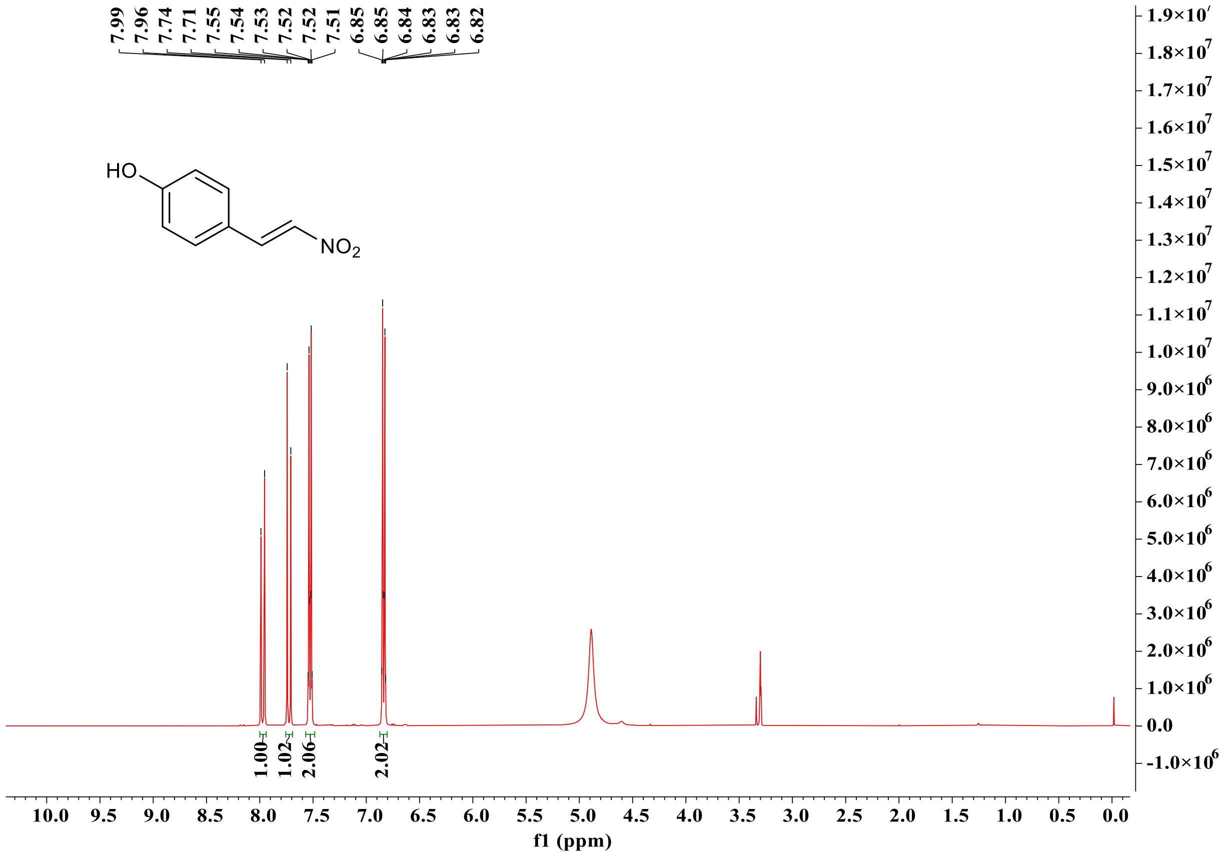

^{1}H NMR (400 MHz, $CDCl_3$) spectrum of compound 13

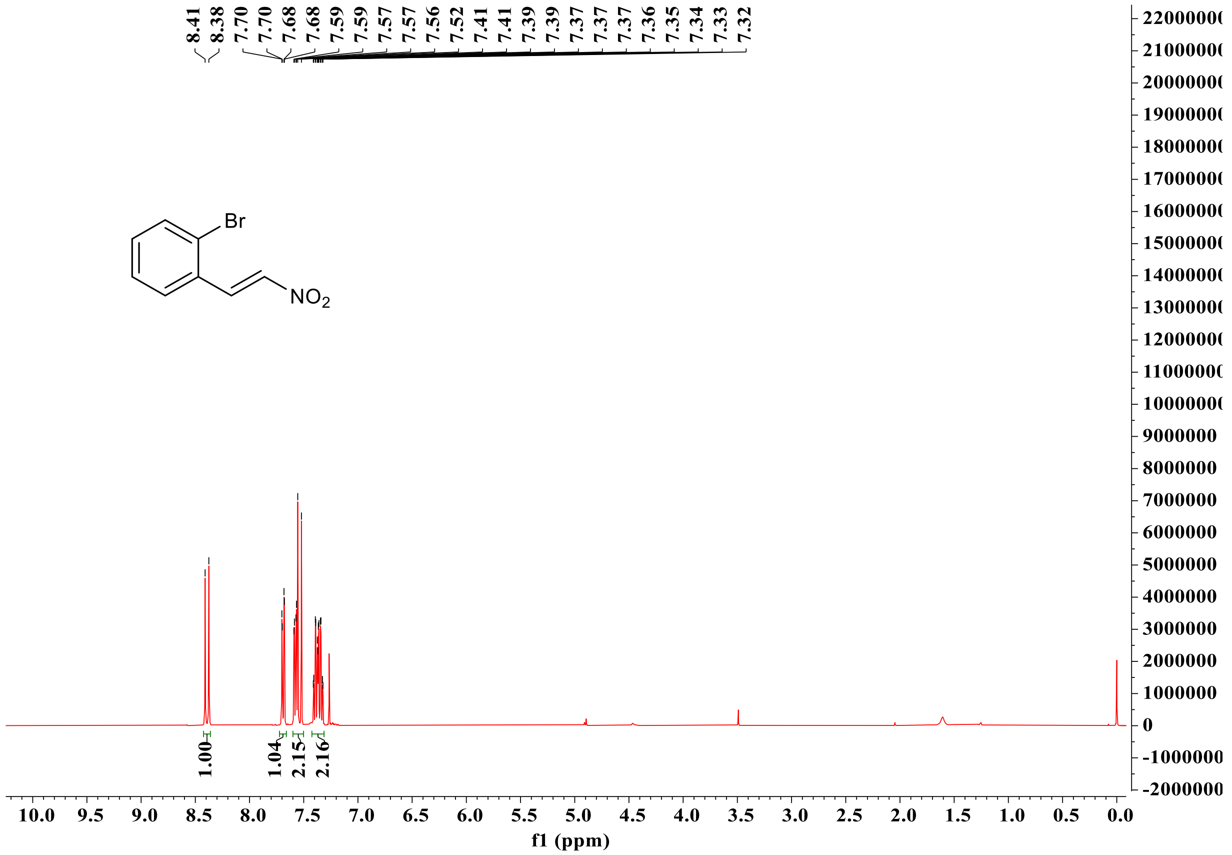

^{1}H NMR (400 MHz, $CDCl_3$) spectrum of compound 14

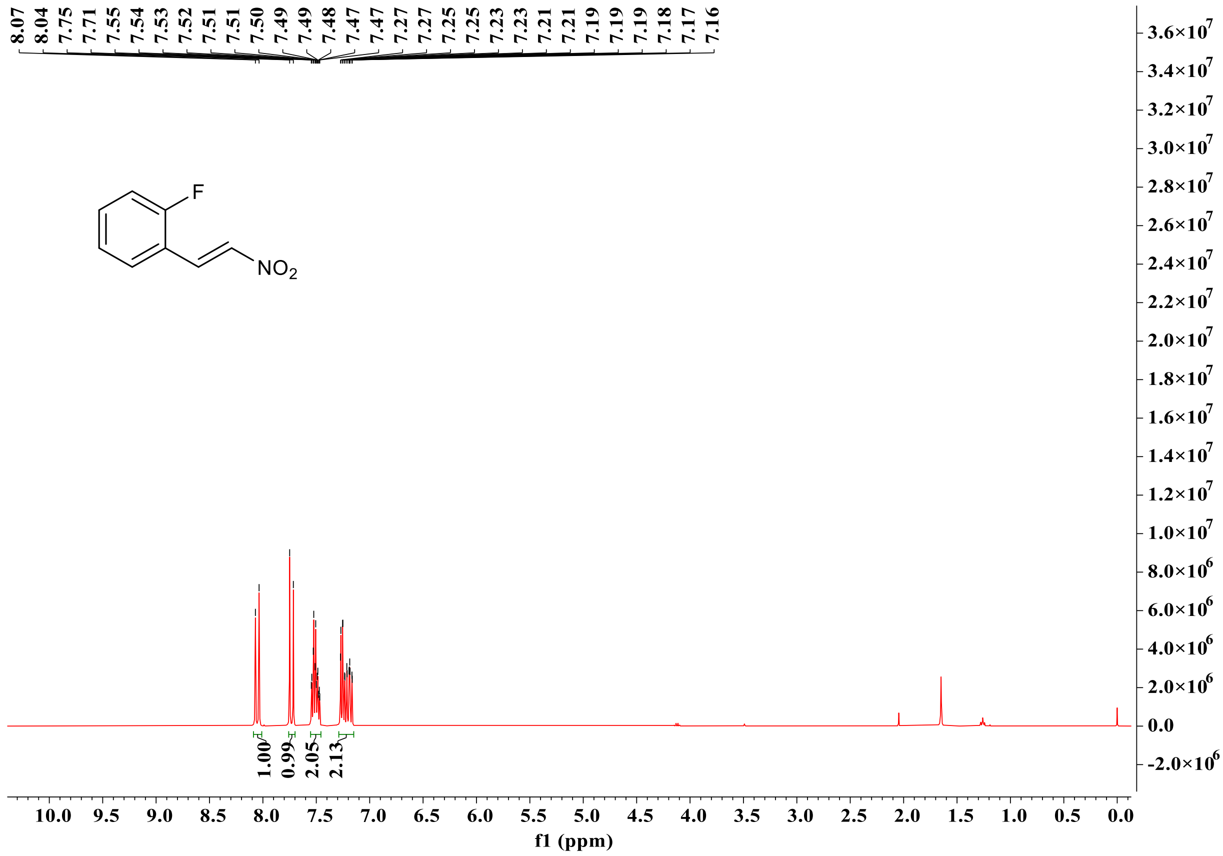

^{1}H NMR (400 MHz, $CDCl_3$) spectrum of compound 15

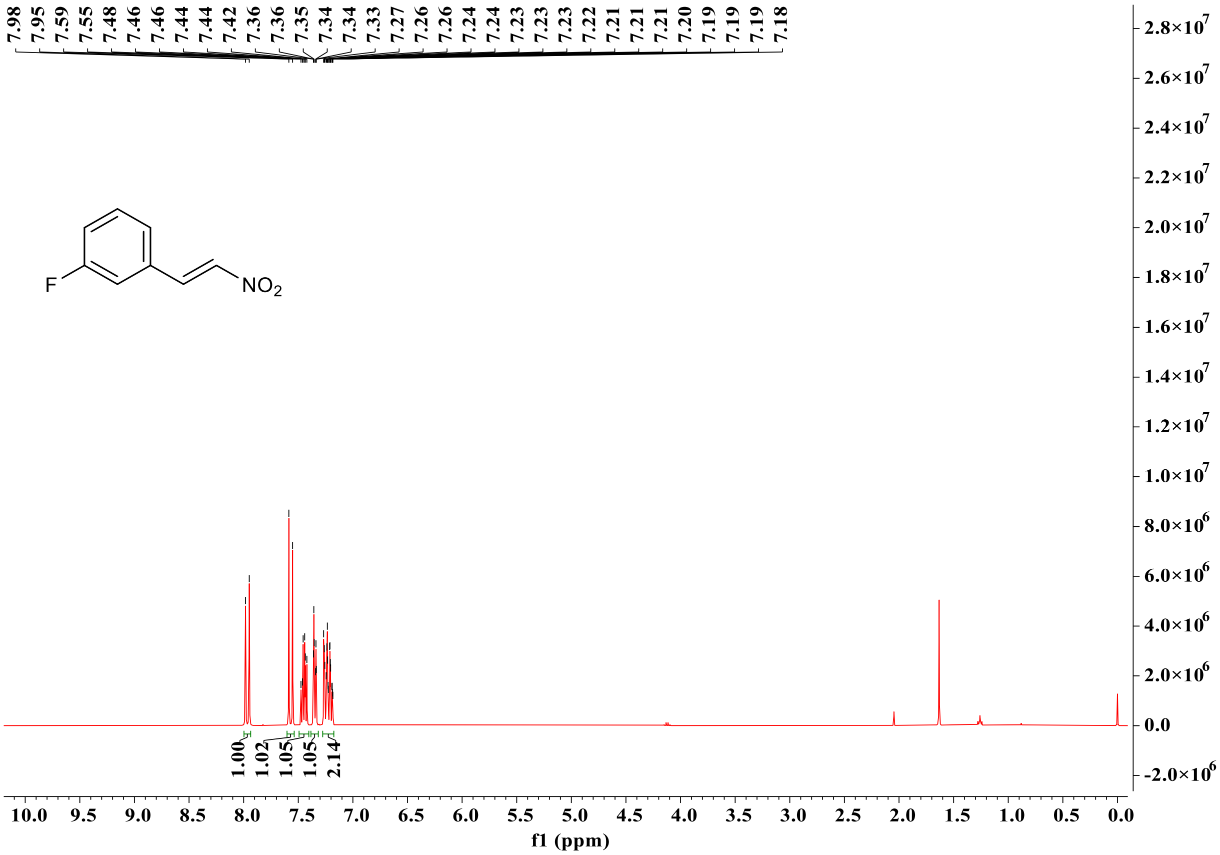

^{1}H NMR (400 MHz, $CDCl_3$) spectrum of compound 16

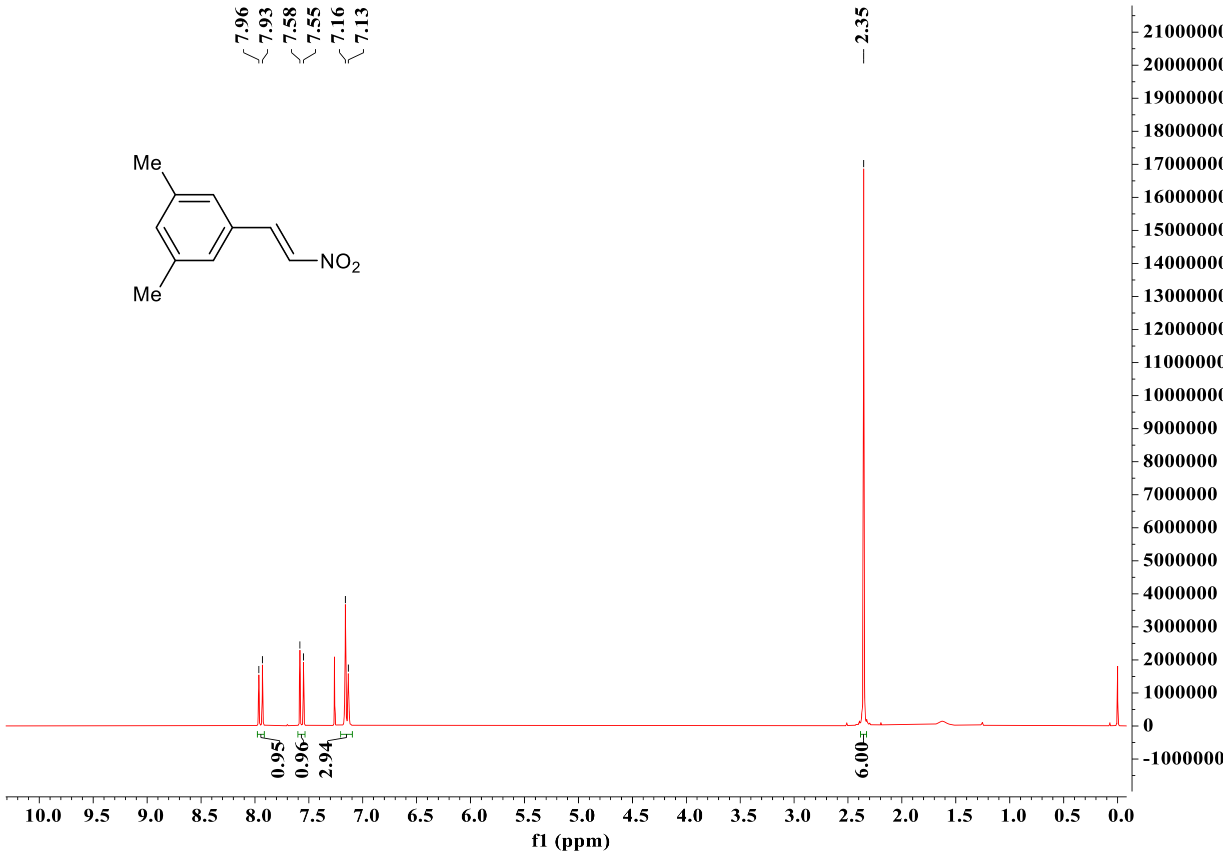

^{1}H NMR (400 MHz, $CDCl_3$) spectrum of compound 17

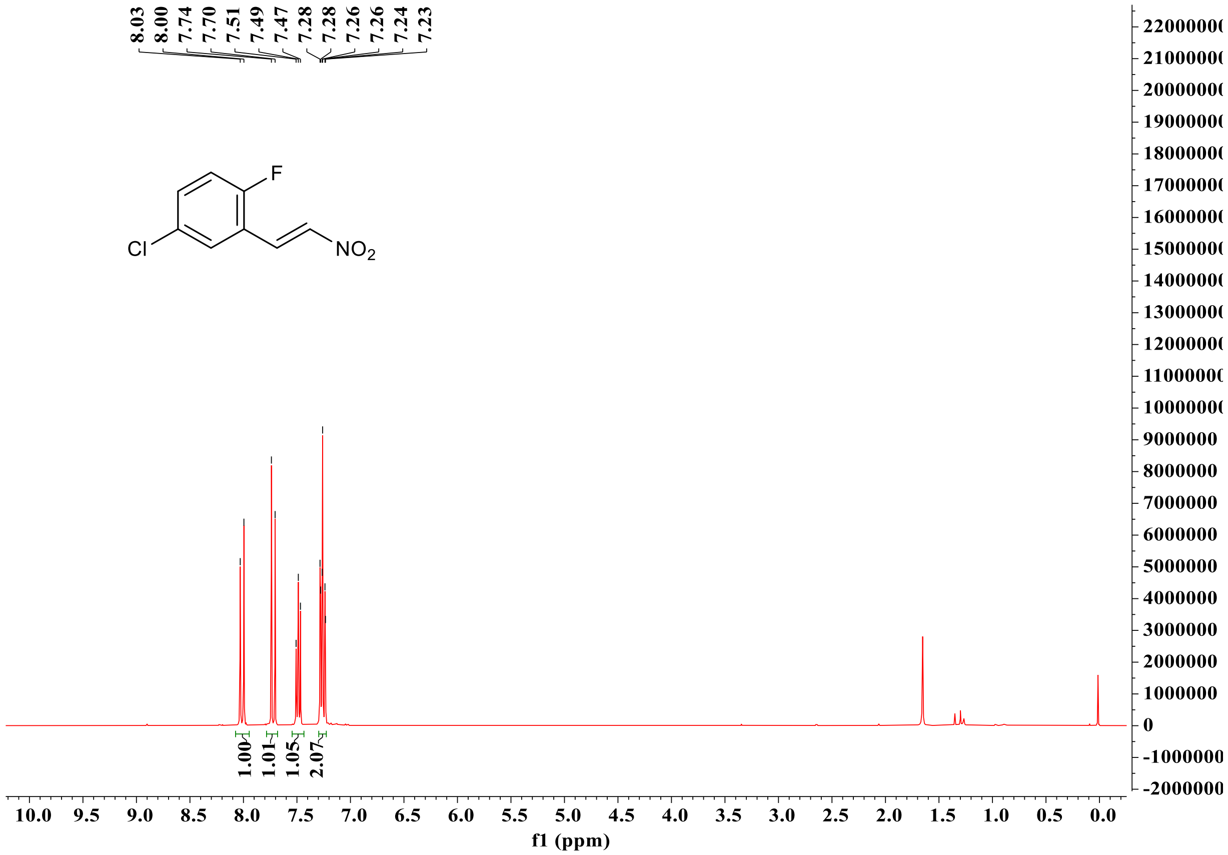

^{1}H NMR (400 MHz, $CDCl_3$) spectrum of compound 18

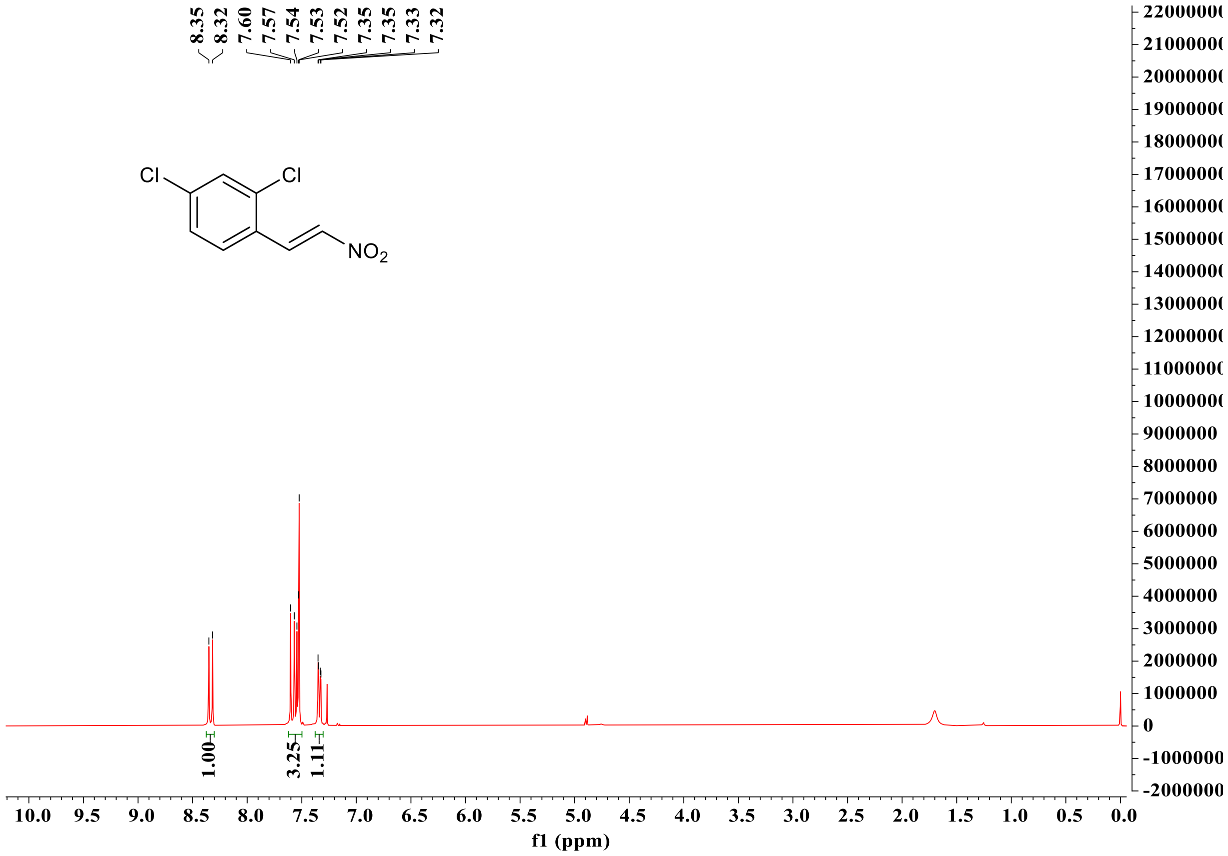

^{1}H NMR (400 MHz, $CDCl_3$) spectrum of compound 19

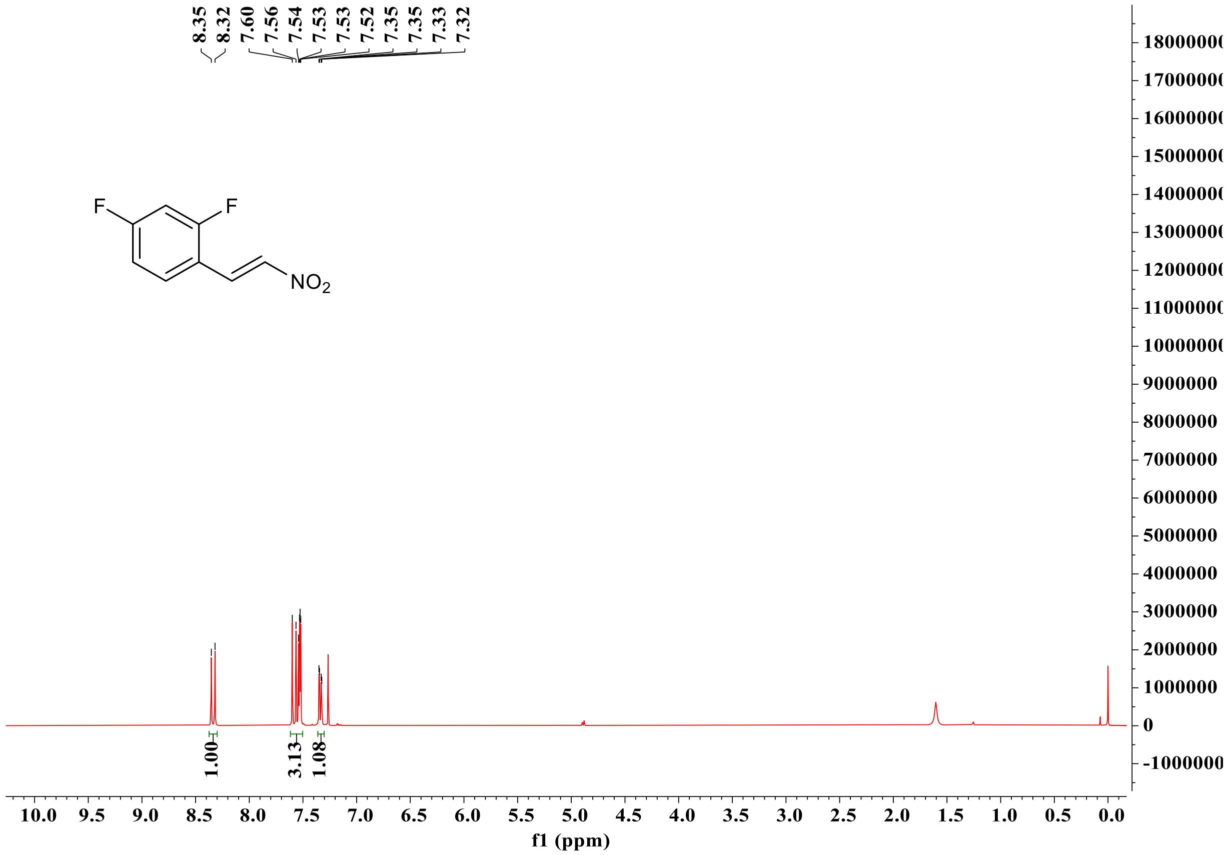

^{1}H NMR (400 MHz, $CDCl_3$) spectrum of compound 20

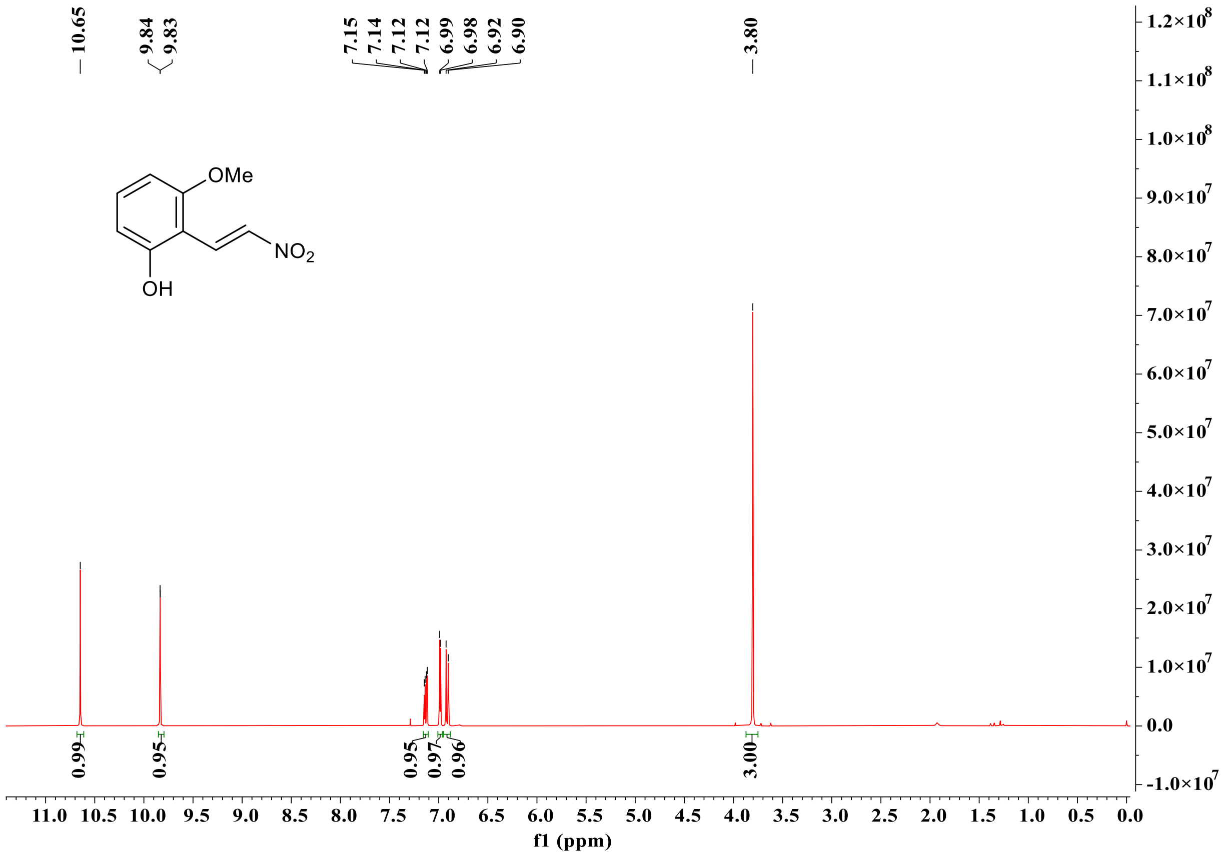

^1H NMR (400 MHz, $CDCl_3$) spectrum of compound 21

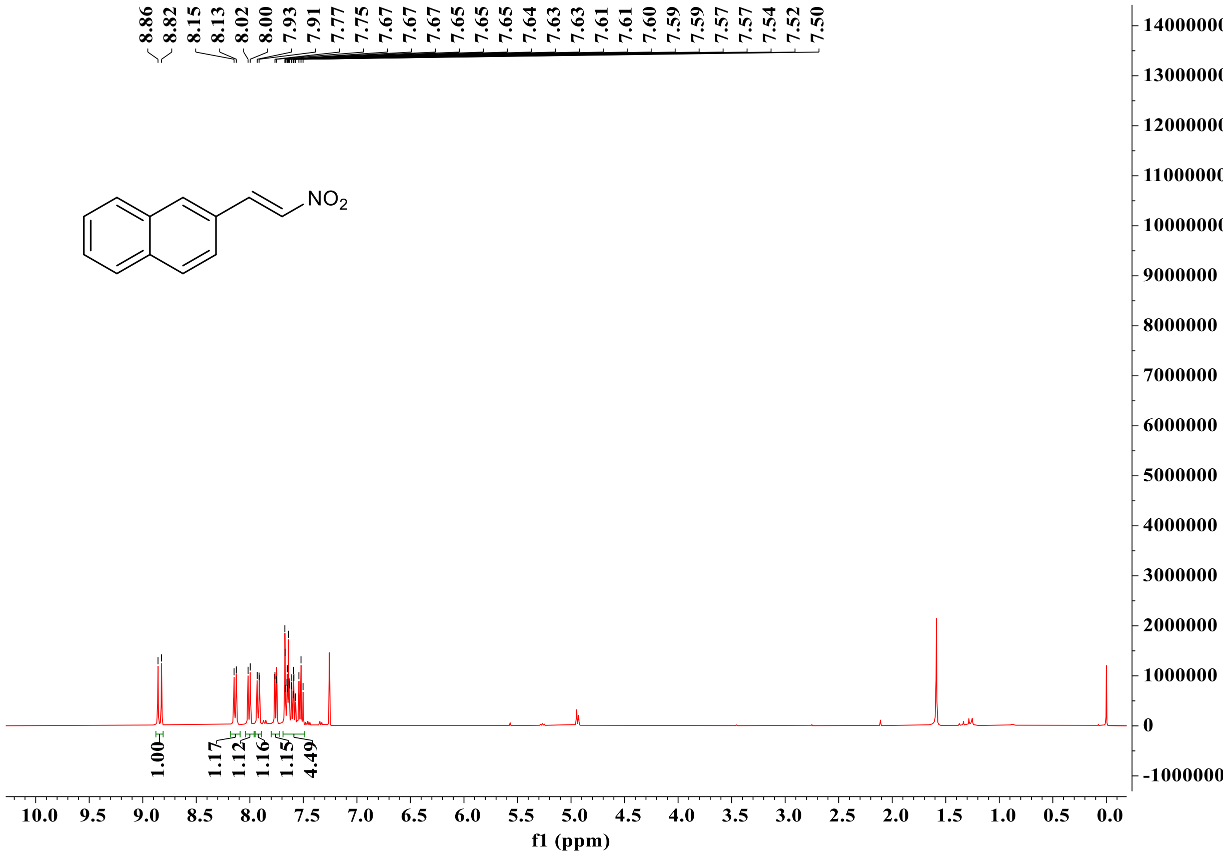

^1H NMR (400 MHz, $CDCl_3$) spectrum of compound 22

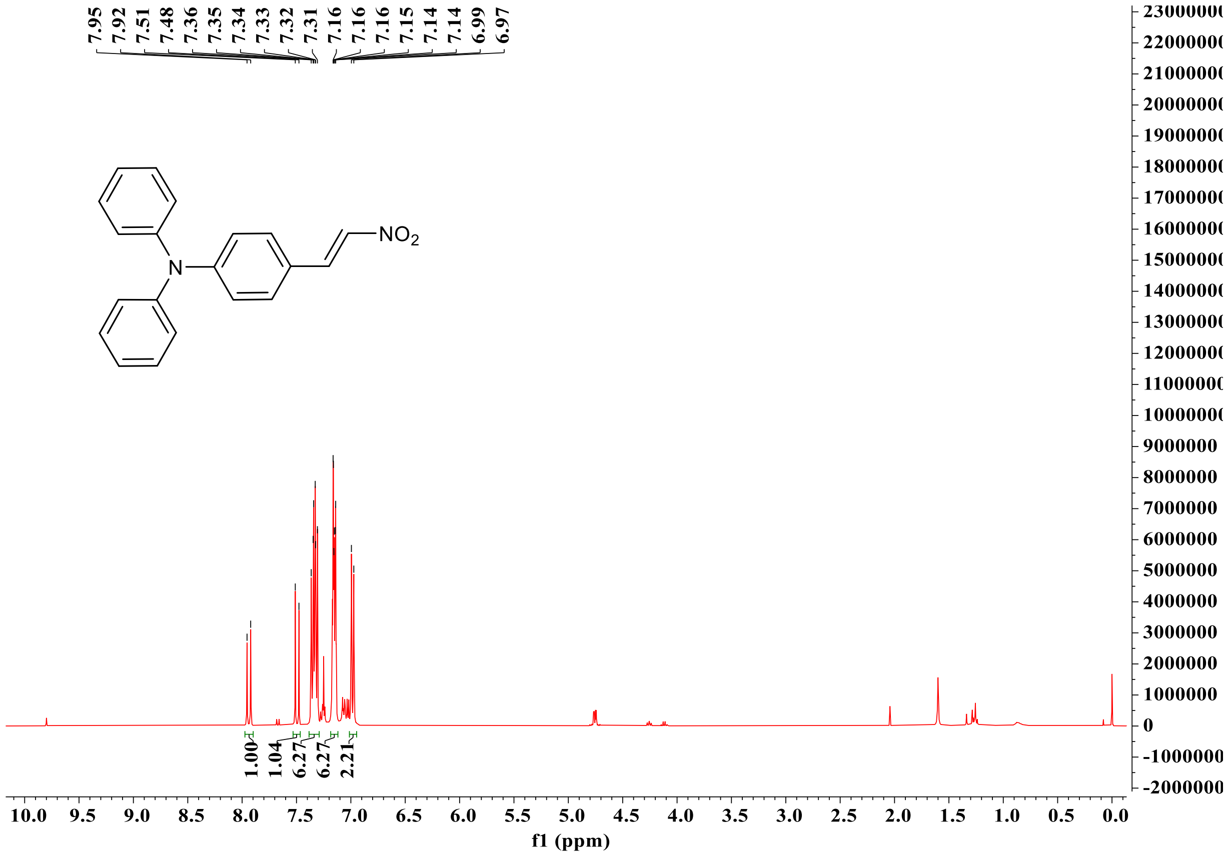

^{1}H NMR (400 MHz, $CDCl_3$) spectrum of compound 23

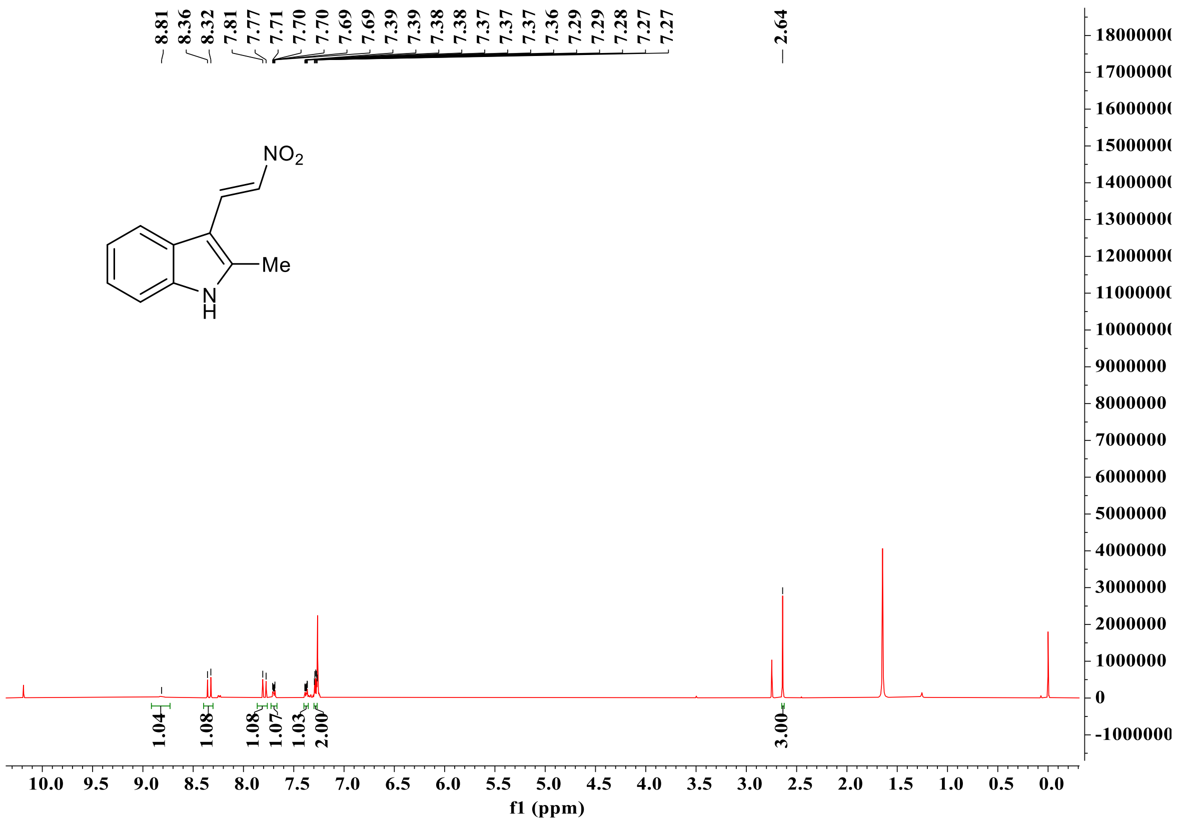

^{1}H NMR (400 MHz, $CDCl_3$) spectrum of compound 24

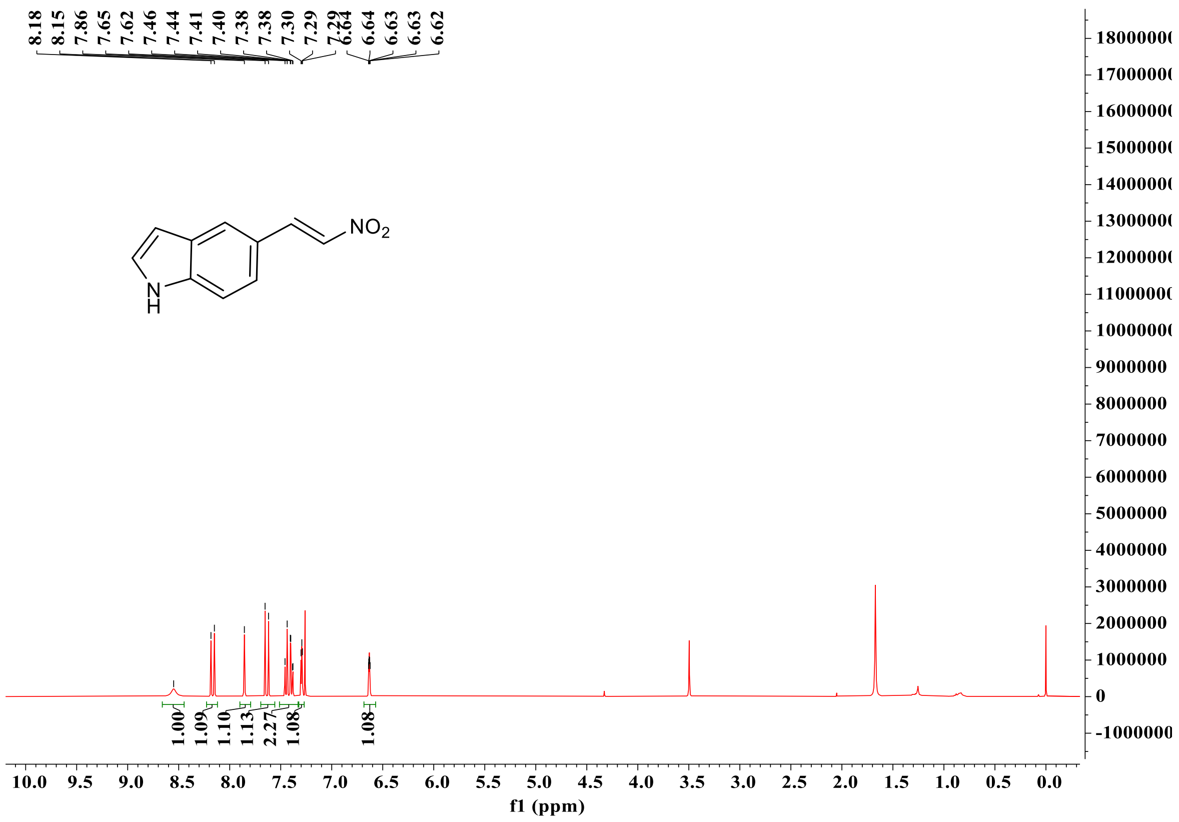

^1H NMR (400 MHz, $CDCl_3$) spectrum of compound 25

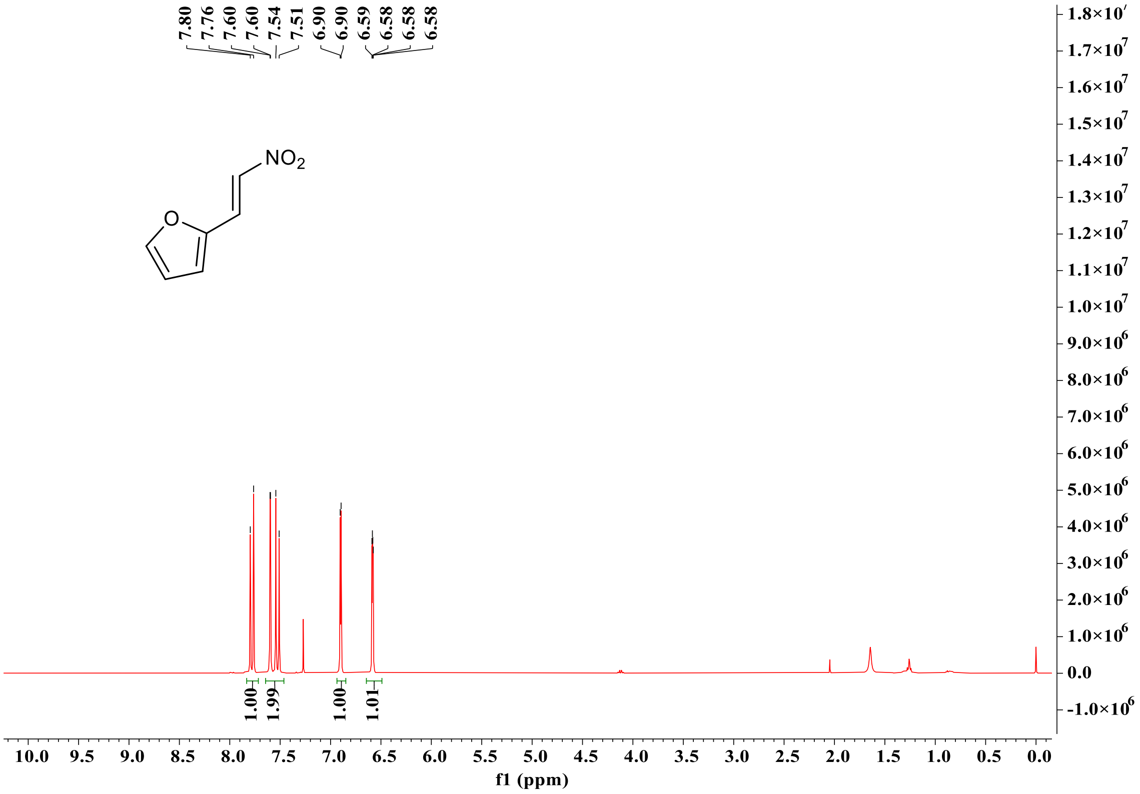

^1H NMR (400 MHz, $CDCl_3$) spectrum of compound 26

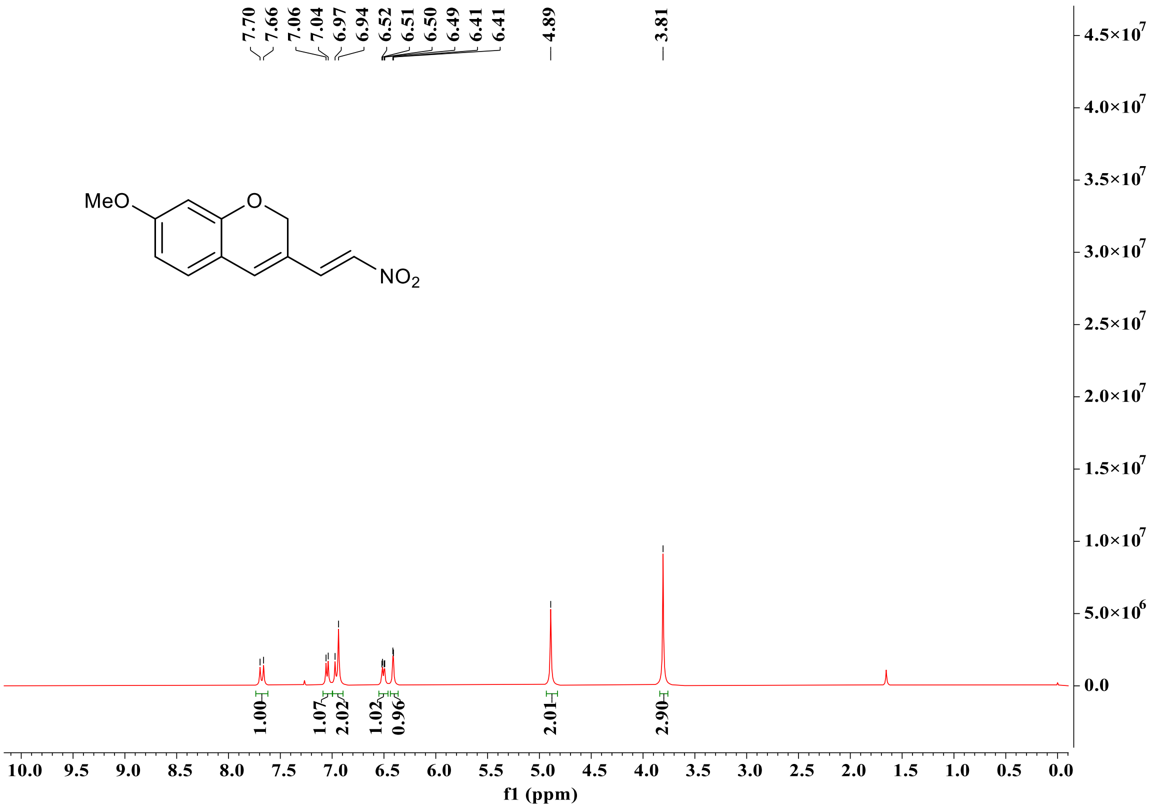

^{13}C NMR (100 MHz, $CDCl_3$) spectrum of compound 26

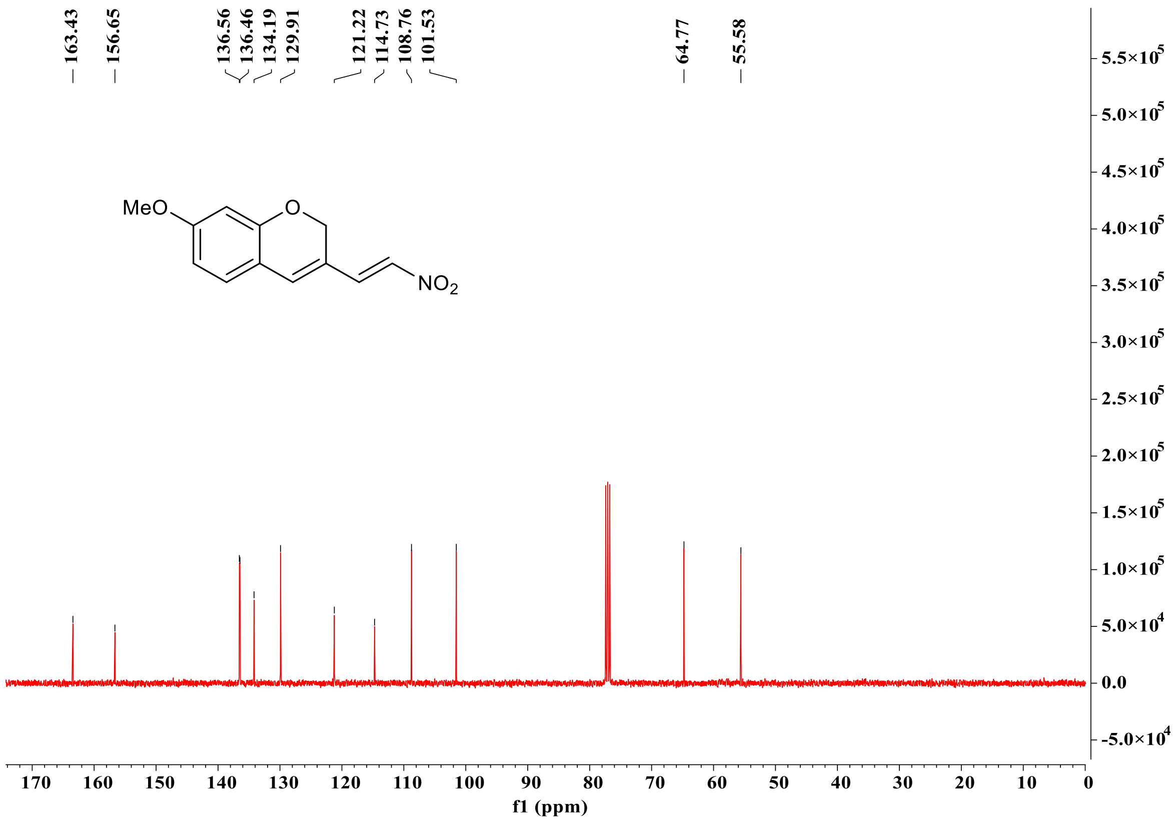

^{1}H NMR (400 MHz, $CDCl_3$) spectrum of compound 27

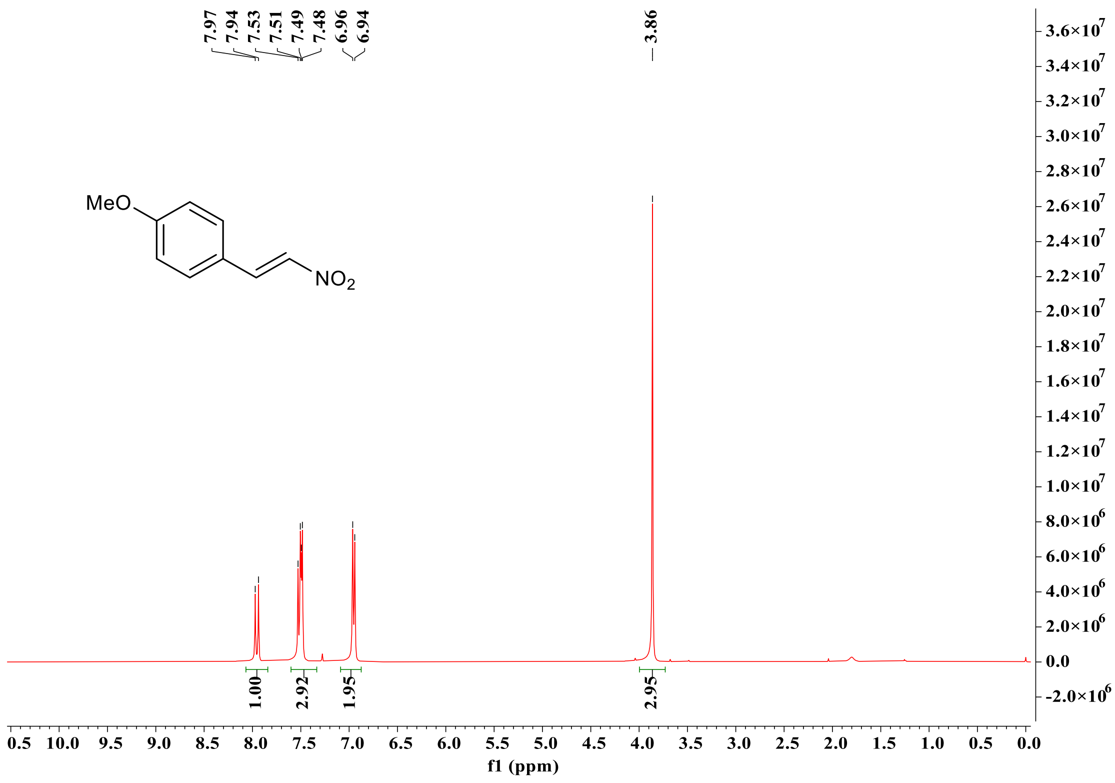

^1H NMR (400 MHz, $CDCl_3$) spectrum of compound 28

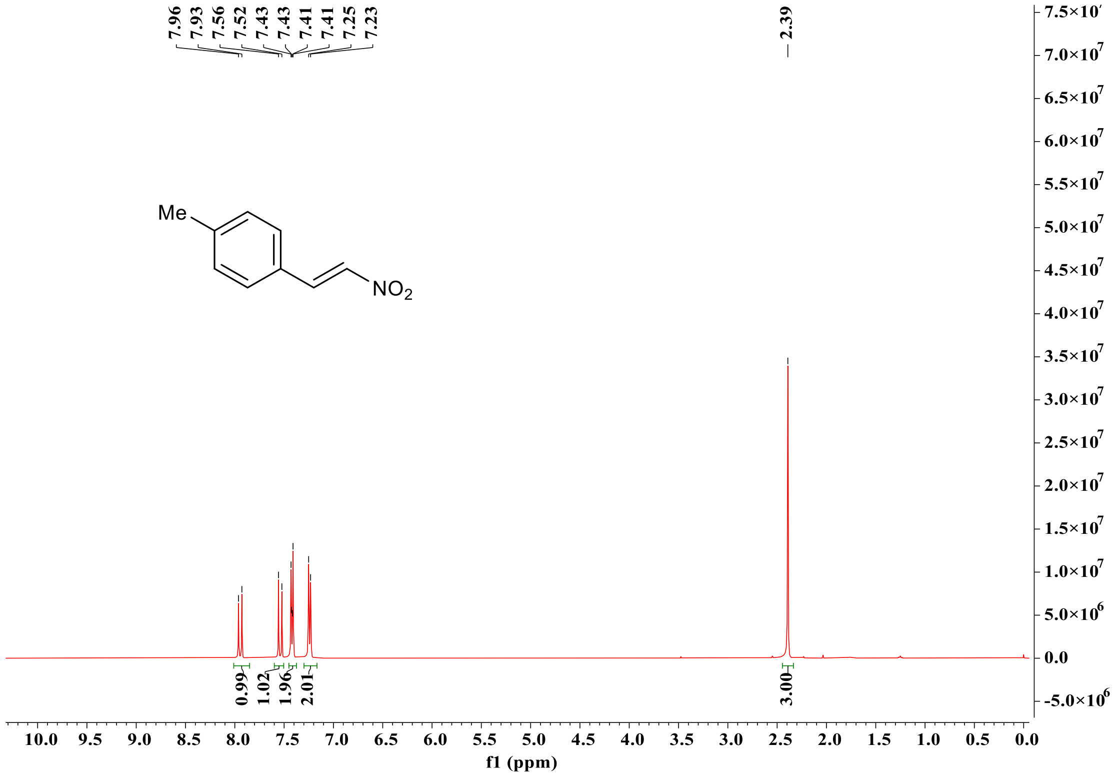

^{13}C NMR (100 MHz, $CDCl_3$) spectrum of compound 28

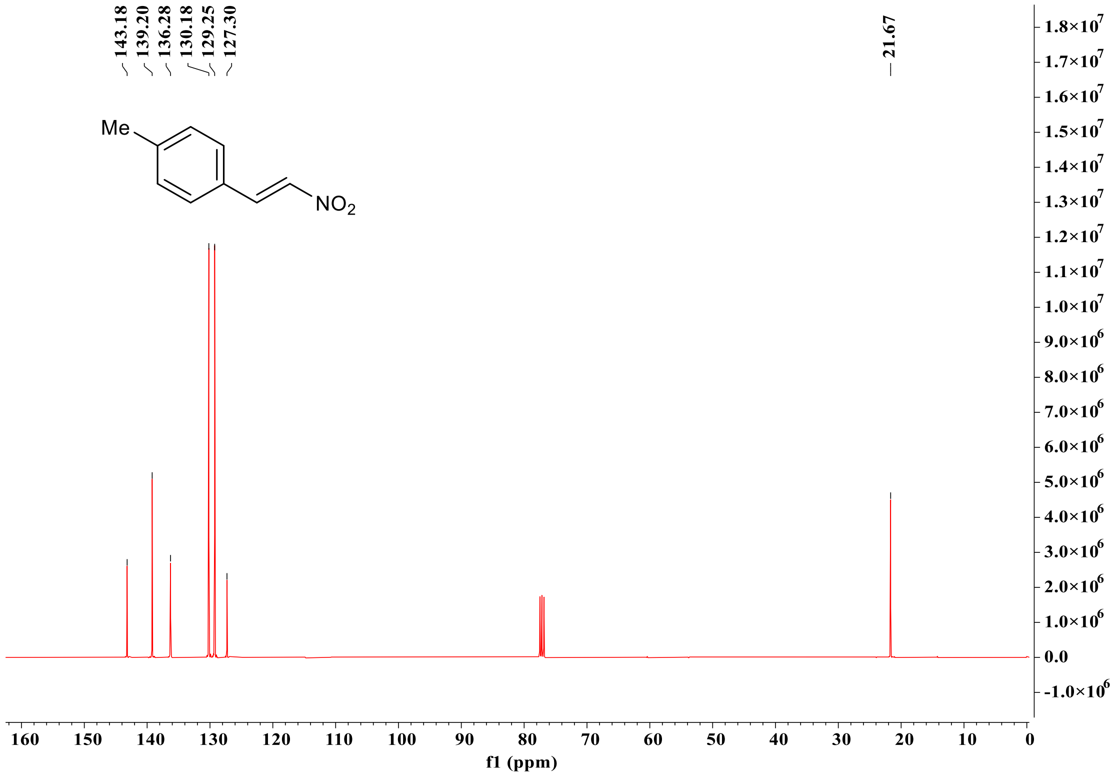

^{1}H NMR (400 MHz, CDCl3) spectrum of compound 29

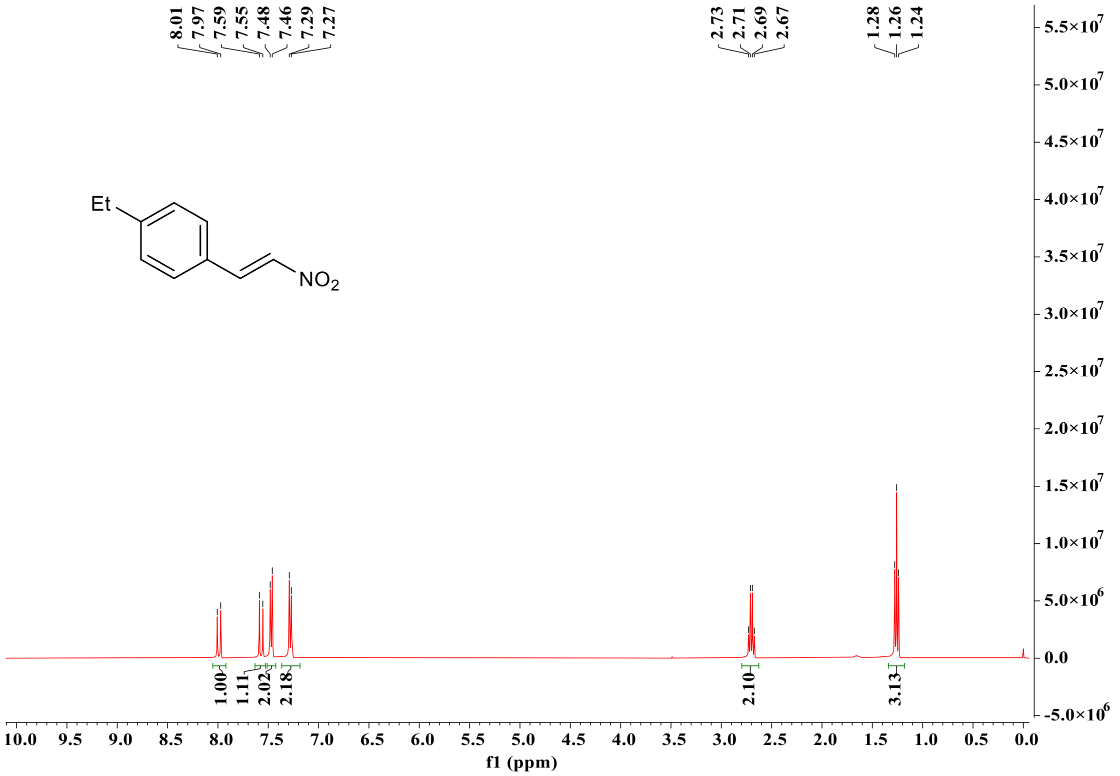

^{1}H NMR (400 MHz, CDCl3) spectrum of compound 30

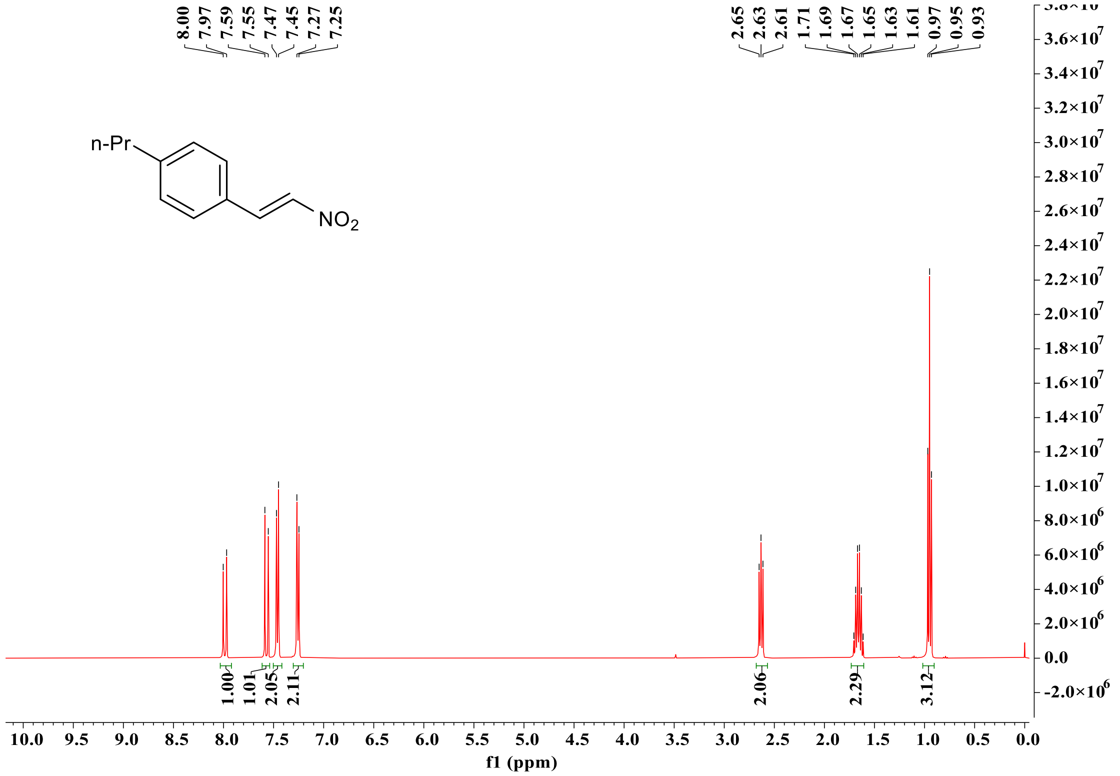

^1H NMR (400 MHz, $CDCl_3$) spectrum of compound 31

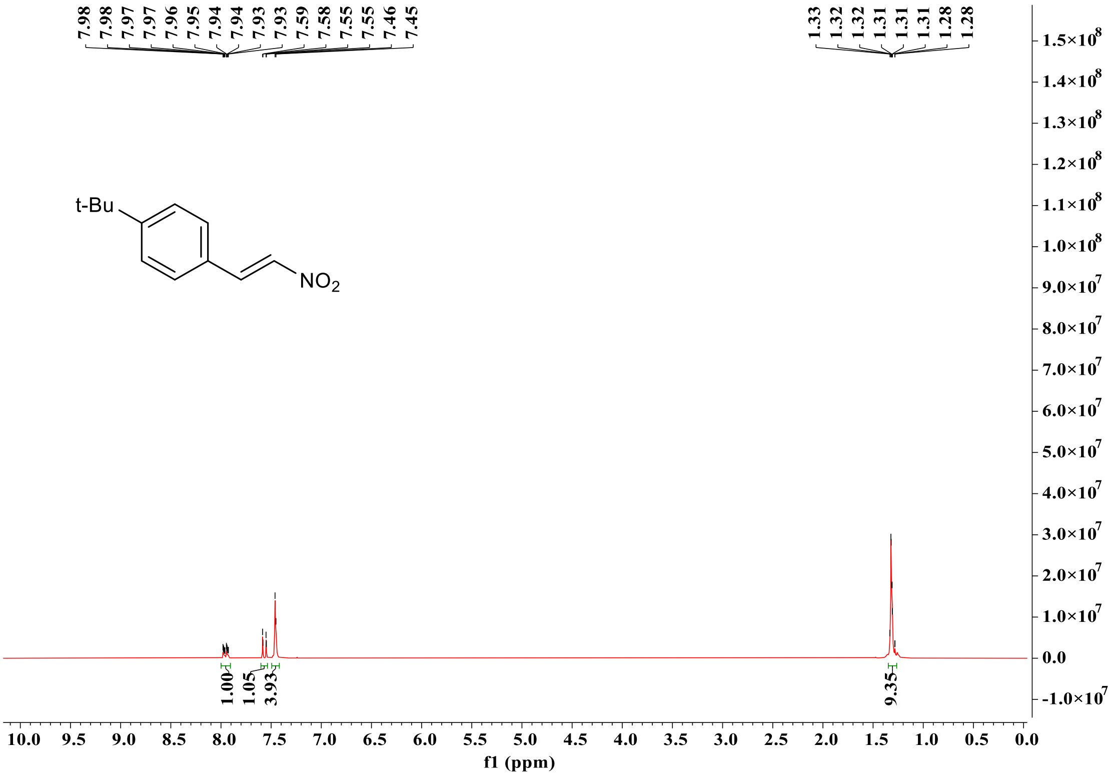

^1H NMR (400 MHz, $CDCl_3$) spectrum of compound 32

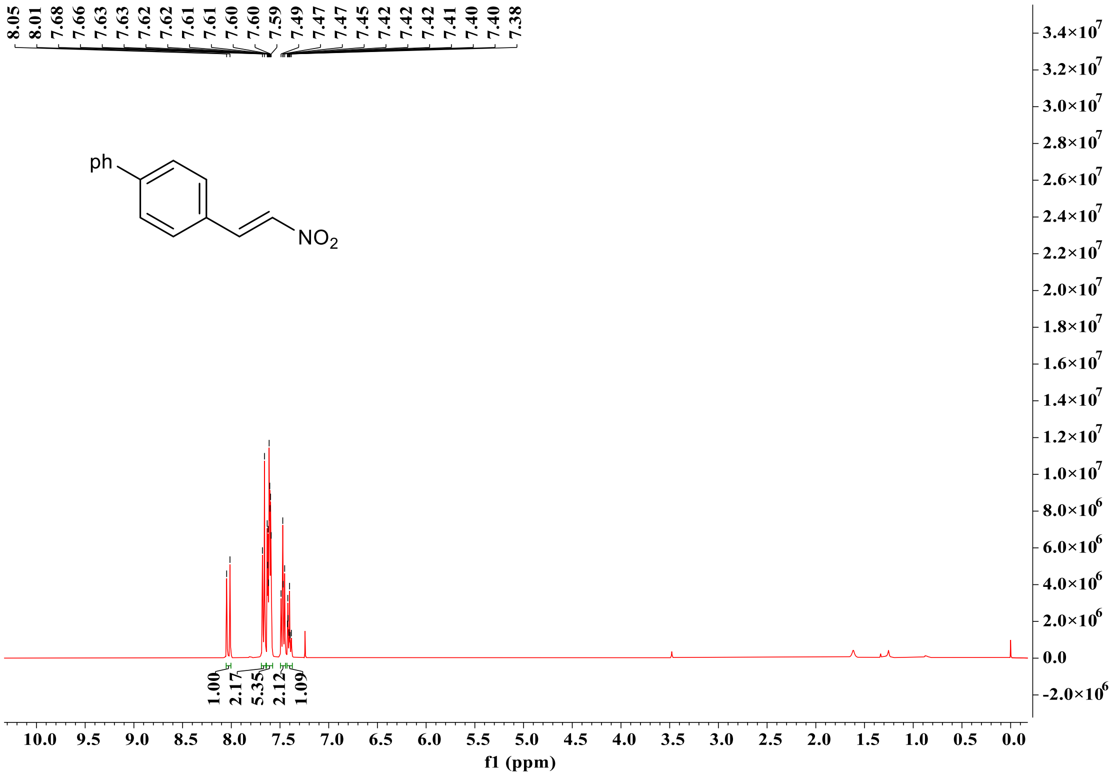

^{1}H NMR (400 MHz, $CDCl_3$) spectrum of compound 33

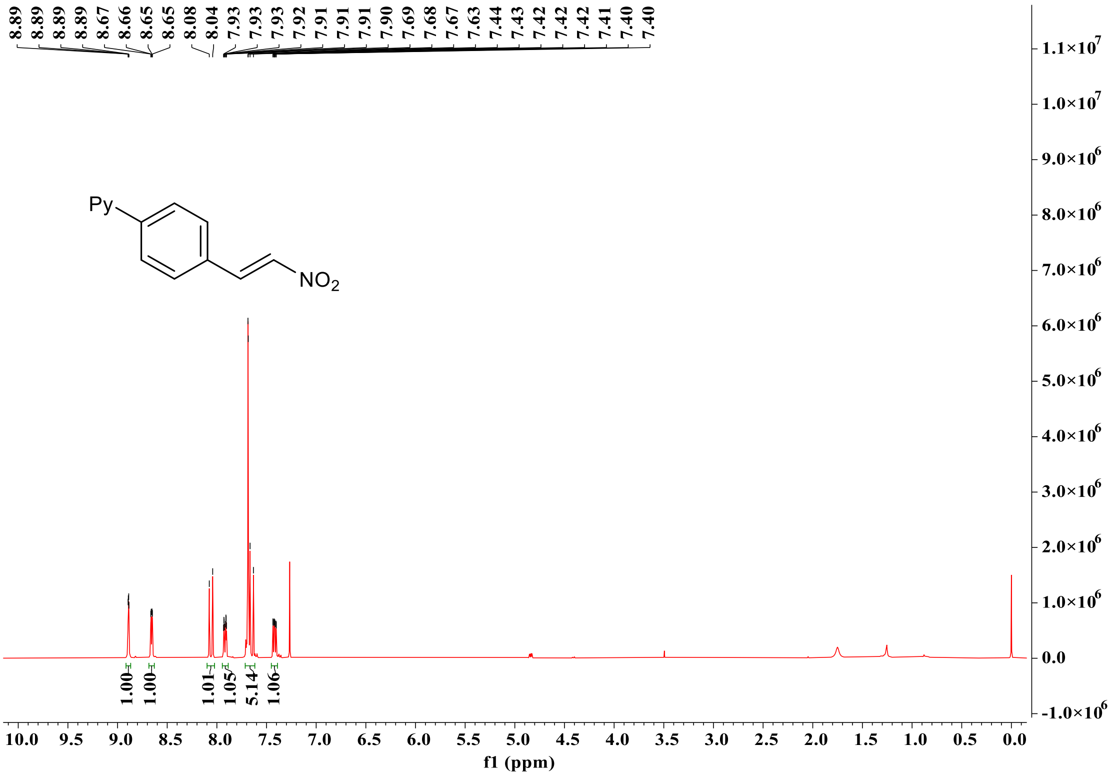

11. X-ray crystallographic data

X-ray Crystallographic Data of compound 26 (ccdc: 2179910)

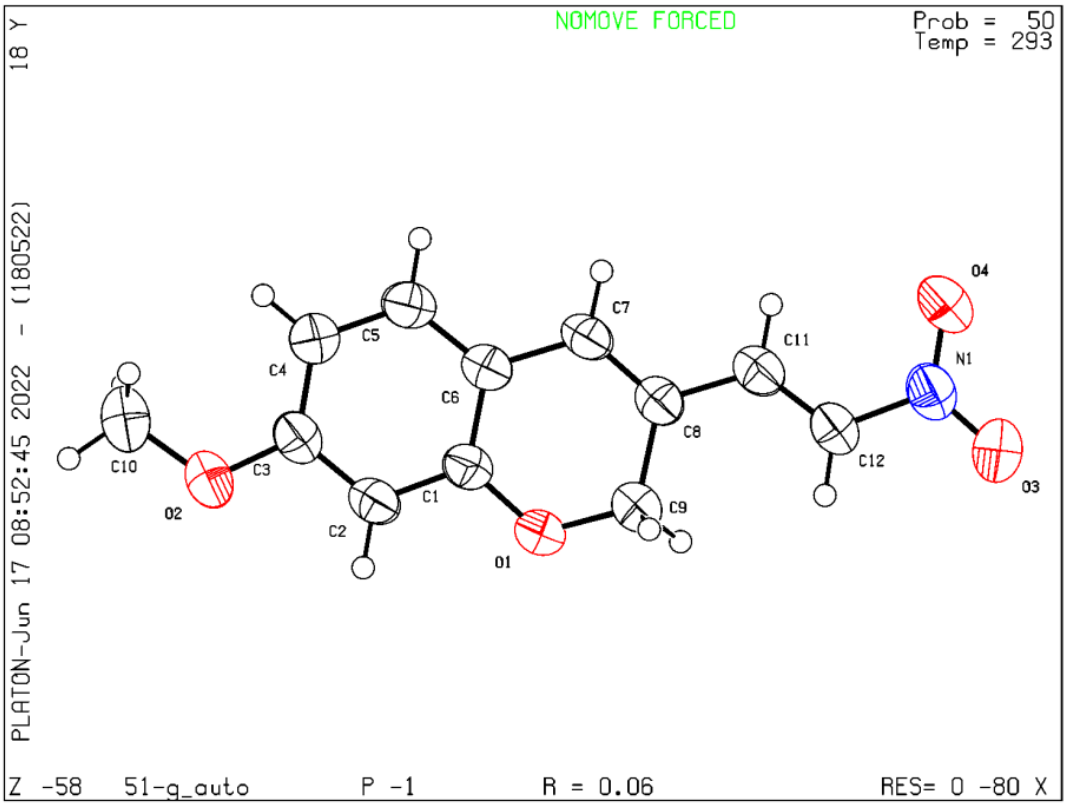

Table S6. Crystal data and structure refinement of compound 26.

Identification code	26
Empirical formula	$C_{12}H_{11}NO_4$
Formula weight	233.22
Temperature/K	293(2)
Crystal system	triclinic
Space group	P-1
a/Å	6.6046(7)
b/Å	7.5090(8)
c/Å	11.8157(8)
α/	75.493(7)
β/	75.532(7)
γ/	84.677(8)
Volume/Å^3	549.03(9)
Z	2
ρ_{calc}g/cm^3	1.441
μ/mm^{-1}	0.108
F(000)	249.0
Crystal size/mm^3	0.14 × 0.12 × 0.11
Radiation	Mo Kα (λ = 0.71073)
2Θ range for data collection/	6.564 to 52.746
Index ranges	-8 ≤ h ≤ 8, -6 ≤ k ≤ 9, -14 ≤ l ≤ 14
Reflections collected	4580
Independent reflections	2242 [R_{int} = 0.0336, R_{sigma} = 0.0477]
Data/restraints/parameters	2242/0/156
Goodness-of-fit on F^2	1.017
Final R indexes [I>=2σ (I)]	R_1 = 0.0622, wR_2 = 0.1549
Final R indexes [all data]	R_1 = 0.1087, wR_2 = 0.1983
Largest diff. peak/hole / e Å^{-3}	0.25/-0.22

Table S7. Fractional atomic coordinates (×10^4) and equivalent isotropic displacement parameters (Å^2×10^3) of compound 26. U_{eq} is defined as 1/3 of the trace of the orthogonalised U_{IJ} tensor.

Atoms	*x*	*y*	*z*	U(eq)
O1	2120(2)	2413(3)	5585.2(13)	68.1(6)
O2	969(2)	5943(3)	1949.2(13)	70.5(6)
O4	9609(3)	-1253(3)	8078.6(15)	85.0(7)
O3	7052(3)	-1597(3)	9642.4(16)	92.8(8)
N1	7769(3)	-1016(3)	8562.9(18)	64.2(6)
C1	2983(3)	3275(3)	4423.9(18)	47.8(6)
C8	5674(3)	1524(3)	5895.2(18)	47.5(6)
C6	5129(3)	3099(3)	3926.1(18)	45.8(6)
C2	1651(3)	4218(3)	3738.2(18)	53.3(6)
C3	2417(3)	5012(3)	2531.4(19)	51.5(6)
C7	6436(3)	2111(3)	4714.0(19)	50.8(6)
C11	6974(4)	577(3)	6685(2)	52.2(6)
C9	3403(3)	1894(4)	6428.9(19)	57.4(7)
C4	4531(4)	4848(4)	2011(2)	58.4(7)
C12	6356(4)	-56(4)	7855(2)	57.4(7)
C5	5854(4)	3892(4)	2714.1(19)	57.8(7)
C10	1587(5)	6671(5)	684(2)	83.9(9)

Table S8. Anisotropic displacement parameters ($Å^2×10^3$) of compound 26. The anisotropic displacement factor exponent takes the form: -$2\pi^2[h^2a^{*2}U_{11}+2hka^*b^*U_{12}+\ldots]$.

Atoms	U_{11}	U_{22}	U_{33}	U_{23}	U_{13}	U_{12}
O1	39.7(9)	95.2(15)	56.7(10)	-1.5(8)	-7.9(7)	7.4(9)
O2	59.4(11)	89.1(15)	61.9(10)	-11.2(9)	-25.8(8)	16.8(10)
O4	53.8(11)	105.5(18)	85.6(13)	-2.6(11)	-24.5(10)	15.4(11)
O3	82.6(14)	119.2(19)	60.0(12)	8(1)	-21.3(9)	12.8(12)
N1	56.7(13)	65.9(15)	67.1(13)	-4.6(10)	-23.0(11)	5.8(11)
C1	40.7(12)	50.2(14)	52.2(12)	-14.5(10)	-9.6(9)	3.7(10)
C8	41.9(12)	44.5(14)	56.6(13)	-9.7(10)	-15.3(10)	-0.4(10)
C6	39.4(11)	43.3(13)	54.9(12)	-12.8(10)	-10.4(9)	-0.4(10)
C2	39.4(11)	62.1(16)	58.4(14)	-16.5(11)	-12.2(10)	7.0(11)
C3	49.0(13)	51.8(15)	57.7(13)	-15.8(11)	-20.1(10)	6.7(11)
C7	35.8(11)	51.7(15)	63.1(14)	-11.3(11)	-12.2(10)	2.4(10)
C11	44.6(12)	48.6(15)	63.5(14)	-11.9(11)	-15.7(10)	3.2(11)
C9	47.3(13)	66.5(17)	56.6(13)	-11.9(11)	-14.6(10)	6.8(12)
C4	52.2(14)	64.0(17)	53.0(12)	-6.2(11)	-10.9(10)	2.7(12)
C12	47.2(13)	62.2(17)	61.2(14)	-8.4(11)	-19.6(11)	7.5(12)
C5	42.8(12)	63.4(17)	61.0(14)	-9.9(11)	-6.6(10)	1.4(12)
C10	80.5(19)	102(3)	66.6(17)	-5.2(15)	-33.1(14)	14.4(17)

Table S9. Bond lengths of compound 26.

Atoms	Atoms	Length/Å	Atoms	Atoms	Length/Å
O1	C1	1.364(3)	C8	C7	1.333(3)
O1	C9	1.425(3)	C8	C11	1.438(3)
O2	C3	1.356(3)	C8	C9	1.504(3)
O2	C10	1.424(3)	C6	C7	1.449(3)
O4	N1	1.224(2)	C6	C5	1.385(3)
O3	N1	1.224(2)	C2	C3	1.384(3)
N1	C12	1.433(3)	C3	C4	1.386(3)
C1	C6	1.400(3)	C11	C12	1.316(3)
C1	C2	1.373(3)	C4	C5	1.385(3)

Table S10. Bond Angles of compound 26.

Atoms	Atoms	Atoms	Angle/˚	Atoms	Atoms	Atoms	Angle/˚
C1	O1	C9	119.61(16)	C5	C6	C1	117.5(2)
C3	O2	C10	118.76(19)	C5	C6	C7	124.63(19)
O4	N1	O3	122.6(2)	C1	C2	C3	120.2(2)
O4	N1	C12	119.9(2)	O2	C3	C2	115.07(19)
O3	N1	C12	117.5(2)	O2	C3	C4	124.9(2)
O1	C1	C6	121.1(2)	C2	C3	C4	120.0(2)
O1	C1	C2	117.52(19)	C8	C7	C6	121.64(19)
C2	C1	C6	121.2(2)	C12	C11	C8	125.9(2)
C7	C8	C11	121.84(19)	O1	C9	C8	115.03(16)
C7	C8	C9	119.46(19)	C5	C4	C3	119.2(2)
C11	C8	C9	118.69(18)	C11	C12	N1	121.7(2)
C1	C6	C7	117.84(19)	C6	C5	C4	122.0(2)

Table S11. Hydrogen Atom Coordinates (Å×10^4) and Isotropic Displacement Parameters (Å^2×10^3) of compound 26.

Atoms	*x*	*y*	*z*	U(eq)
H2	230.42	4322.56	4085.54	64
H7	7840.91	1879.44	4387.69	61
H11	8380.97	394.96	6335.54	63
H9A	2850.06	795.88	7019.67	69
H9B	3305.44	2866.53	6849.25	69
H4	5054.35	5374.32	1199.14	70
H12	4964.03	124.34	8233.74	69
H5	7271.12	3778.57	2361.99	69
H10A	2013.94	5680.59	287.86	126
H10B	2733.75	7473.22	505.66	126
H10C	430.7	7351.16	405.27	126
